# Sink/Source Balance of Leaves Influences Amino Acid Pools and Their Associated Metabolic Fluxes in Winter Oilseed Rape (*Brassica napus* L.)

**DOI:** 10.3390/metabo10040150

**Published:** 2020-04-13

**Authors:** Younès Dellero, Maud Heuillet, Nathalie Marnet, Floriant Bellvert, Pierre Millard, Alain Bouchereau

**Affiliations:** 1 Department Plant Biology and Breeding, Agrocampus Ouest, Institute for Genetics, Environment and Plant Protection, French National Research Institute for Agriculture, Food and Environment, University of Rennes II, 35653 Le Rheu, France; alain.bouchereau@univ-rennes1.fr; 2 Department Plant Biology and Breeding, Department Microbiology and Food Chain, INSA, TBI, French National Center for Scientific Research, French National Research Institute for Agriculture, Food and Environment, University of Toulouse, 31400 Toulouse, France; heuillet@insa-toulouse.fr (M.H.); fbellvert@insa-toulouse.Fr (F.B.); millard@insa-toulouse.fr (P.M.); 3 MetaToul-MetaboHUB, National Infrastructure of Metabolomics and Fluxomics, 33140 Toulouse, France; 4 Department Plant Biology and Breeding and Department Transform, Agrocampus Ouest, Plateau de Profilage Métabolique et Métabolique (P2M2), Biopolymers Interactions Assemblies, Institute for Genetics, Environment and Plant Protection, French National Research Institute for Agriculture, Food and Environment, University of Rennes II, 35653 Le Rheu, France; nathalie.marnet@inra.fr

**Keywords:** metabolomics, isotopically non-stationary steady-state metabolic flux analysis, *Brassica napus*, winter oilseed rape, sink/source balance, senescence, amino acid metabolism, threonine, valine, proline, SMCSO

## Abstract

Nitrogen remobilization processes from source to sink tissues in plants are determinant for seed yield and their implementation results in a complete reorganization of the primary metabolism during sink/source transition. Here, we decided to characterize the impact of the sink/source balance on amino acid metabolism in the leaves of winter oilseed rape grown at the vegetative stage. We combined a quantitative metabolomics approach with an instationary ^15^N-labeling experiment by using [^15^N]_L_-glycine as a metabolic probe on leaf ranks with a gradual increase in their source status. We showed that the acquisition of the source status by leaves was specifically accompanied by a decrease in asparagine, glutamine, proline and S-methyl-l-cysteine sulphoxide contents and an increase in valine and threonine contents. Dynamic analysis of ^15^N enrichment and concentration of amino acids revealed gradual changes in the dynamics of amino acid metabolism with respect to the sink/source status of leaf ranks. Notably, nitrogen assimilation into valine, threonine and proline were all decreased in source leaves compared to sink leaves. Overall, our results suggested a reduction in de novo amino acid biosynthesis during sink/source transition at the vegetative stage.

## 1. Introduction

Winter oilseed rape (*Brassica napus* L.) is a major oleaginous crop ranked the 3rd most essential source of plant oil in the world. The seeds of *B. napus* are naturally rich in triglycerides and proteins, representing, respectively, 44–48% and 18–21% of the dry weight, with applications for oil and seed cake in human and livestock nutrition [1]. Oilseed rape production is highly demanding for mineral nitrogen (N) inputs (150–250 kg N/ha) compared with other crops, and is characterized by a strong nitrogen uptake efficiency at the vegetative stage [2,3]. However, oilseed rape has a low nitrogen remobilization efficiency (NRE), leading to a low nitrogen use efficiency and a low recovery of initial N input in the seeds (50% of losses) [2,4]. NRE can be described as the proportion of N absorbed by the crop, which is lost during the vegetative stage (due to leaf abscission) and during the reproductive stage (N not present in the seeds) [5,6]. Considering the agroecological transition, it is crucial to maintain crop seed yield and quality with reduced chemical inputs, such as nitrogen. Since oilseed rape breeding progress in the last 20 years has essentially improved nitrogen uptake efficiency, the optimization of nitrogen use efficiency, and more specifically NRE in oilseed rape, is still a promising target for future crop improvement [7,8,9].

Nitrogen is taken up first from the soil through ammonia and nitrate transporters localized in the roots [10]. Then, nitrogen is directly assimilated in roots or transported towards sink and source leaves to be also assimilated by the Glutamine Synthetase/Glutamine: 2-OxoGlutarate AminoTransferase (GS/GOGAT) cycle. Nitrogen remobilization is a complex process, occurring at both vegetative and reproductive stages which implies a transfer of N-containing molecules from source tissues to sink tissues. This transfer of nitrogen can work at both short and long distances (between roots and seeds for example) and generally uses amino acids, ureides or peptides as nitrogen vectors [11]. Recent studies showed that the active transport of amino acids and ureides can be performed by cell-to-cell transporters showing wide or specific substrate preferences [12,13,14]. Nitrogen remobilization in plants is often linked with senescence, i.e., the active remobilization of cell nutrients through the degradation of subcellular compartments (chloroplasts then mitochondria) and cell structures. However, at the vegetative stage in oilseed rape, the activation of nitrogen remobilization processes could already be effective in source leaves (mature but non-senescent). This observation implies that other processes could also be involved in nitrogen remobilization, such as autophagy [5,15]. Indeed, nitrogen remobilization occurs mainly between leaf ranks, with a different sink/source balance at this stage. The acquisition of source status by the leaves is always correlated with a depletion in their chlorophyll and protein contents per leaf area or per dry weight (DW), and consequently with specific modifications to proteolytic activities [16,17,18]. Amino acids produced by protein degradation constitute an important source of carbon and nitrogen that are differentially metabolized by the primary metabolism network depending on the considered amino acids and the cell conditions [19]. In *Arabidopsis*, amino acids are usually converted in Gln, Glu, Asn and Asp by aminotransferases prior to being exported to sink tissues (preferentially transported amino acids) [20]. Thus, the produced deaminated organic acids can enter the TCA cycle and contribute to the mitochondrial respiration. However, both developmental and dark-induced leaf senescence (nitrogen remobilization triggered by shading) can trigger the activation of branched chain amino acid (BCAA) catabolism in the mitochondria (Leu, Ile and Val) [21,22]. Those amino acids are then used as an alternative respiratory substrate for the production of energy by the mitochondrial electron transfer chain [23,24,25,26]. In addition, the catabolism of other amino acids such as Pro and Tyr can also participate in this mitochondrial respiration process during dark-induced senescence [27,28]. Recent work based on ^15^N-proline experiments showed that proline oxidation capacity was weakly affected by the sink/source status of oilseed rape leaves, confirming that developmental and dark-induced leaf senescence use different strategies for nitrogen remobilization [21,22,29]. Overall, the specific regulation of the enzymatic network associated with nitrogen metabolism seems to be crucial to sustaining nitrogen remobilization processes and mitochondrial respiration during senescence.

Developmental and dark-induced leaf senescence induce a complete reprogramming of genome transcription, leading to major modifications to transcript, protein and metabolite levels and, ultimately, metabolic fluxes [21,22,30]. However, modulations in transcript levels are not always associated with similar modifications to protein and metabolite levels [31]. In addition, it is difficult to predict modifications to nitrogen fluxes at the cellular level from instantaneous metabolomics data [32]. Indeed, amino acids are, essentially, metabolic intermediates between proteins and exported nitrogen and their accumulation/depletion is not always correlated with the increase/decrease in the associated metabolic fluxes [29,33]. Therefore, it is crucial to develop both spatial and temporal fluxomic analyses around nitrogen remobilization processes in order to identify metabolic bottlenecks and/or key regulatory points for future crop improvement [34]. Two main types of fluxomic approaches are currently employed in plants to gain information about the dynamic response of a metabolic network to specific conditions: Flux-Balance Analysis (FBA) and Metabolic Flux Analysis (MFA). FBA is a constraint-based approach in which steady-state fluxes are predicted by using optimization algorithms and gives an experimentally bounded solution space [35,36,37]. MFA is an isotopic labeling-based approach in which steady-state fluxes are computed by using stoichiometric balancing of isotopic labeling [38,39]. Until recently, MFA in plant tissues has been limited to the study of seeds or cell suspensions that exhibit extended metabolic steady states during which isotopic labeling can approach equilibrated values [40,41,42,43]. Recent improvements in the modelling of transient labels, such as Isotopically Non-Stationary MFA (INST-MFA) by using ^13^C-substrates, can assess short-lived metabolic events that are relevant to most cell types [44,45,46,47].

Here, we decided to characterize modifications in nitrogen fluxes occurring at cellular level within amino acid metabolism during nitrogen remobilization processes in oilseed rape leaves. To do so, we combined both metabolomic and ^15^N-labeling approaches dedicated to the analysis of the amino acid metabolism of winter oilseed rape leaves having a different sink/source balance. We succeeded in identifying amino acid variations that were correlated with the acquisition of the source status in leaves. We also conducted a short-term labeling kinetic with [^15^N]_L_-glycine on the different leaf nodal ranks, and locally estimate some metabolic fluxes using ScalaFlux [48], a recent extension of the ^15^N-INST-MFA framework.

## 2. Results

### 2.1. S-methyl-l-cysteine Sulphoxide (SMCSO), Gln, Pro, Asn, Val and Thr Contents Are Specifically Impacted by the Acquisition of the Source Status of Winter Oilseed Rape Leaves

**Figure 1:**
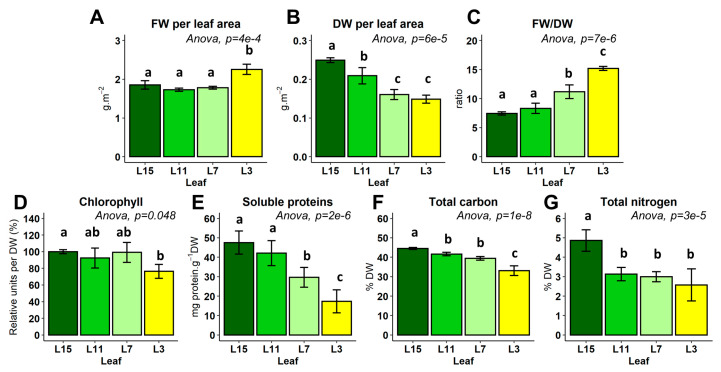
Physiological indicators of the sink/source status of four leaf ranks (L15, L11, L7, L3) from oilseed rape plants grown at the vegetative stage. (**A**) Fresh weight (FW) per leaf area; (**B**) Dry weight (DW) per leaf area; (**C**) Ratio of fresh weight over dry weight; (**D**) Relative chlorophyll content; (**E**) Soluble protein content; (**F**) Total carbon content; (**G**) Total nitrogen content. Plants were grown for 60 days after sowing and developed 16 leaf ranks annotated from the bottom to the top, accordingly (L1 to L16) and the two oldest leaves had already fallen off (L1 and L2). Values are the mean ± SD of 3–5 independent biological replicates. Different letters indicate groups of mean values that are significantly different between the different leaf ranks (ANOVA-Tukey HSD, *p*-value < 0.05).

In order to characterize modifications to nitrogen fluxes occurring at cellular level within amino acid metabolism during nitrogen remobilization in oilseed rape leaves, we decided first to quantify changes in physiology and amino acid pools that occurred during the acquisition of the source status in leaves. *Brassica napus* plants were grown for 60 days after sowing and have reached a 16 leaf nodal ranks stage (annotated from L1 to L16 from the bottom to the top) under our growth conditions. The two oldest yellowing leaves (L1 and L2) have already fallen off, confirming that senescence-associated nutrient remobilization processes were also operating between the different leaf ranks. To simplify our analysis, we selected four leaf ranks (L15, L11, L7 and L3) based on their order of appearance in the plant (young, pre- and post-mature and old leaves) and their visual color, and we quantified their chlorophyll, protein and total nitrogen contents. Since nutrient remobilization processes are known to affect the contents of both fresh weight (FW) and dry weight (DW), we first analyzed these parameters in order to find a good way to normalize our data when comparing the different leaf ranks. While FW per leaf area was relatively constant between the leaf ranks (excepted for L3, which showed minor changes), DW per leaf area was significantly reduced in the oldest leaves, which resulted in a significant increase in the FW/DW ratio (Figure 1A–C). The decrease in DW per leaf area confirmed that nutrient remobilization processes were occurring between the leaf ranks. Consequently, all measured data in the study were normalized per dry weight content. Somewhat surprisingly, chlorophyll content per dry weight was not significantly changed between the different leaf ranks, whereas this was the case by normalizing chlorophyll content per leaf area (Figure 1D). This result confirmed the importance of normalizing data with the DW and highlighted that chlorophyll degradation was over-activated compared with nutrient remobilization processes in L3 leaves only (senescent leaves). Interestingly, protein content and carbon content per DW were both significantly and gradually reduced between the leaf ranks (Figure 1E,F), illustrating the regulation and the activation of remobilization processes. Nitrogen content per DW was only higher in L15 leaves compared with L11, L7 and L3 leaves (Figure 1G). However, the decrease in DW and protein contents between L11, L7 and L3 leaves confirmed that nitrogen was still remobilized in these leaves. Overall, our results showed that the chosen leaf ranks had a gradually advanced source status, i.e., a gradual increase in their nitrogen remobilization processes. Moreover, since the strong activation of chlorophyll degradation in old leaves is always associated with a decrease in chloroplast volume and chloroplast size [49], our result suggested that L3 leaves were at the onset of senescence.

**Figure 2:**
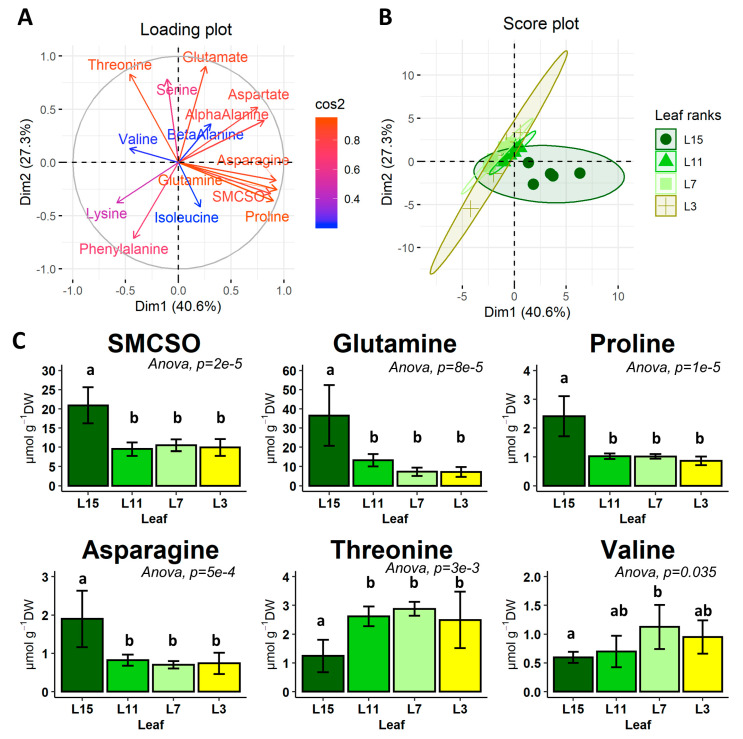
Principal Component Analysis of free amino acid contents from four leaf ranks (L15, L11, L7, L3) of oilseed rape plants grown at the vegetative stage. (**A**) Loading plot with Pearson correlation coefficients for each amino acid along the two major axis of the PCA; (**B**) Score plot of leaf groups along the two major axes of the PCA with 95% confidence ellipses; (**C**) Content of amino acids differentiating the leaf ranks. Values are the mean ± SD of 5 independent biological replicates. Different letters indicate groups of mean values that are significantly different between the different leaf ranks (ANOVA-Tukey HSD, *p*-value < 0.05). PCA analysis was performed on amino acids showing a normal distribution of their values (denoted in bold in the complete dataset available in Supplementary Table S1).

Next, we quantified amino acid contents in the different leaf ranks (complete dataset available in Supplementary Table S1). From this dataset, we performed a principal component analysis for amino acids showing a normal distribution of their values (Figure 2A,B). The two first major axes selected from the PCA (Dim1 and Dim2) explained up to 68% of the variations between the leaf ranks and succeeded to visually separate L15 leaves from other leaf ranks, along a “South-South-East North-North-West” axis (from Figure 2B). The loading plot (Figure 2A) showed the Pearson correlation coefficient for the variation in each amino acid with respect to the spatial separation of the leaf groups in the Dim1–Dim2 plane. We found that Asn, Gln, Pro and SMCSO showed strong positive Pearson correlation coefficients (0.89, 0.93, 0.93 and 0.85, respectively) along the leaf rank separation axis. Interestingly, Thr and Val showed negative Pearson correlation coefficients along a relatively similar axis (0.90 and 0.23, respectively; Figure 2A). Then, we performed statistical analysis for these amino acid contents between the different leaf groups (ANOVA followed by a post-hoc Tukey HSD test). Interestingly, Asn, SMCSO, Pro and Gln contents were significantly decreased in L11, L7 and L3 leaves compared to L15 leaves, while Thr content showed an opposite behavior (Figure 2C). Asn, SMCSO, Pro, Gln and Thr contents remained stable between the leaf ranks L11, L7 and L3, while some changes between L11-L7 and L3 could have been expected, due to their respective leaf status (source versus senescent). Val content was significantly higher only in L7 leaves compared with other leaf ranks, thus becoming a metabolic fingerprint specific to L7 leaves. Overall, we have identified four leaf ranks with a gradual increase in their source status and a specific metabolic fingerprint associated with amino acid metabolism. The specific variation in Asn, Gln, SMCSO, Pro, Thr and Val between leaves with contrasted sink/source balances suggested that their associated nitrogen metabolic fluxes might be affected by nitrogen remobilization processes.

### 2.2. Biosynthesis Fluxes of Pro, Val and Thr Are Reduced During the Sink/Source Transition of B. Napus Leaves

To assess modifications to nitrogen fluxes within the amino acid metabolism during nitrogen remobilization in winter oilseed rape, we conducted an instationary labeling experiment with [^15^N]_L_-glycine on leaf discs of the four leaf ranks with a different sink/source balance, with the idea of estimating metabolic fluxes using an INST-MFA approach. We chose [^15^N]_L_-glycine as a metabolic probe during short-term labeling experiments for two reasons. First, our recent use of ^15^NH_4_ to assess capacities for proline production in *B. napus* resulted in a non-physiological accumulation of Gln, which could also be harmful for the precise analysis of the ^15^N-labeling on other amino acids [29]. Second, a strong part of the activity of the chloroplastic Gln synthetase enzyme in illuminated plant leaves is devoted to the reassimilation of the NH_4_ lost by the activity of the photorespiratory enzyme Gly decarboxylase (GDC) [50]. Therefore, Gly appeared to be a relevant ^15^N-source to ensure a sufficiently high and rapid label incorporation through amino acid metabolism.

**Figure 3:**
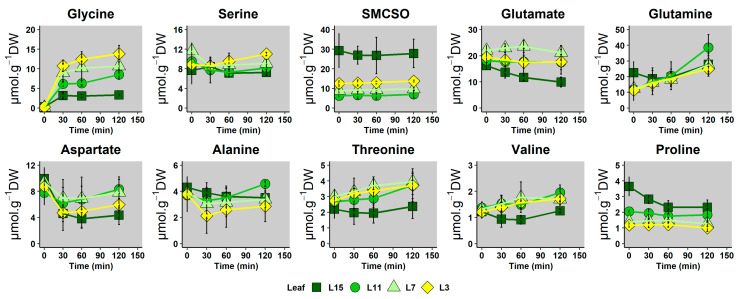
Time-course concentrations of the most abundant amino acids in leaf ranks with a different sink/source balance during an instationary labeling experiment with [^15^N]_L_-glycine as the sole nitrogen source. Leaf discs from the four leaf ranks having a different sink/source balance were floated during 0, 30, 60 or 120 min in a buffer containing 10 mM [^15^N]_L_-glycine, under continuous light and orbital shaking. Amino acid quantification was achieved with a UPLC-DAD system. Values are the mean ± SD of 3 independent biological replicates for each leaf group and each time-point. Complete dataset is available in Supplementary Table S2.

Following the transfer of leaf discs to the [^15^N]_L_-glycine media, the concentration and labeling dynamics of amino acids were measured at three time points (30, 60 and 120 min) with a UPLC-DAD system and a HPLC-HRMS system, respectively. The present analysis focused on amino acids found in major quantities (>1 µmoL g^−1^ DW in at least half of the samples). The complete dataset of all detected amino acids is available in Supplementary Material Tables S2 and S3. Metabolomics results (Figure 3) showed that the concentration of Gly rapidly increased from 0.2 to 4–12 µmoL g^−1^ DW following the isotopic switch and remained stable after 30 min. Surprisingly, the steady-state Gly concentration gradually increased in the leaf ranks following their sink/source status. In contrast, the concentration of other amino acids was not strongly impacted by the labeling experiment. Their pool size were slightly different between the different leaf ranks before the switch (metabolic fingerprint of senescence) and they showed weak variations during the entire experiment. These results indicated that metabolism rapidly reached a metabolic pseudo steady-state following the switch to ^15^N-Gly. Interestingly, the strength of the response for glycine content reflected the progressive sink/source transition of leaves.

**Figure 4:**
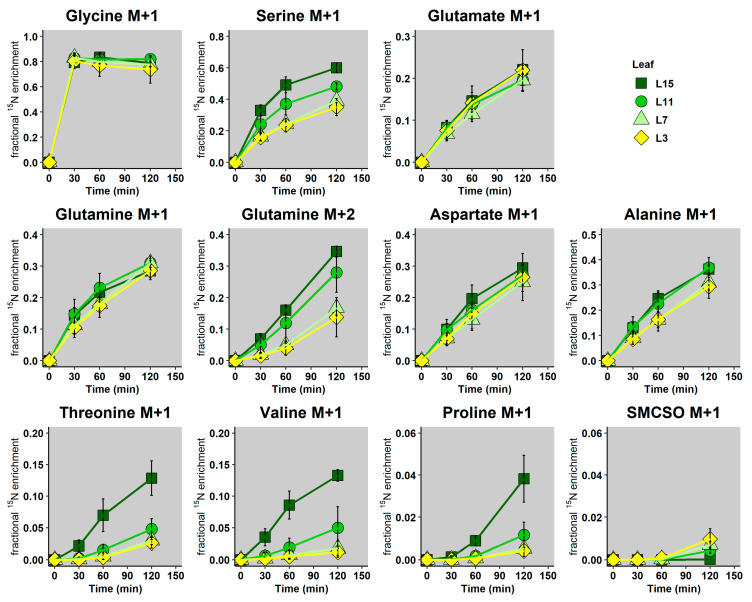
Time-course fractional ^15^N enrichment of the most abundant amino acids in leaf ranks having a different sink/source balance during an instationary labeling experiment with [^15^N]_L_-glycine as sole nitrogen source. Leaf discs from the four leaf ranks having a different sink/source balance were floated during 0, 30, 60 or 120 min in a buffer containing 10 mM [^15^N]_L_-glycine, under continuous light and orbital shaking. Analysis of nitrogen isotopologue distributions within amino acids was achieved with a HPLC-HRMS system. Values are the mean ± SD of 3 independent biological replicates for each leaf group and each kinetic. The complete dataset is available in Supplementary Table S3.

Isotopic analyses indicated that the fractional ^15^N-enrichment of Gly rapidly reached an isotopic steady-state (i.e., before 30 min), at around 80% for all leaf ranks, confirming that ^15^N-Gly had been infiltrated into leaf discs (Figure 4). This enrichment was similar for all leaves and was lower than expected (100%), which demonstrates the contribution of another (unlabeled) nitrogen pool to Gly, originating either directly from protein degradation or indirectly through transamination reactions. Interestingly, Ser, Val, Thr, Pro and Gln showed a slower rate of ^15^N incorporation between the different leaf ranks, following their sink/source status. Gln showed a surprising behavior, since only the Gln M+2 isotopologue (2 ^15^N per molecule) was impacted by the sink/source status of the leaf, whereas the fractional ^15^N-enrichment of Gln M+1 (1 ^15^N per molecule) increased relatively consistently for all leaves. Glu, Ala and Asp showed a similar evolution of their fractional ^15^N-enrichment over time between the different leaf ranks. Conversely, SMCSO only started to be weakly enriched after 120 min in all the leaf ranks (except L15), thus indicating that the biosynthesis of SMCSO from de novo assimilated nitrogen was too low with respect to the SMCSO pool size to be precisely detected in our experiment. From a global point of view, fractional ^15^N-enrichments of major amino acids reached relatively high values after 2 h (30% on average), with the exception of Pro and SMCSO, which reflects major differences in their biosynthesis and degradation dynamics compared with other amino acids.

**Figure 5:**
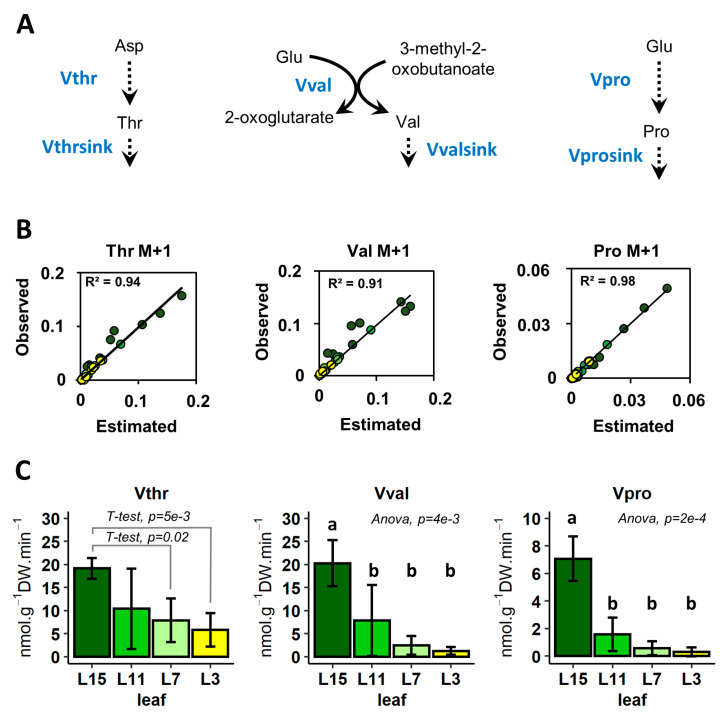
Estimation of metabolic fluxes for Thr, Val and Pro biosynthesis in leaf ranks with a different sink/source balance by using a ^15^N-INST-MFA approach. (**A**) Metabolic subsystems considered for the estimation of metabolic fluxes; (**B**) Comparison of estimated (flux-based) and observed fractional ^15^N enrichment for the considered amino acids; (**C**) Pro biosynthesis. Local estimation of metabolic fluxes (in nmoL.g^−1^ DW.min^−1^) was achieved using the ScalaFlux approach with the IsoSim v2software by minimizing the sum of squared weighted errors between the observed and simulated experimental data (amino acid concentrations and label propagations). Values are the mean ± SD of 3 independent biological replicates for each leaf group. Different letters indicate groups of mean values that are significantly different between the different leaf ranks (ANOVA-Tukey HSD, *p*-value < 0.05). For Vthr (ANOVA *p*-value = 0.068), additional student tests were performed between L15-L7 and L15-L3 (*p*-values shown within the graph). Complete dataset is available in Supplementary Tables S4 and S5.

Since metabolomics results indicated that all leaves were at metabolic pseudo steady-state during ^15^N-label propagation, we decided to apply an ^15^N-INST-MFA approach to estimate metabolic fluxes in the different leaves. Since Gly consumed during the experiment is below the precision of Gly quantification (which is around 2–5%), the glycine uptake could not be quantified. Using classical MFA approaches, the lack of identifiability of the glycine uptake flux would propagate downstream, thereby affecting the rest of the network for which fluxes could not be calculated. We thus applied an alternative approach, ScalaFlux [48], which can quantify fluxes through any metabolic subnetwork of interest by modeling label propagation directly from the metabolic precursor of this subnetwork. The flux calculations are thus purely based on information from within the subnetwork of interest, and no additional knowledge about the surrounding network (such as label propagation through upstream reactions or the labeling of the extracellular nutrient) is required. Based on the accessible isotopic data and on the Plant Metabolics Network (PMN) database (www.plantcyc.org), we identified three independent subsystems for which fluxes can be calculated: The biosynthesis of Val and Pro from Glu, and the biosynthesis of Thr from Asp. Following the ScalaFlux workflow [48], we constructed a flux model for each of these subsystems (Figure 5A). All flux models contain only two reactions: A biosynthetic reaction and a sink reaction that operate at the same rate to reflect the observed metabolic pseudo steady-state. Experimental labelling dynamics of the precursors of these subnetworks (Glu and Asp) were used as label input, and metabolic fluxes were estimated by the fitting concentrations and labelling dynamics of Thr, Val and Pro (See Material and Methods and Figure 5B). The results showed that the biosynthesis fluxes in Val, Thr and Pro were statistically decreased in the source leaves, L7 and L3, compared to sink leaves (L15), and this response was similar for the three amino acids (Figure 5C). In L15 leaves, Thr and Val biosynthesis fluxes were three times higher than the Pro biosynthesis flux, and this ratio seemed to be relatively conserved between the different leaves. The decrease in Pro content during the acquisition of the source status by leaves appears to be explained by the decrease in the Pro biosynthesis flux, whereas Thr and Val pools tend to increase despite the apparent reduction in their biosynthesis fluxes. Overall, these results highlighted a progressive and gradual reorganization of amino acid metabolism of leaves during sink/source transition at the vegetative stage and suggests a strong reduction in de novo amino acid biosynthesis.

## 3. Discussion

Source–sink relationships are the major support for nitrogen remobilization/recycling processes in crop plants, influencing yield elaboration and nutritional quality of harvested products, especially seeds [11,51]. These nitrogen remobilization processes are spatially separated between source leaves (for protein degradation and production or recycling of amino acids/peptides), phloem tissues (for transport of amino acids/peptides) and sink leaves (for metabolic use of amino acids/peptides). The acquisition of source status by leaves generally occurs during ageing and leads to major modifications to transcript levels, protein levels and metabolite levels [21,22,30]. These changes reflect modifications to metabolic fluxes within the primary metabolism network that still remain to be described in order to ultimately identify the determinant metabolic steps of N recycling. Indeed, the direct prediction of metabolic fluxes from metabolic fingerprints can be very difficult, since modifications to metabolic fluxes are not always associated with changes in the size of metabolite pools [52], as shown in this study.

In this work, we characterized major changes occurring in amino acid metabolism during nitrogen remobilization in leaves, with a gradual increase in their source/senescent status by combining metabolic profiling with a fluxomic approach. We clearly identified a specific metabolic fingerprint in the amino acid metabolism that was associated with the acquisition of source status by leaves. Indeed, contents of Asn, SMCSO, Gln, Pro and Thr were significantly decreased in source and senescent leaves (L11, L7 and L3 leaves) whereas Val content was significantly increased in L7 leaf (Figure 2). In many plant species, developmental leaf senescence and inherent nitrogen remobilization processes are associated with a depletion of amino acid contents. Developmental leaf senescence correlated with the decrease in Ser, Gly, Glu, Ala, Asp, Pro and Val contents in *Arabidopsis* [21] and in tobacco [53]. A recent analysis of the metabolic fingerprint associated with leaves having a different sink/source status in winter oilseed rape genotype *Darmor bzh* showed a correlation between the decrease in Glu and Asp contents in the acquisition of source status by leaves [54]. Since we performed our analysis on a winter oilseed rape genotype *Aviso*, our results suggested that metabolic fingerprint could be an interesting tool to separate different genotypes. Nevertheless, the depletion of Gln and Asn contents is often associated with their remobilization and probably their translocation through the phloem [55]. Based on our analysis, we may suggest that SMCSO and Pro are also remobilized directly through the phloem. Such a conclusion is partly supported by our recent work, which showed that Pro catabolism played a minor role during nitrogen remobilization processes in source leaves of oilseed rape [29]. In addition, our fluxomic approach using a ^15^N tracer clearly demonstrated that Pro biosynthesis flux was strongly decreased in source leaves. (Figure 5). Finally, under drought stress, phloem exudates from different leaf ranks of oilseed rape can be strongly enriched in Pro, suggesting that Pro was efficiently remobilized during drought stress [56]. On the other hand, the question remains unsolved for SMCSO, an amino acid whose synthesis and degradation pathways are not yet fully known. To date, SMC, the precursor of SMCSO, is synthesized from Met and Ser [57]. Interestingly, the N atom present in the SMC can be specifically inherited from Ser, through O-acetyl-serine biosynthesis. In our ^15^N-labeling experiment, fractional ^15^N enrichment of SMCSO was very low in all the leaf ranks after 2 h and undetectable for SMC, whereas fractional ^15^N enrichment of Ser was strongly increased during the experiment (Figure 3). Therefore, the low ^15^N incorporation into SMCSO in leaves of winter oilseed rape after 2 h of labeling can be explained by a low de novo biosynthesis of SMCSO from Ser, the use of a non-labeled O-acetyl-serine pool for the de novo biosynthesis of SMCSO, a strong isotopic dilution, due to the large pool size of SMCSO (major free soluble amino acid of leaves).

^15^N-INST-MFA showed that N assimilation into Pro, Val and Thr was progressively decreased during the acquisition of source status by leaves. Such results contrasted with intuitive interpretations based on the overall variation in Val and Thr pools between the different leaf ranks, thus emphasizing the necessity of gaining access to metabolic fluxes to fully understand the metabolic reconfiguration orchestrated at the cellular level during the evolution of the sink/source balance. Nevertheless, the decrease in Pro, Val and Thr biosynthesis fluxes could also reflect an overall decrease in protein biosynthesis in addition to the increased protein remobilization (Figure 1). In fact, a dynamic ^13^CO_2_ labeling experiment in *Arabidopsis* leaves showed that soluble free and protein-bound amino acids were both rapidly enriched in freshly assimilated ^13^C after 4 h [58]. Another study on barley leaves using ^15^NO_3_ demonstrated that proteins could be enriched in ^15^N up to 20% after 12 h of light [59]. Both results confirmed that proteins were the major sink for amino acids in illuminated leaves. In our experimental ^15^N-INST-MFA setup, where each amino acid appeared to be at metabolic pseudo steady-state, the reduction of amino acid biosynthesis could reflect a reduction in amino acid requirements towards protein synthesis, thereby suggesting that de novo protein biosynthesis was also decreased in source leaves. Consequently, we propose that protein turn-over during nitrogen remobilization processes in winter oilseed rape is regulated in two ways: by decreasing protein synthesis and by activating protein degradation. Interestingly, the first step of the Val degradation pathway is achieved by BCAA transaminase (BCAT) enzymes, which are exactly the same enzymes as used for Val biosynthesis [19]. Therefore, our estimation of Val biosynthesis flux in our ^15^N labeling experiment can also directly reflect the fraction of active BCAT, thereby suggesting that the decrease in BCAT activity in source leaves compared to sink leaves (L15) could similarly affect Val biosynthesis and its degradation. In *Arabidopsis*, the degradation of BCAA can be triggered by dark-induced senescence and provides a source of alternative substrates for the mitochondrial respiration [24]. Indeed, *Arabidopsis* mutants for an isovaleryl-CoA dehydrogenase and two electron-transfer flavoprotein:ubiquinone oxidoreductases (enzymes downstream of the BCAT producing or processing electrons to the mitochondrial electron transfer chain) accumulated 3–5 times more Val compared to the control after 7–15 days of dark-induced senescence. Given the close link between natural and dark-induced senescence, an increase in the BCAT activity could have been expected in our experiments, at least for L3 leaves (ongoing natural senescence). However, BCAT activity may not be rate-limiting for the overall Val degradation during senescence. In fact, the transcript levels of genes encoding mitochondrial BCATs remained relatively stable during natural senescence, while the transcript levels of genes encoding isovaleryl-CoA dehydrogenases strongly accumulated in *Arabidopsis* [21]. In addition, the specific regulation of Val degradation may differ between natural and dark-induced senescence. Indeed, our recent work in winter oilseed rape showed that the mitochondrial Pro catabolism was preferentially activated by dark-induced senescence rather than the acquisition of the source status [29]. Similarly, Val degradation must be activated during natural senescence, but may act at a later stage, i.e., when other major carbon sources have already been remobilized.

To summarize, we have identified a specific metabolic fingerprint within the amino acid metabolism that is associated with the acquisition of the source status in leaves of winter oilseed rape. We found that Val, Thr and Pro biosynthesis fluxes were progressively decreased in source leaves compared to young leaves, thereby suggesting an overall decrease in amino acid biosynthesis during nitrogen remobilization processes. Consequently, nitrogen remobilization from protein pool may be partly activated by decreasing de novo protein biosynthesis, in addition to the activation of protein degradation. Future work will focus on developing more detailed and complete isotopic models of the compartmented nitrogen metabolism of winter oilseed rape. This effort will be necessary to provide a comprehensive understanding of the dynamic reorganization of amino acid metabolism, and more generally of nitrogen metabolism (such as fluxes through transamination reactions), during sink/source transition in leaves.

## 4. Materials and Methods

### 4.1. Plant Material and Growth Conditions

Seeds of the *B. napus* genotype Aviso (“Bracysol” Biological Resource Center (IGEPP)) were first incubated during 3 d on a soaked blotting paper to allow seedling establishment. Then, seedlings were individually transferred in 4 L pots filled with a non-fertilized commercial substrate (Falienor, Vivy, France, reference 922016F3). Plants were grown in a 6 m^3^ growth chamber with the following parameters: a (14 h-22 °C) light/(10 h-16 °C) dark cycle, ambient air with 65% to 80% of relative humidity, 100 µmoL photons m^−2^ s^−1^ of a photosynthetically active radiation at the top of the canopy. Plants were watered twice a week with a commercial fertilized solution (Liquoplant Bleu, 2.5% N, 5% P, 2.5% K). Experiments were performed on plants grown for 60 days after sowing. These plants possessed 16 leaf ranks with the two oldest yellowing leaves already fallen off, confirming that nutrient remobilization processes between leaves were operating. Leaf ranks were annotated from the bottom to the top (L3 to L16). To simplify our analysis, we worked with four leaf ranks (L15, L11, L7 and L3) selected on the basis of their order of appearance in the plant (young, pre-mature and post-mature and old leaves) and their visual color.

### 4.2. Quantification of Protein, Chlorophyll, Carbon and Nitrogen Content

Chlorophyll contents were measured in SPAD units on a leaf area basis using the non-destructive chlorophyll SPAD-502 meter (Konika Minolta, Europe) and were expressed relatively to the L15 leaf rank. For protein, carbon and nitrogen content quantification, leaves were instantly immersed in liquid nitrogen. Samples were freeze-dried for 72 h and then ground to a fine powder. Soluble proteins were extracted from 10 mg of freeze-dried powder with 1 mL of a citrate-phosphate buffer containing 20 mM Citrate, 160 mM Na_2_HPO_4_ (pH 6.8) supplemented with a spoon of polyvinylpyrrolidone and a cocktail of antiprotease inhibitor (cOmplete™, EDTA-free Protease Inhibitor Cocktail, Merck, Darmstadt, Germany). After a 15 min incubation step with an orbital shaking of 1500 rpm, samples were centrifuged for 10 min at 4 °C at 12,000× *g*. The supernatant was recovered and proteins were quantified using the Bradford reagent against a standard curve with BSA as the standard. Carbon and Nitrogen contents were determined by Dumas combustion method with an automated CN analyzer (Elementar Vario Micro cube CHNS; Elementar Analysensysteme GmbH, Langenselbold, Germany) from 5 mg of freeze-dried samples, previously dried overnight at 80 °C.

### 4.3. Time-Course Experiment with [^15^N]_L_-glycine

One hour after the beginning of the light period, each leaf was cut at the basis of the petiole and then 24 leaf discs (0.8 cm^2^) were randomly punched with a cork-borer in both laminas of each selected leaf. Leaf discs were first floated in a 10 mM MES-KOH (pH 6.5) buffer for 15 min in order to process the four leaves per plant. Then for each time point, six leaf discs from the same leaf were transferred in a well of a 6-well microplates filled with 4 mL/well of a buffer containing 10 mM MES-KOH (pH 6.5) and 10 mM [^15^N]_L_-glycine (^15^N isotopic purity of 98%) (Merck, Darmstadt, Germany). All incubations were performed at 20 °C under a continuous light of 100 µmol photons m^−2^ s^−1^ and an orbital shaking of 70 rpm. For each harvested time point (30, 60 or 120 min of incubation), leaf discs were rinsed three times (10 sec) with a neutral buffer (i.e., without ^15^N-glycine) and dried for 30 s on a clean tissue before being frozen in liquid nitrogen and stored at −80 °C.

### 4.4. Quantification of Amino Acid Content and Fractional ^15^N-enrichment

Prior to metabolite extraction, samples harvested in liquid nitrogen were freeze-dried for 72 h. For polar metabolite extraction, around 10 mg of dry weight was incubated for 15 min under an orbital shaking of 1000 rpm at room temperature with 400 µL of cold MeOH (−20 °C) containing 200 µM of DL-3-aminobutyric acid (used as the standard for amino acid quantification). Then, 200 µL of CHCl_3_ were added and the samples were incubated again for 15 min with an orbital shaking of 1000 rpm. Finally, 400 µL of H_2_O were added to the samples and the biphase was obtained after a centrifugation step of 5 min at room temperature at 12,000× *g*. The upper polar phase (MeOH/H_2_O fraction) was recovered and 200 µL were evaporated using a SpeedVac concentrator at 35 °C for 2 h.

For amino acid quantification by UPLC-UV, amino acids were derivatized using AccQTag derivatization kit (Waters, Milford, MA, USA) following manufacturer’s procedures and the method previously described [29,60]. For each batch of sample analysed, a few injections of a blank sample (an extraction performed without plant powder) and a commercial mix of amino acid standards were randomly performed between the samples. Peaks were integrated using Empower software (Waters) and were visually inspected. Absolute quantification of amino acids was performed using the UV-detection of amino acids (DAD detector), the internal standard, external calibration curves for each amino acid (commercial mix of amino acid standards) and blank correction.

For the determination of nitrogen isotopologue distributions by LC-HRMS, amino acids were separated on a PFP column (150 × 2.1 mm i.d., particle size 5 µm; Supelco Inc, Bellefonte, PA, USA) based on a method previously described [61]. The volume of injection was 5 µL. High-resolution experiments were performed with a Vanquish HPLC system coupled to an Orbitrap Qexactive+ mass spectrometer (Thermo Fisher Scientific, Waltham, MA, USA) equipped with a heated electrospray ionization probe. MS analyses were performed in positive FTMS mode at a resolution of 70,000 (at 400 *m/z*) in full-scan mode, with the following source parameters: capillary temperature at 320 °C, source heater temperature at 300 °C, sheath gas flow rate at 40 a.u. (arbitrary units), auxiliary gas flow rate at 10 a.u., S-Lens RF level at 40%, and source voltage at 5 kV. Metabolites were determined by extracting the exact mass with a tolerance of 10 ppm. Nitrogen isotopologue distributions were obtained after correcting the raw isotopic data (mass fractions) for the contribution of naturally occurring isotopes of all elements and for the isotopic purity of the substrate (98%), using the IsoCor v2.1.4 package of Python [62] (https://github.com/MetaSys-LISBP/IsoCor).

### 4.5. Local Estimation of Metabolic Fluxes Using ScalaFlux, a Scalable ^15^N-INST-MFA Approach

Flux calculations were carried out using the ScalaFlux approach implemented in the IsoSim v2.0.0 software (https://github.com/MetaSys-LISBP/IsoSim), as detailed in [48]. First, three metabolic subsystems were defined to represent Thr, Val and Pro biosynthesis (Figure 5A). Fluxes were then estimated using a two-steps approach by (i) defining analytical functions of the local label input and (ii) fitting experimental concentration and labeling dynamics of the metabolic intermediate, as detailed in the ScalaFlux publication. The objective function (defined as the sum of squared weighted differences between measured and simulated data) was minimized using the *nlsic* optimization algorithm [63]. For each biological replicate, the goodness-of-fit was verified using a chi-square test [48,64], assuming a precision on the isotopic measurements of 0.02 [61]. The code used to calculate fluxes for each subsystem and biological replicate is provided in Supplementary Code S1 to ensure reproducibility and reusability.

### 4.6. Statistical Analysis

Results are expressed as the mean ± standard deviation (SD) of n independent biological replicates (*n* = 3–5). The means of different leaf ranks were compared together with one-way ANOVA followed by a post-hoc Tukey’s HSD test for multiple pairwise comparison, or by a Student test (*p*-value < 0.05 for each test), by using R base [65]. Principal component analysis was achieved with the R package FactoMineR [66].

## Supplementary Materials

The following are available online at https://www.mdpi.com/2218-1989/10/4/150/s1, Table S1: Complete dataset of free amino acid contents from the four leaf ranks having a different sink/source balance, Table S2: Complete dataset of fractional ^15^N enrichment of major amino acids during a kinetic of labeling with [^15^N]_L_-Glycine on leaf ranks having a different sink/source balance, Table S3: Complete dataset of content of major amino acids during a kinetic of labeling with [^15^N]_L_-Glycine on leaf ranks having a different sink/source balance, Table S4: Complete dataset of observed and estimated fractional ^15^N enrichment of Pro, Val and Thr, Table S5: Complete dataset of estimated metabolic fluxes, Code S1. R scripts used to calculate fluxes in each leaf and each plant from amino acid concentrations and labeling dynamics.
