# Supplementary figures and images for: Sink/Source Balance of Leaves Influences Amino Acid Pools and Their Associated Metabolic Fluxes in Winter Oilseed Rape (Brassica napus L.)
Source: Metabolites. 2020 Apr 13;10(4):150. doi: 10.3390/metabo10040150 (PMC7240945; doi:10.3390/metabo10040150)

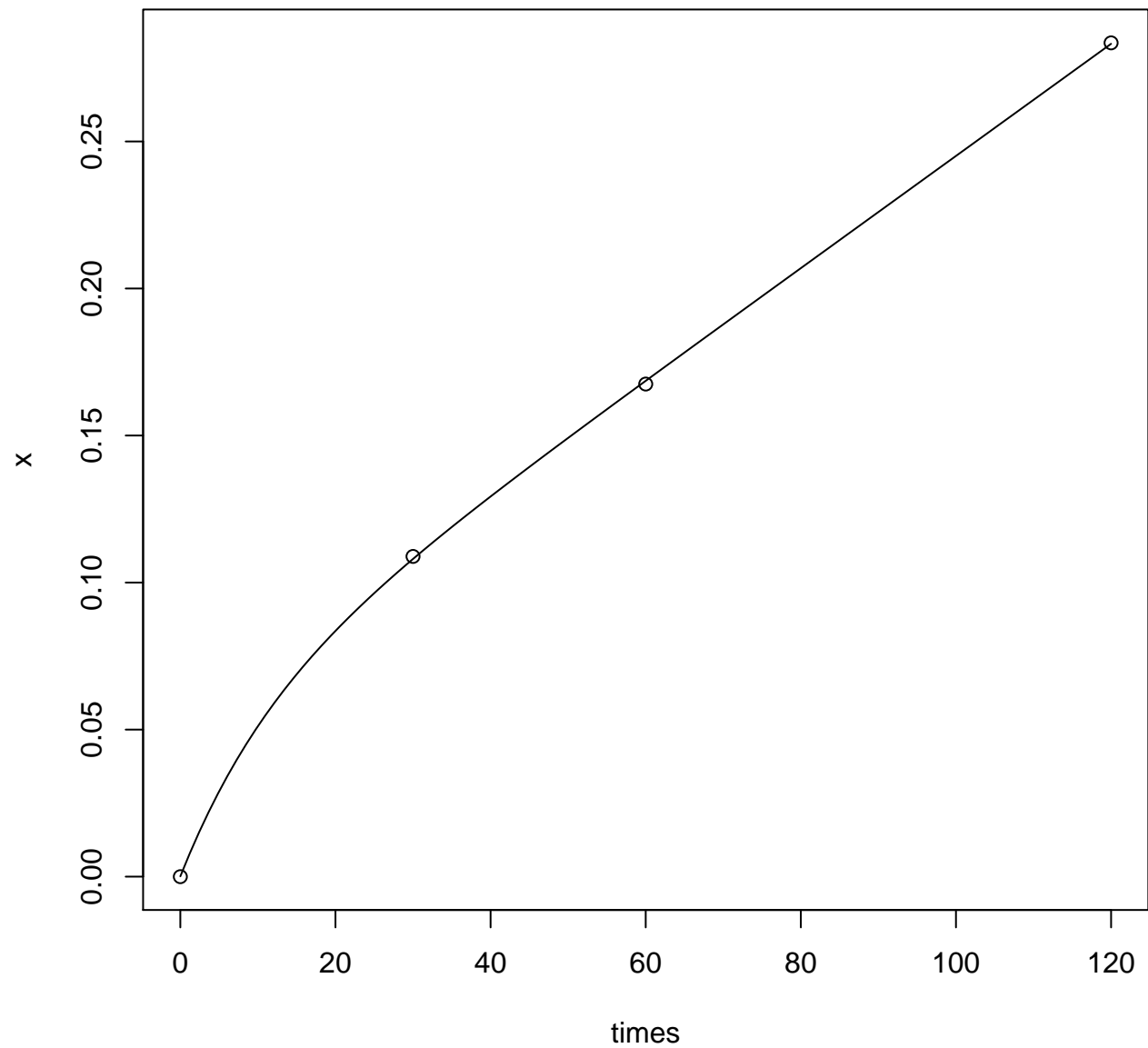

Supplement: Supplementary file 1 [file metabolites-10-00150-s001.zip › Supplementary Code S1/Asp-Thr/AL11/fit_input/res_fit_enr_AL11_asp_1.pdf]

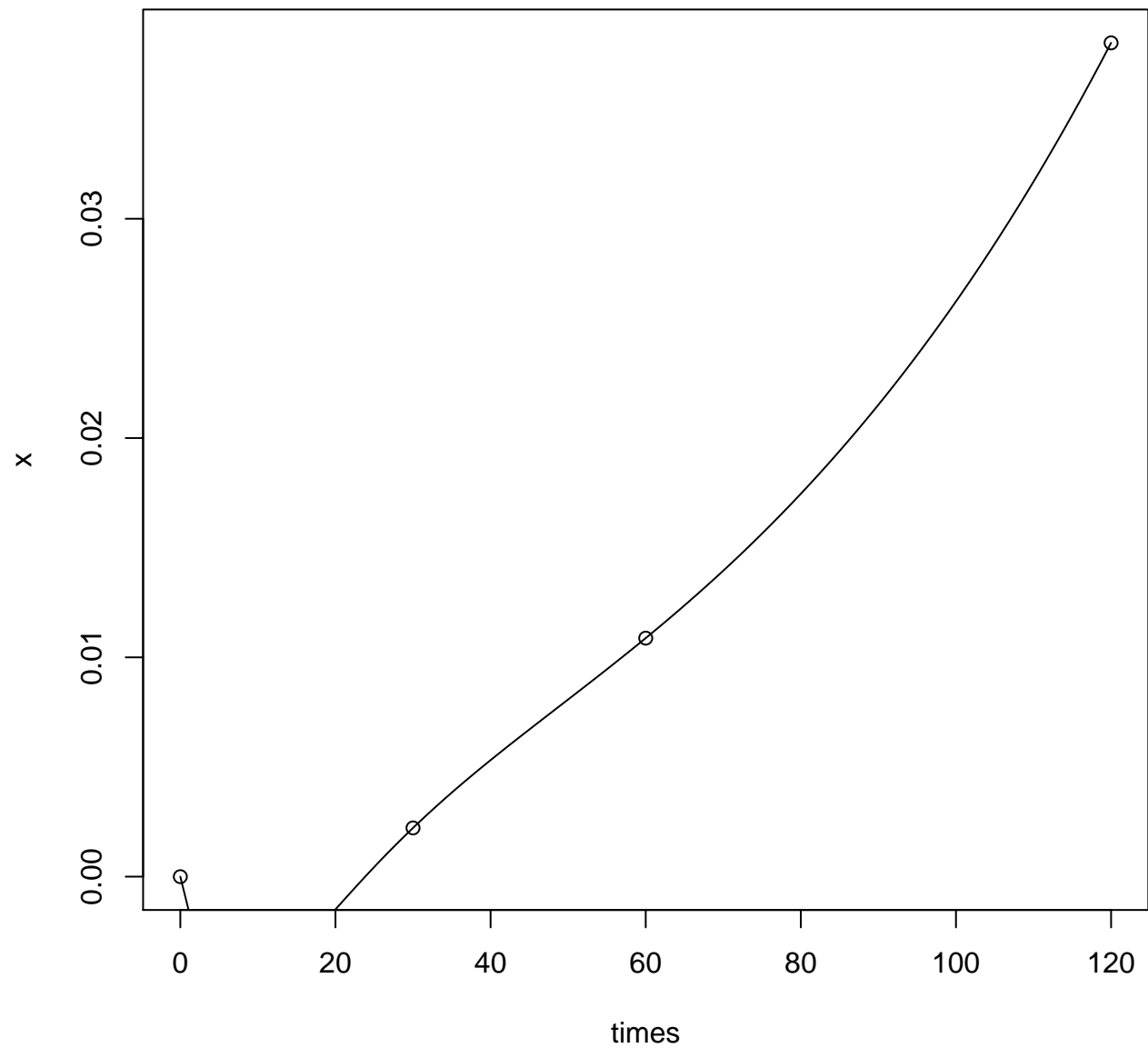

Supplement: Supplementary file 1 [file metabolites-10-00150-s001.zip › Supplementary Code S1/Asp-Thr/AL11/fit_input/res_fit_enr_AL11_thr_1.pdf]

## fitting results

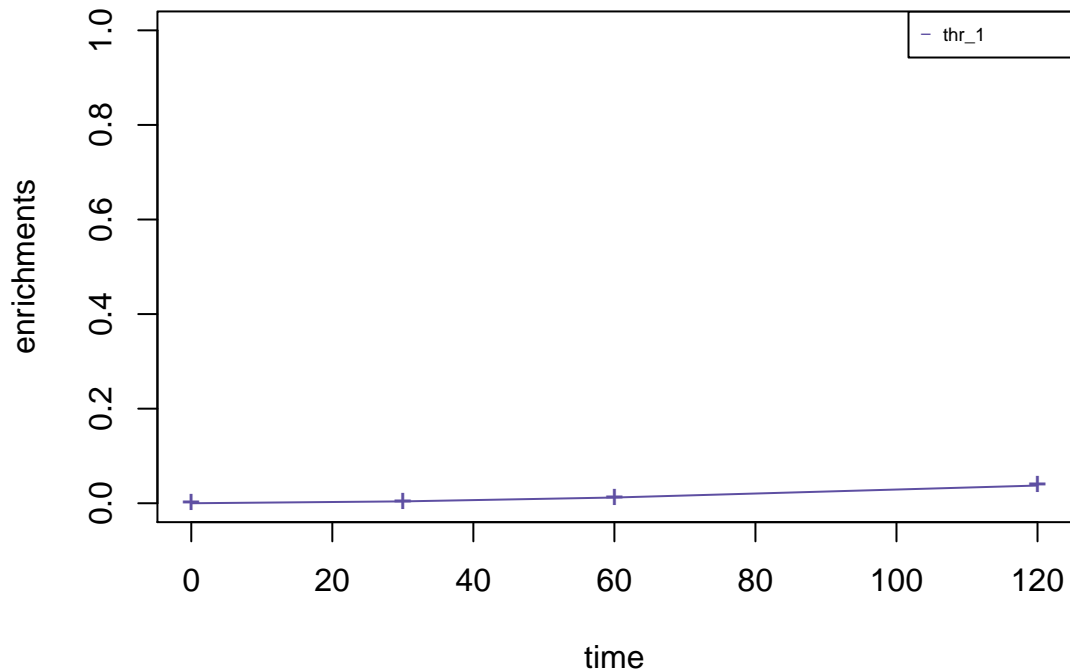

Supplement: Supplementary file 1 [file metabolites-10-00150-s001.zip › Supplementary Code S1/Asp-Thr/AL11/fit_subnet_fitthr/results.pdf]

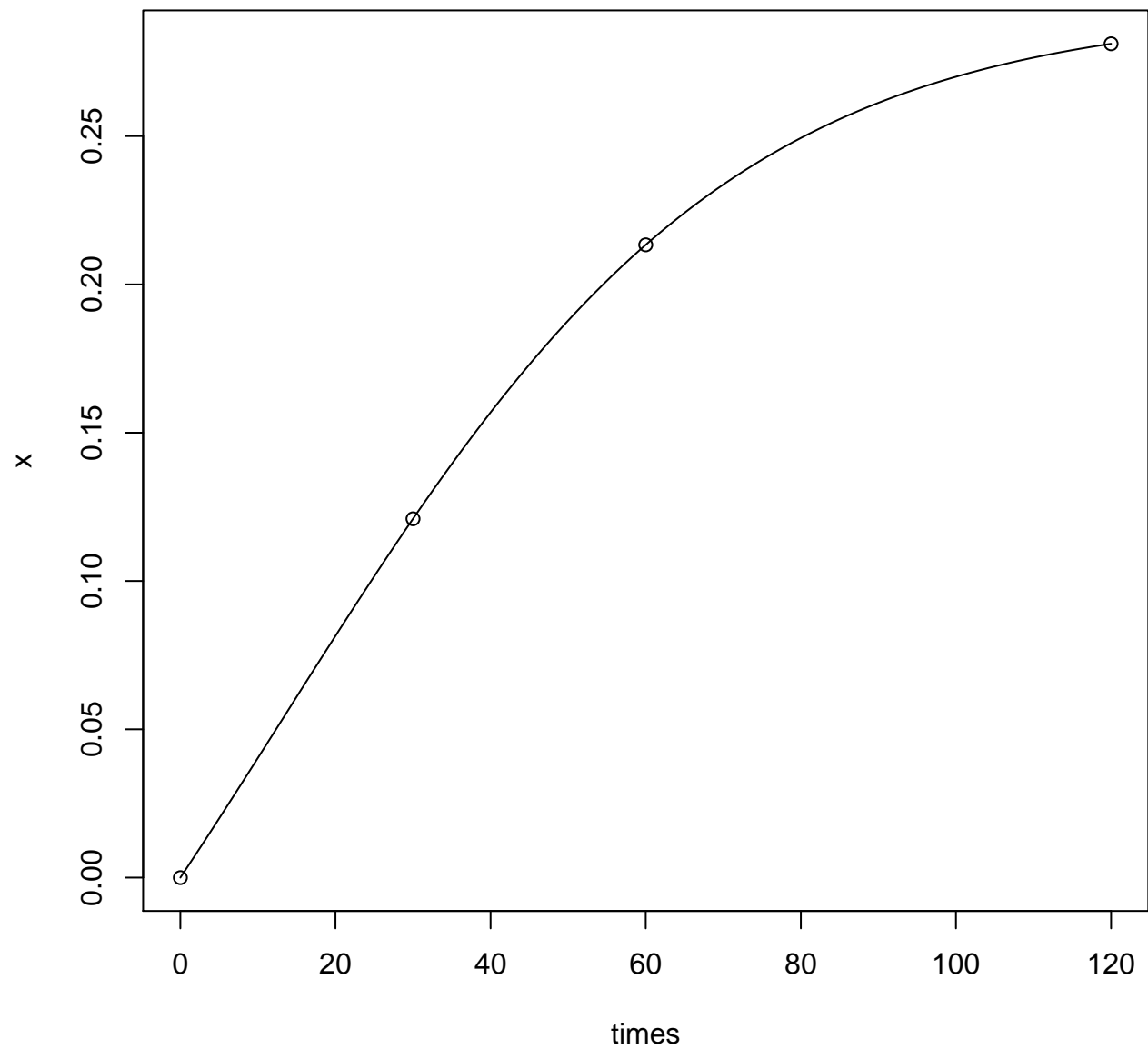

Supplement: Supplementary file 1 [file metabolites-10-00150-s001.zip › Supplementary Code S1/Asp-Thr/AL15/fit_input/res_fit_enr_AL15_asp_1.pdf]

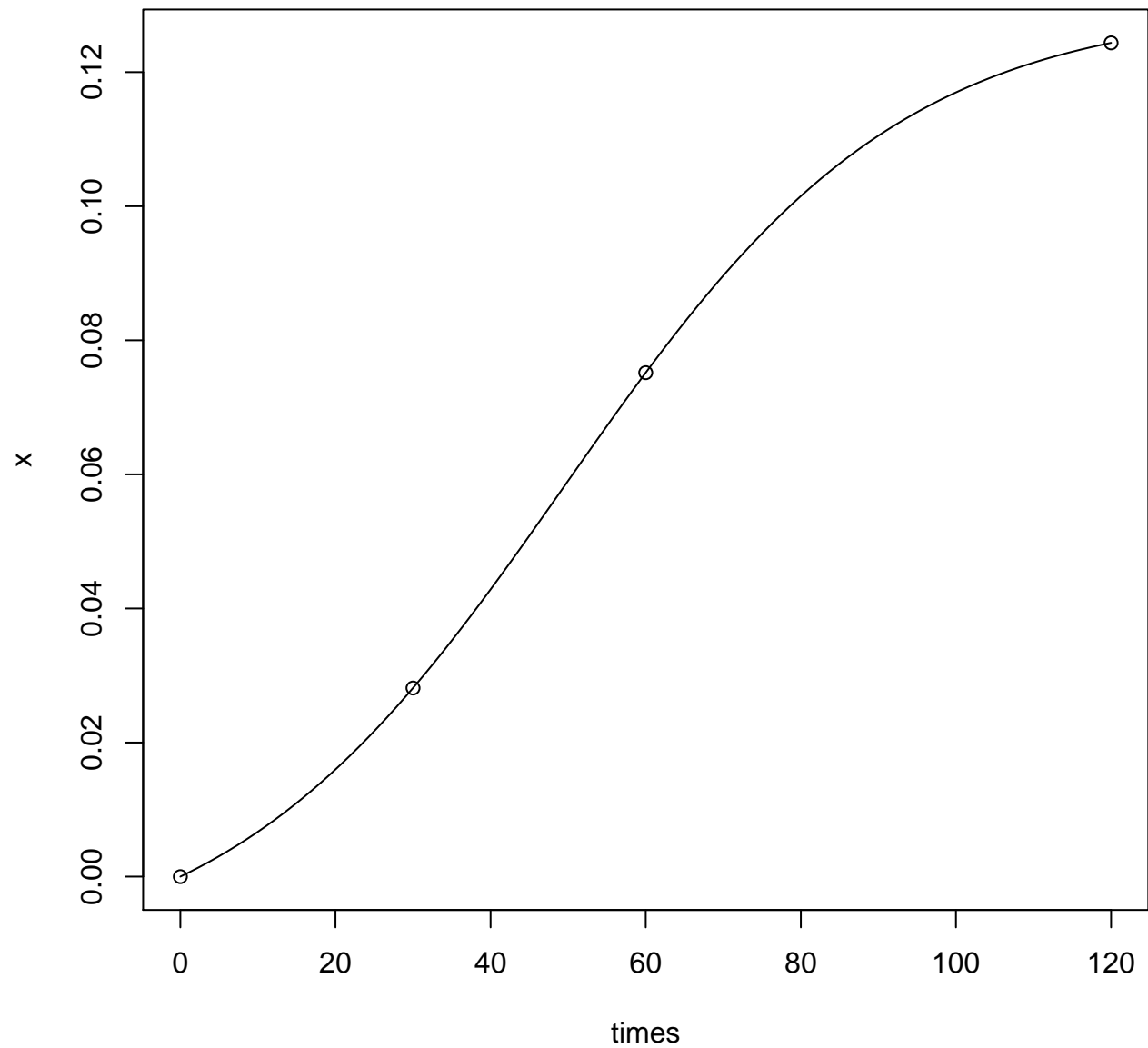

Supplement: Supplementary file 1 [file metabolites-10-00150-s001.zip › Supplementary Code S1/Asp-Thr/AL15/fit_input/res_fit_enr_AL15_thr_1.pdf]

## fitting results

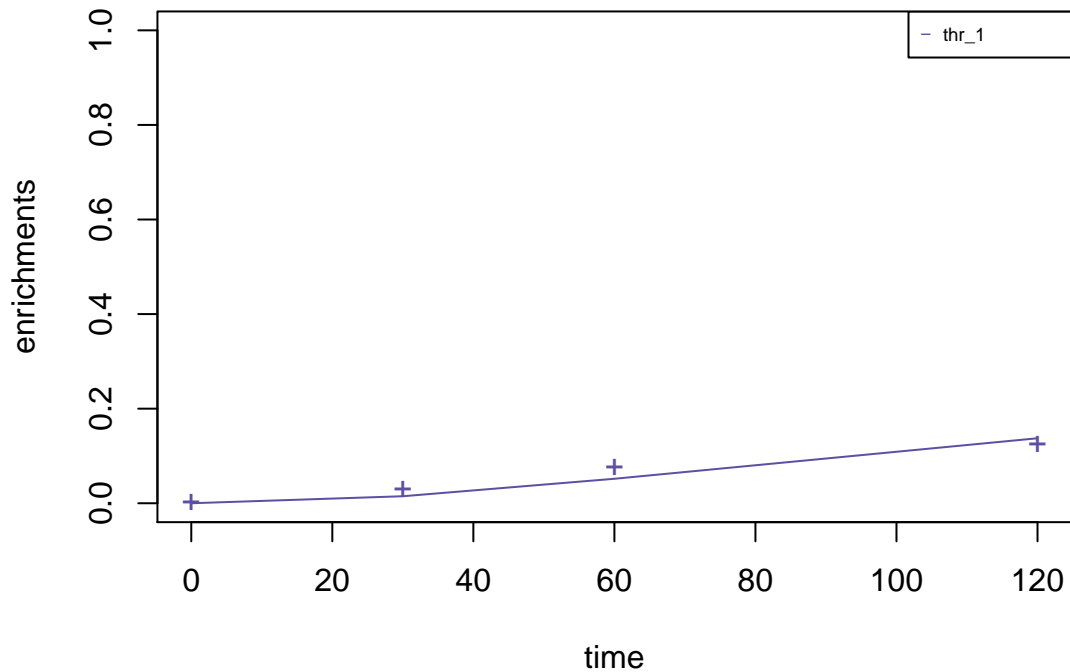

Supplement: Supplementary file 1 [file metabolites-10-00150-s001.zip › Supplementary Code S1/Asp-Thr/AL15/fit_subnet_fitthr/results.pdf]

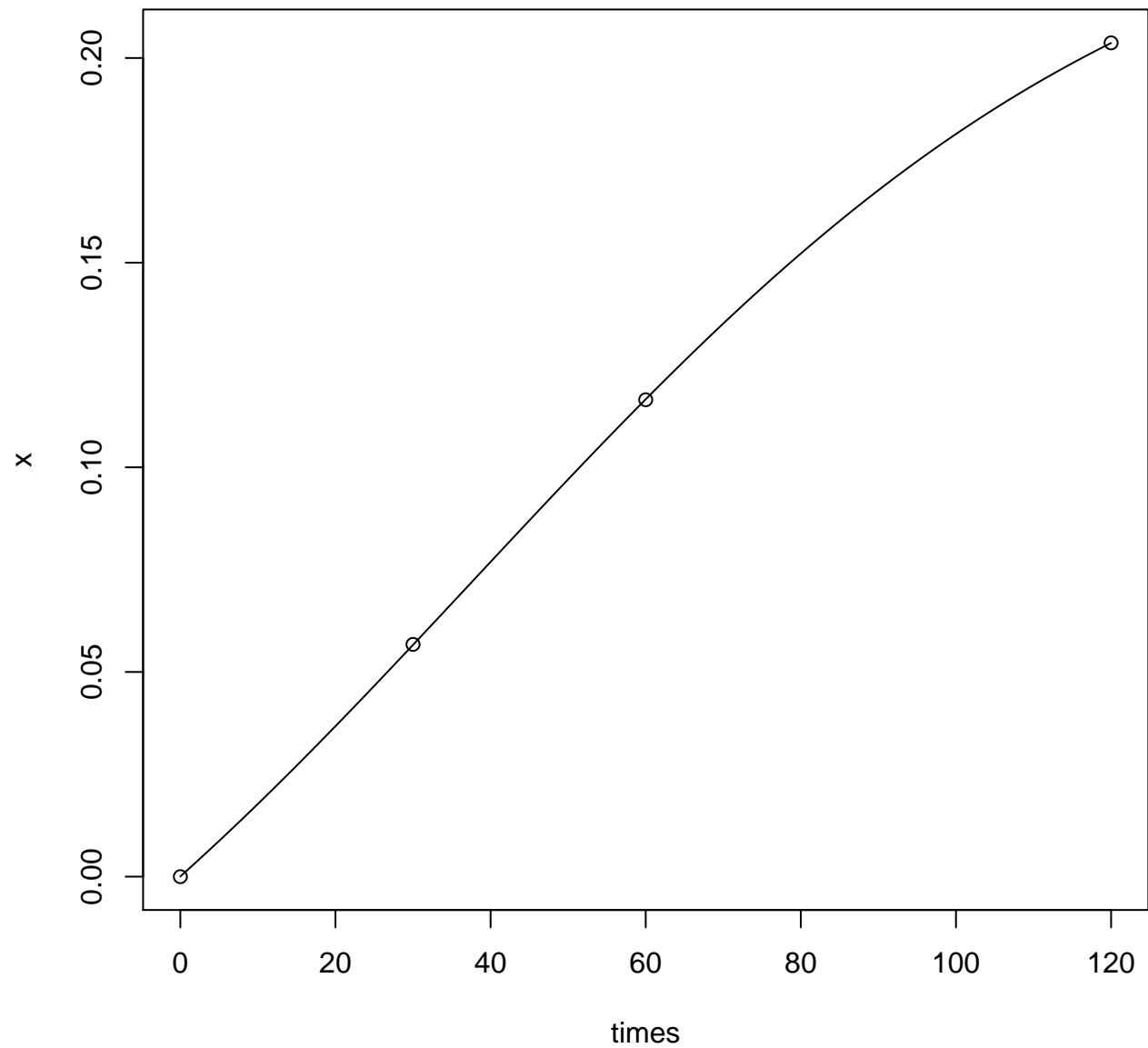

Supplement: Supplementary file 1 [file metabolites-10-00150-s001.zip › Supplementary Code S1/Asp-Thr/AL3/fit_input/res_fit_enr_AL3_asp_1.pdf]

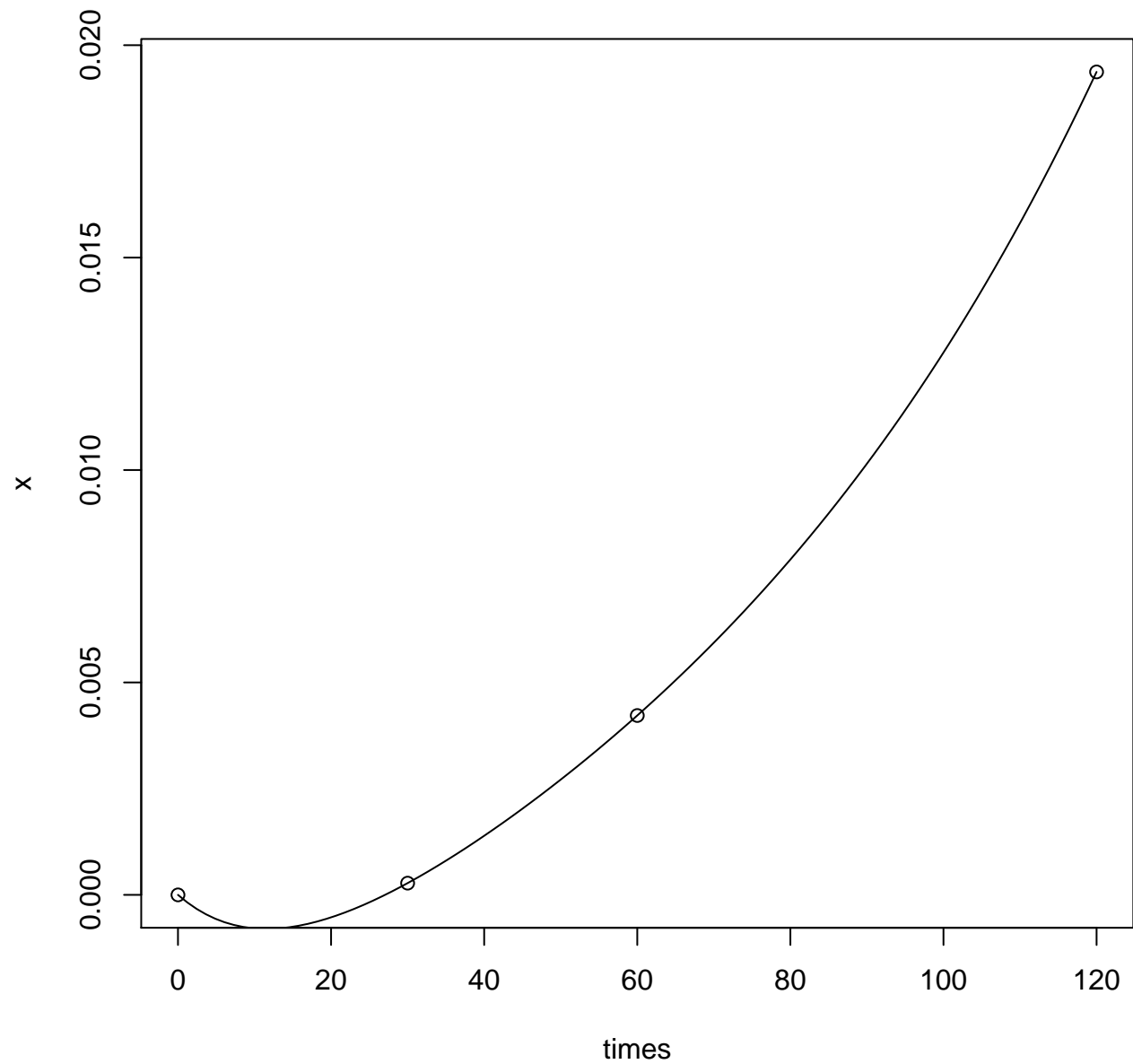

Supplement: Supplementary file 1 [file metabolites-10-00150-s001.zip › Supplementary Code S1/Asp-Thr/AL3/fit_input/res_fit_enr_AL3_thr_1.pdf]

## fitting results

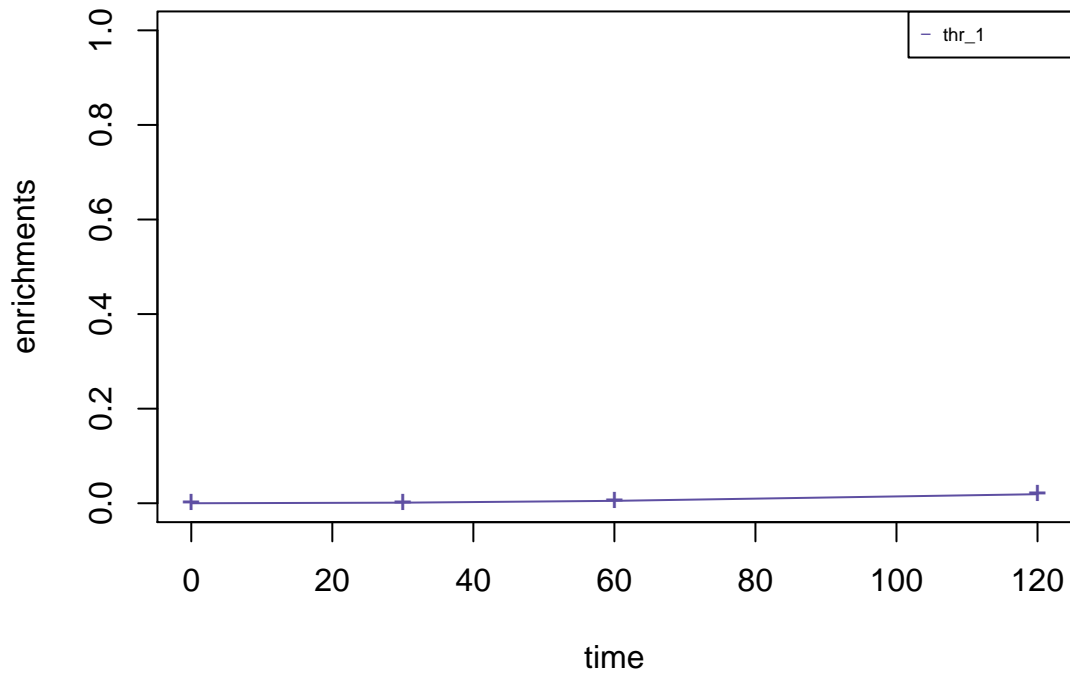

Supplement: Supplementary file 1 [file metabolites-10-00150-s001.zip › Supplementary Code S1/Asp-Thr/AL3/fit_subnet_fitthr/results.pdf]

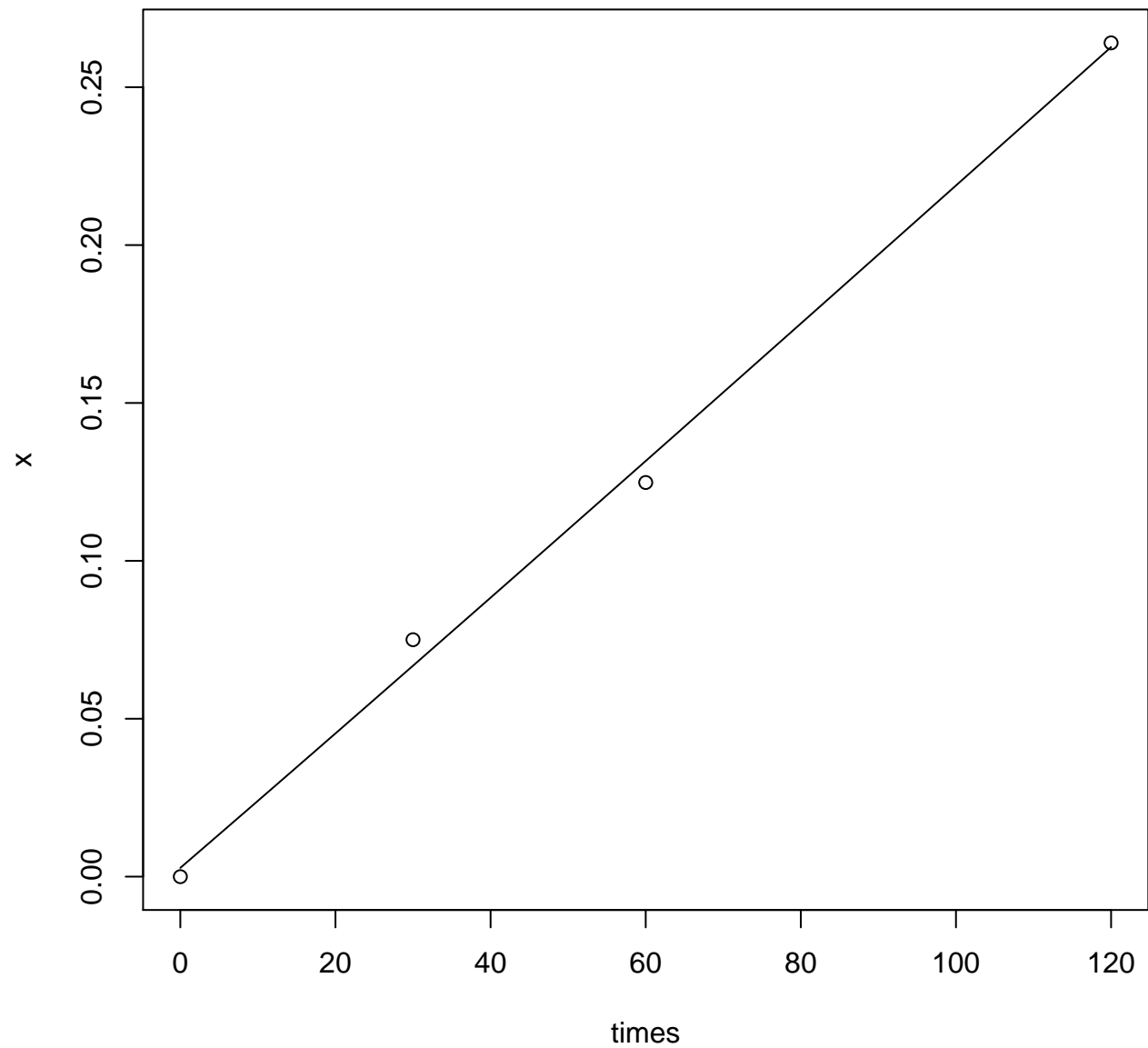

Supplement: Supplementary file 1 [file metabolites-10-00150-s001.zip › Supplementary Code S1/Asp-Thr/AL7/fit_input/res_fit_enr_AL7_asp_1.pdf]

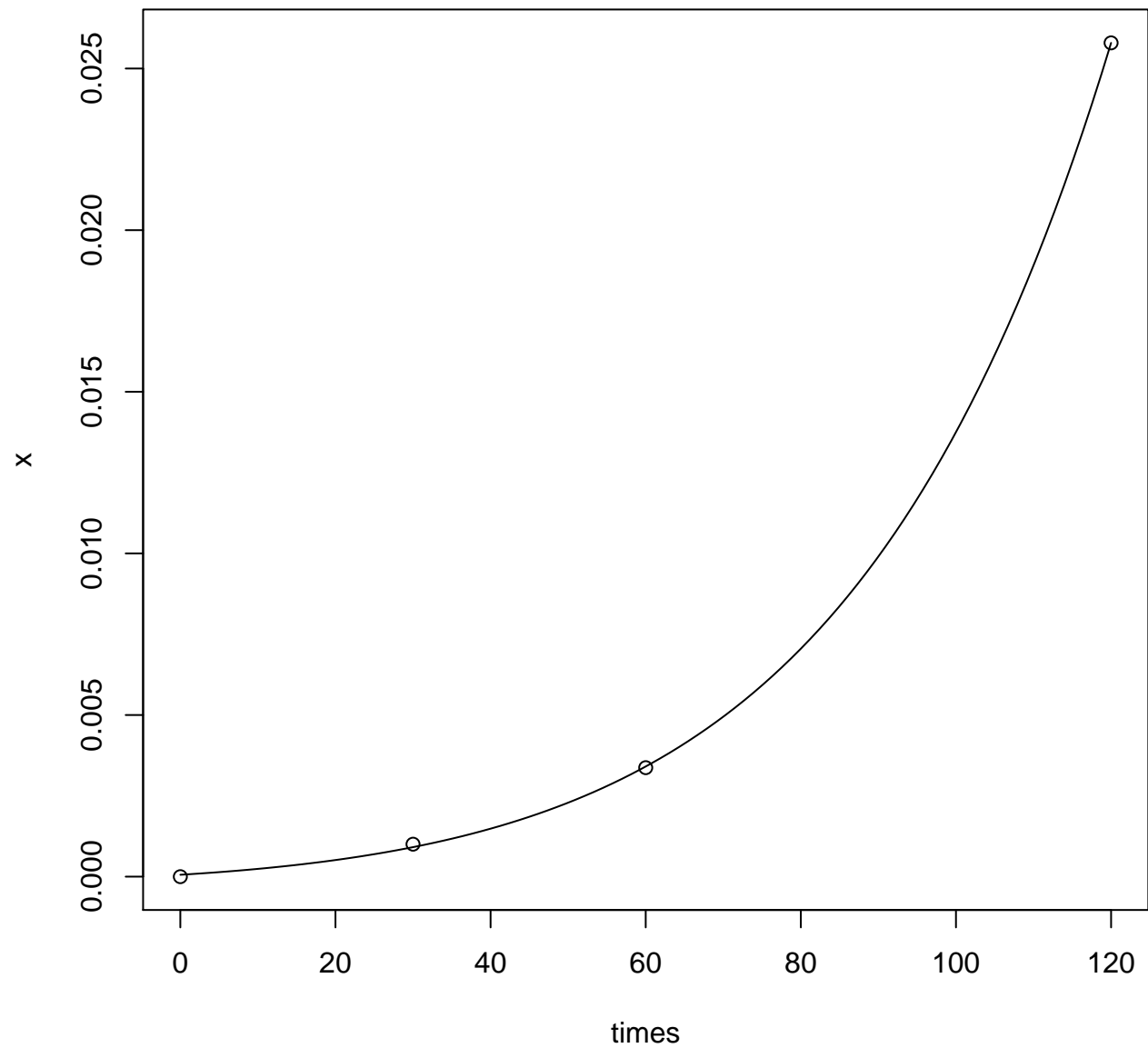

Supplement: Supplementary file 1 [file metabolites-10-00150-s001.zip › Supplementary Code S1/Asp-Thr/AL7/fit_input/res_fit_enr_AL7_thr_1.pdf]

## fitting results

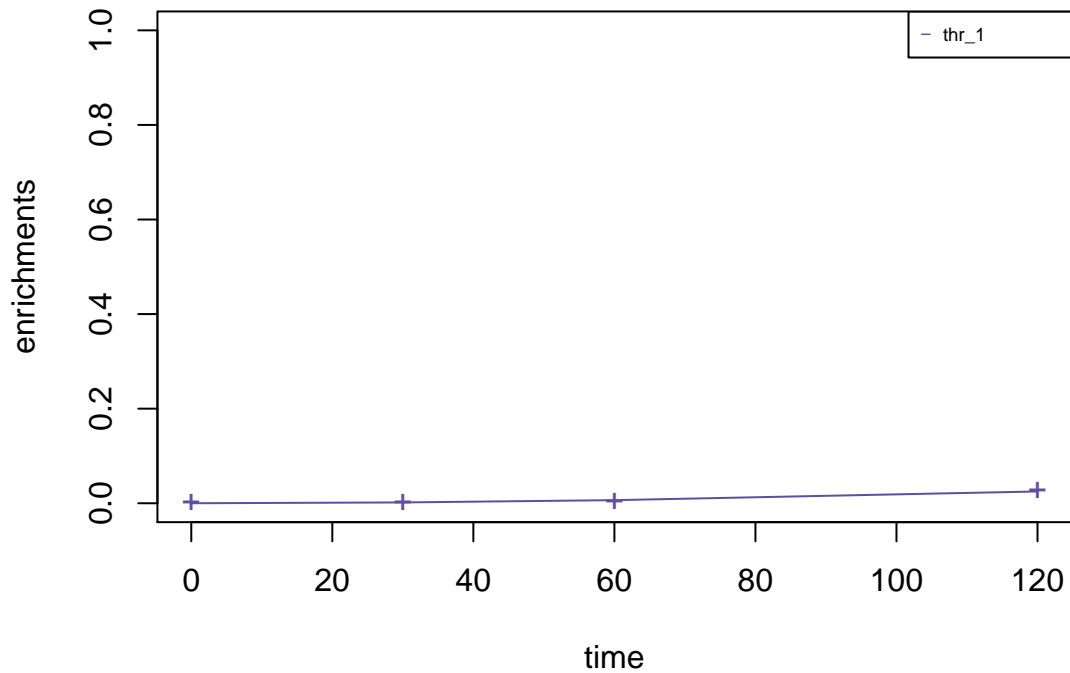

Supplement: Supplementary file 1 [file metabolites-10-00150-s001.zip › Supplementary Code S1/Asp-Thr/AL7/fit_subnet_fitthr/results.pdf]

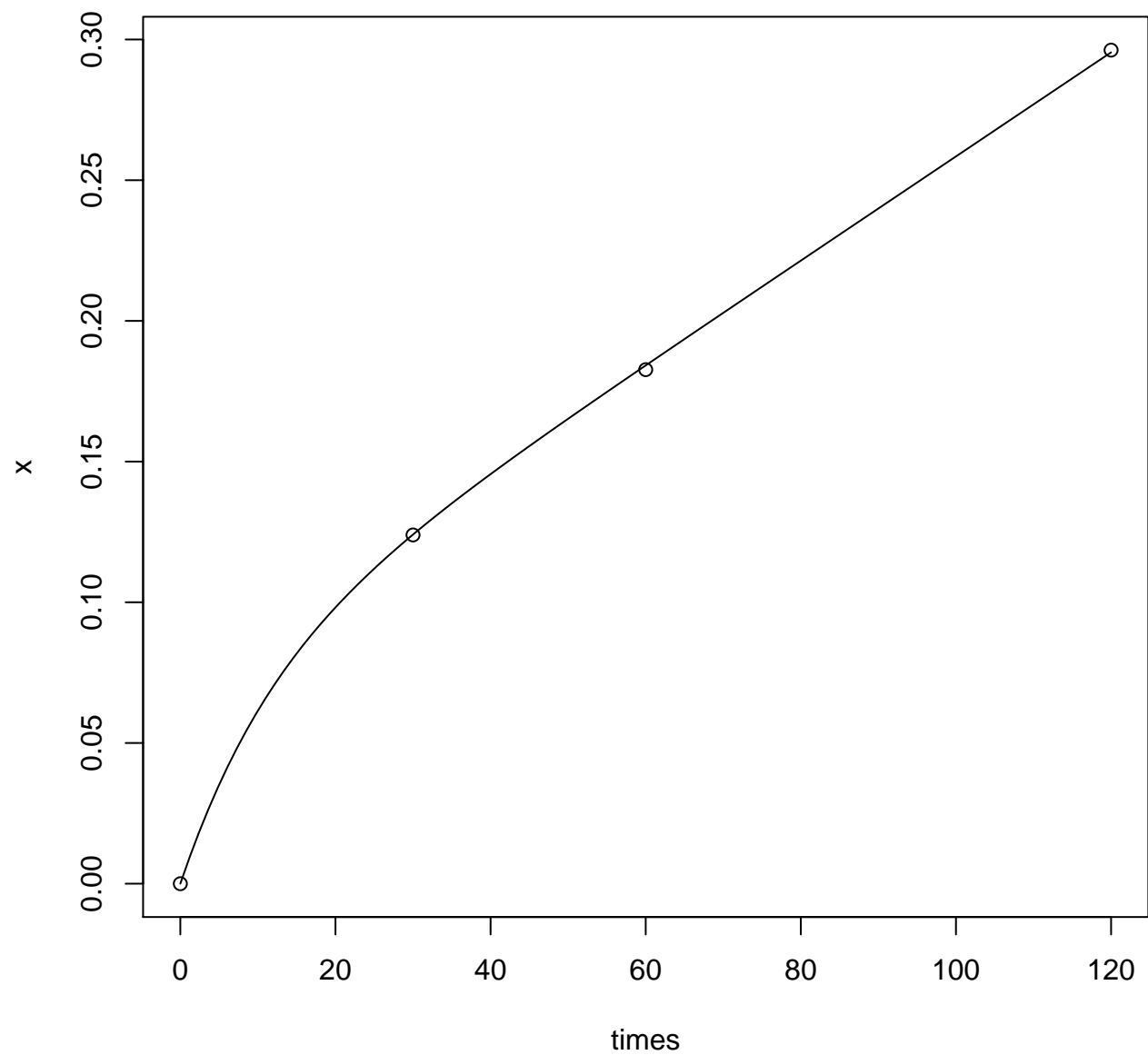

Supplement: Supplementary file 1 [file metabolites-10-00150-s001.zip › Supplementary Code S1/Asp-Thr/BL11/fit_input/res_fit_enr_BL11_asp_1.pdf]

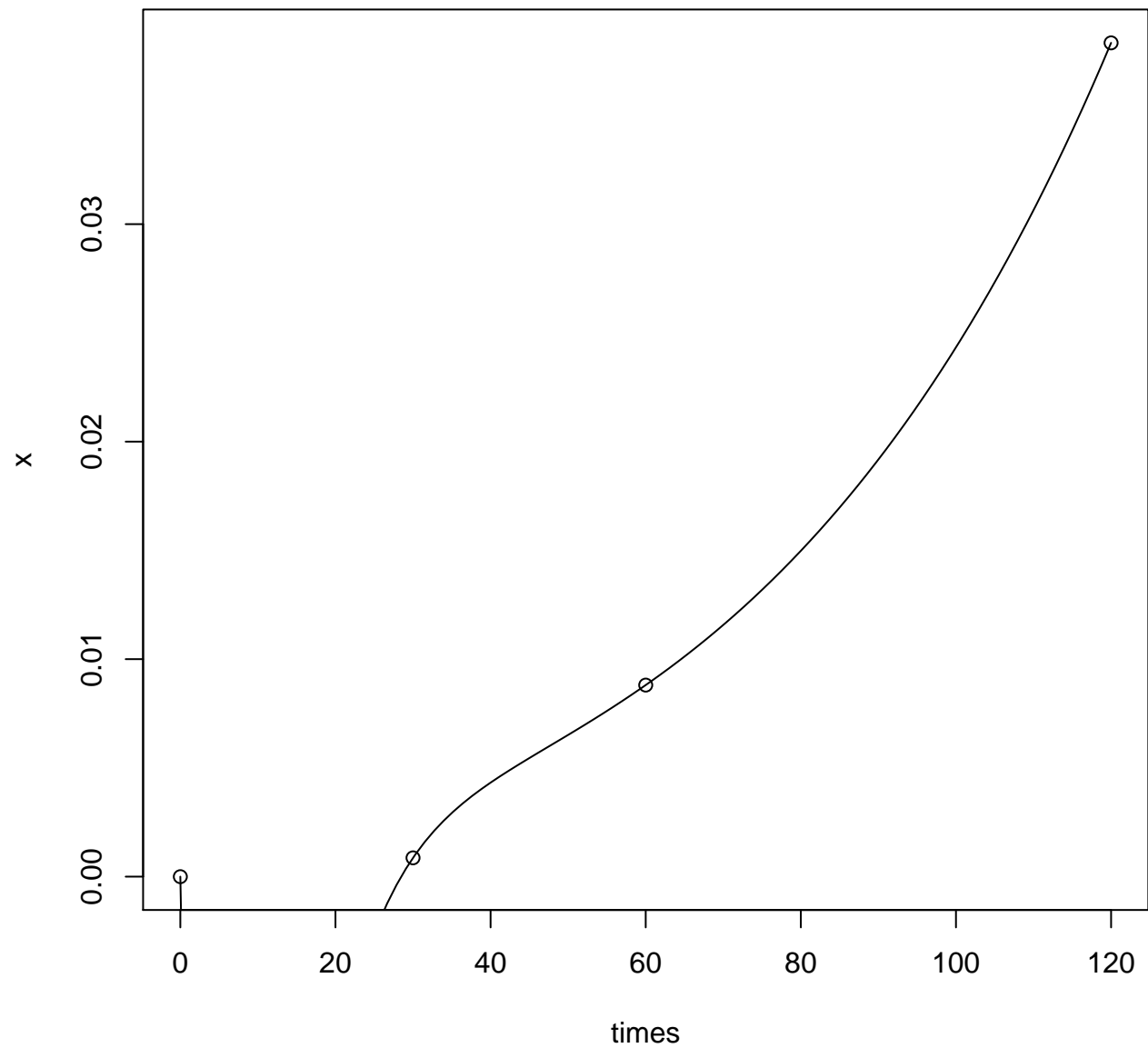

Supplement: Supplementary file 1 [file metabolites-10-00150-s001.zip › Supplementary Code S1/Asp-Thr/BL11/fit_input/res_fit_enr_BL11_thr_1.pdf]

## fitting results

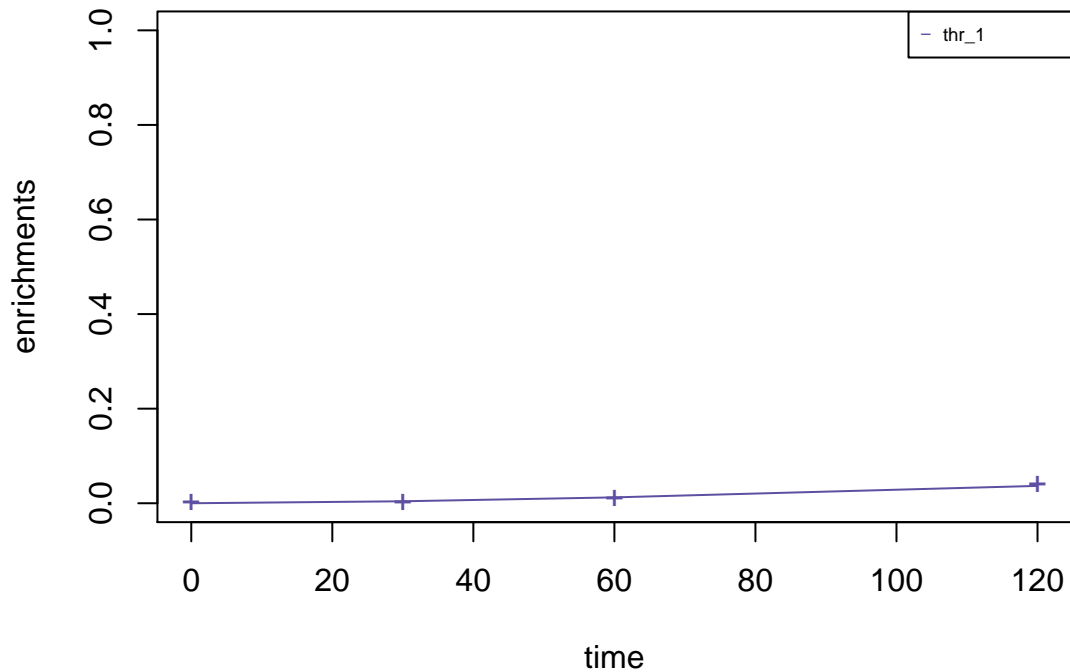

Supplement: Supplementary file 1 [file metabolites-10-00150-s001.zip › Supplementary Code S1/Asp-Thr/BL11/fit_subnet_fitthr/results.pdf]

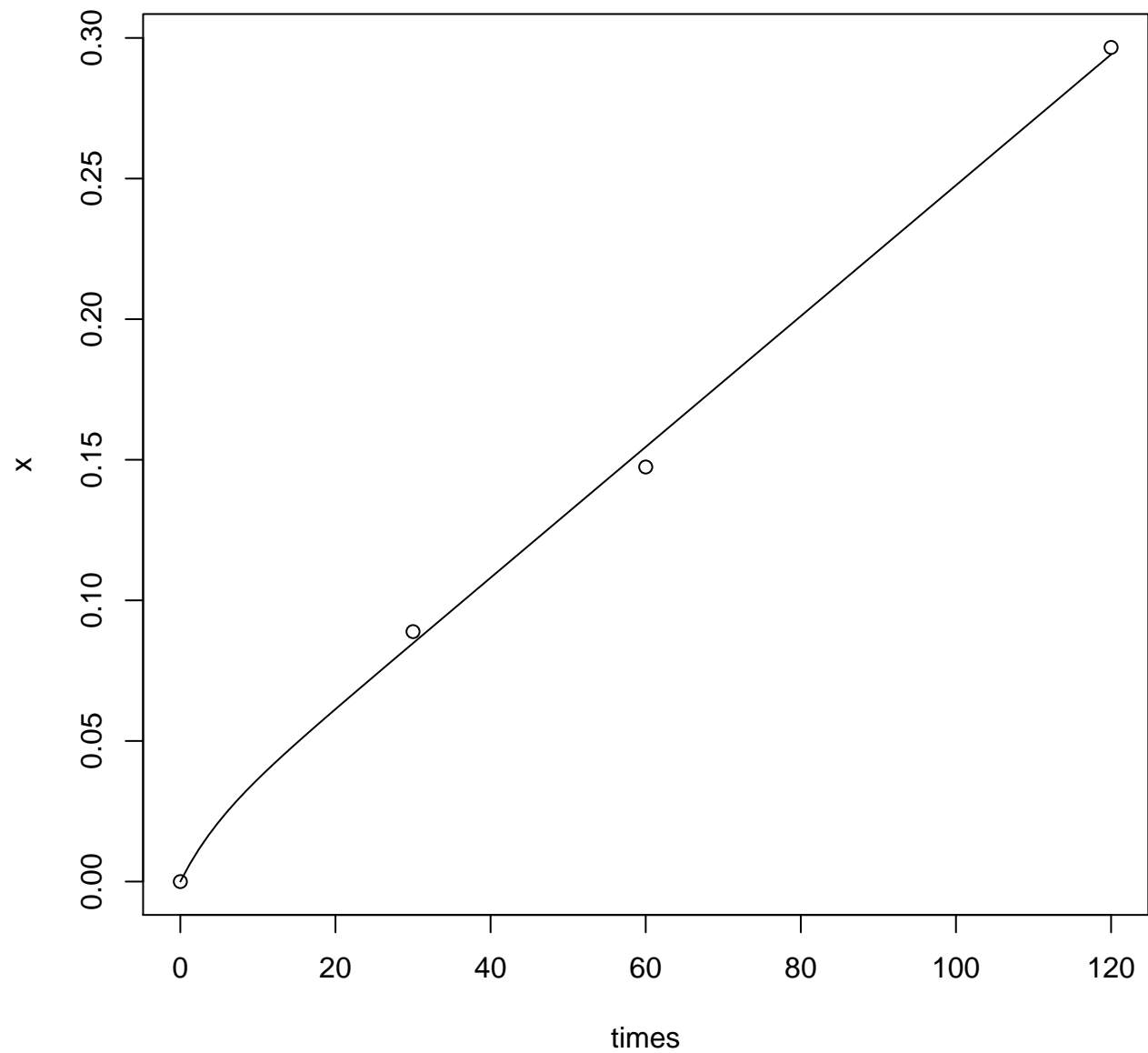

Supplement: Supplementary file 1 [file metabolites-10-00150-s001.zip › Supplementary Code S1/Asp-Thr/BL15/fit_input/res_fit_enr_BL15_asp_1.pdf]

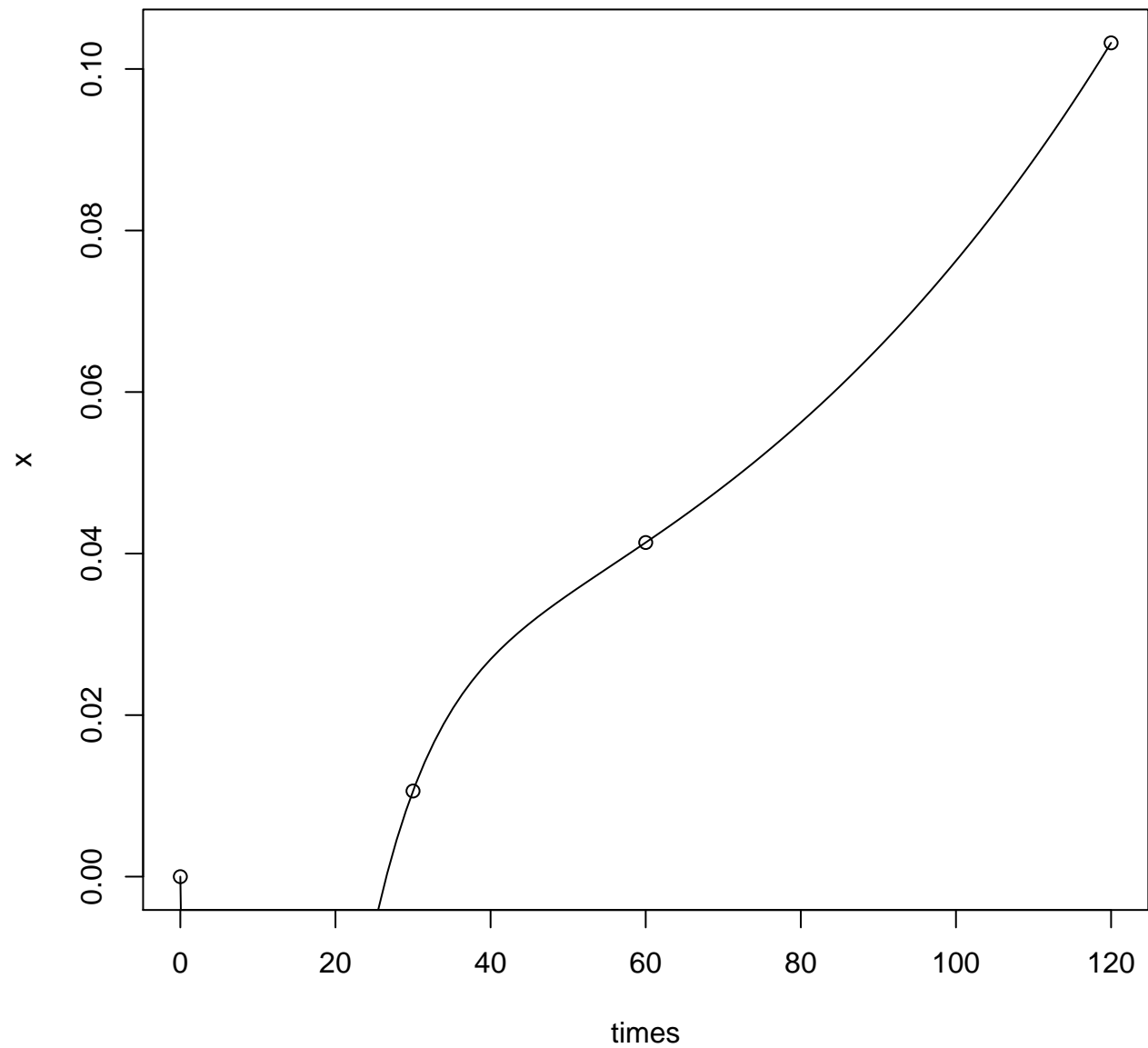

Supplement: Supplementary file 1 [file metabolites-10-00150-s001.zip › Supplementary Code S1/Asp-Thr/BL15/fit_input/res_fit_enr_BL15_thr_1.pdf]

## fitting results

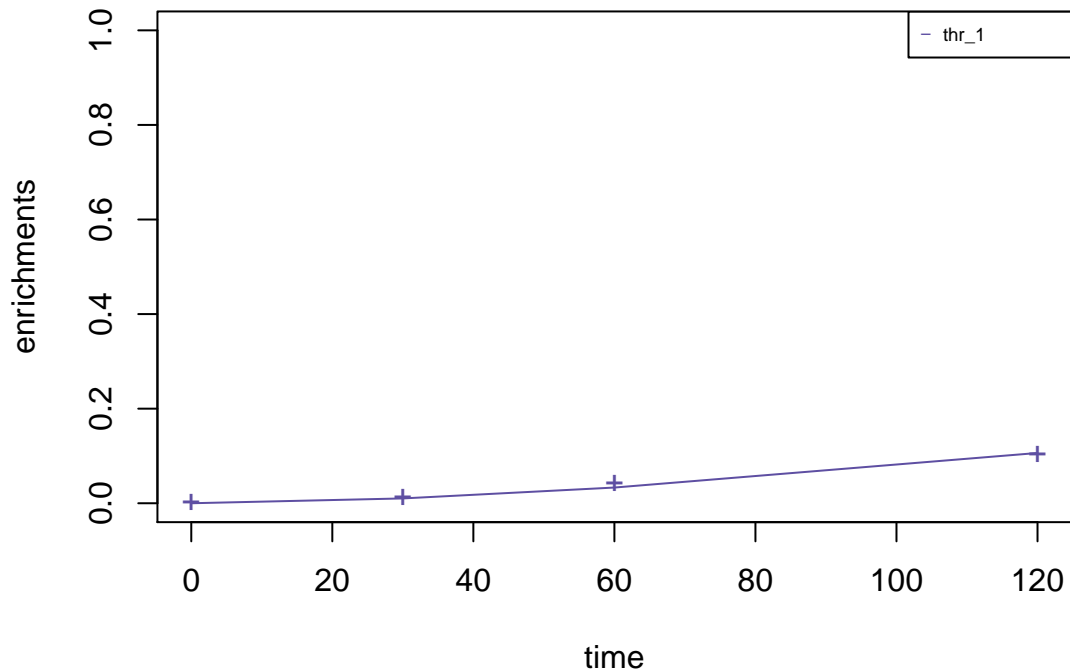

Supplement: Supplementary file 1 [file metabolites-10-00150-s001.zip › Supplementary Code S1/Asp-Thr/BL15/fit_subnet_fitthr/results.pdf]

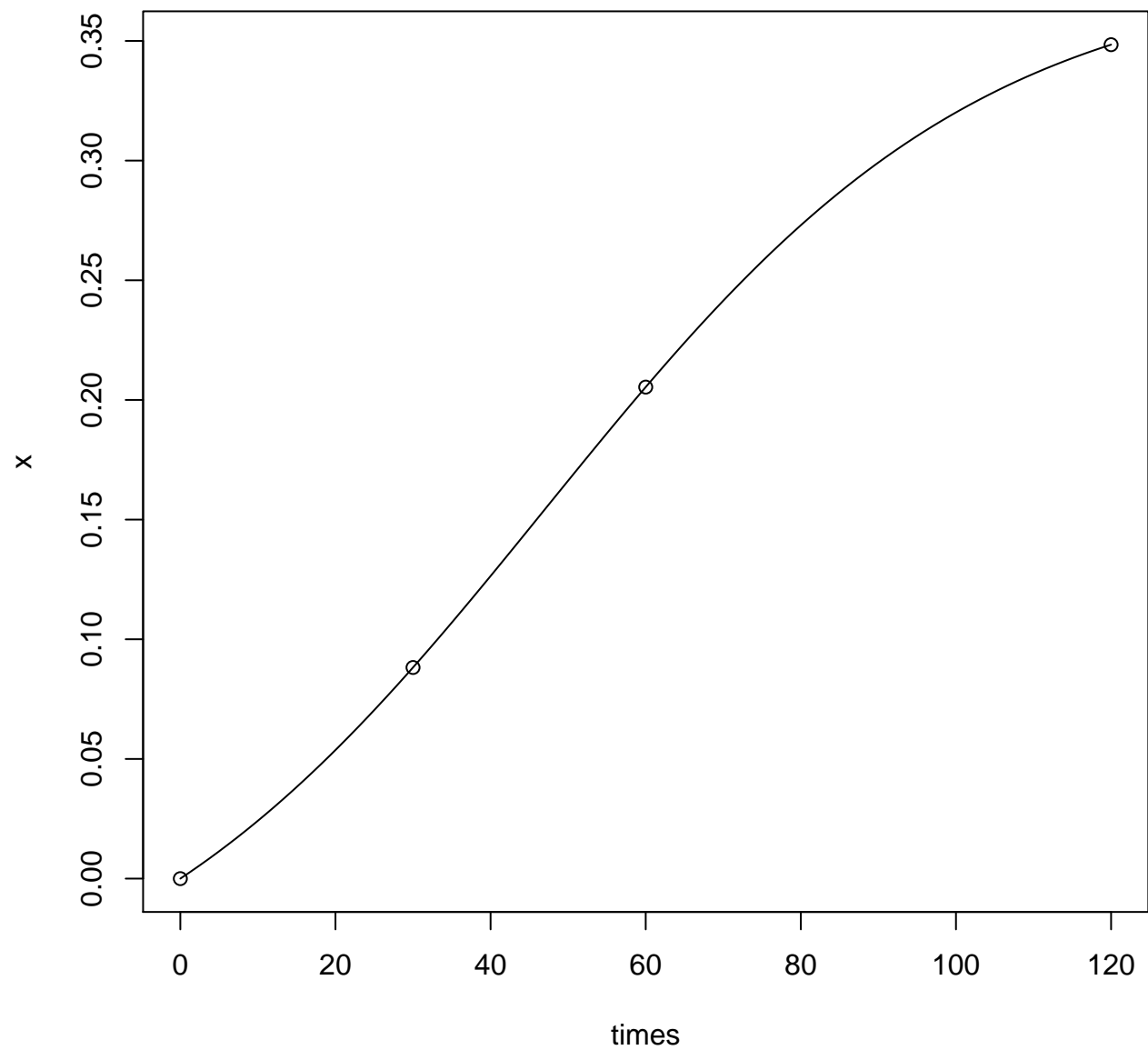

Supplement: Supplementary file 1 [file metabolites-10-00150-s001.zip › Supplementary Code S1/Asp-Thr/BL3/fit_input/res_fit_enr_BL3_asp_1.pdf]

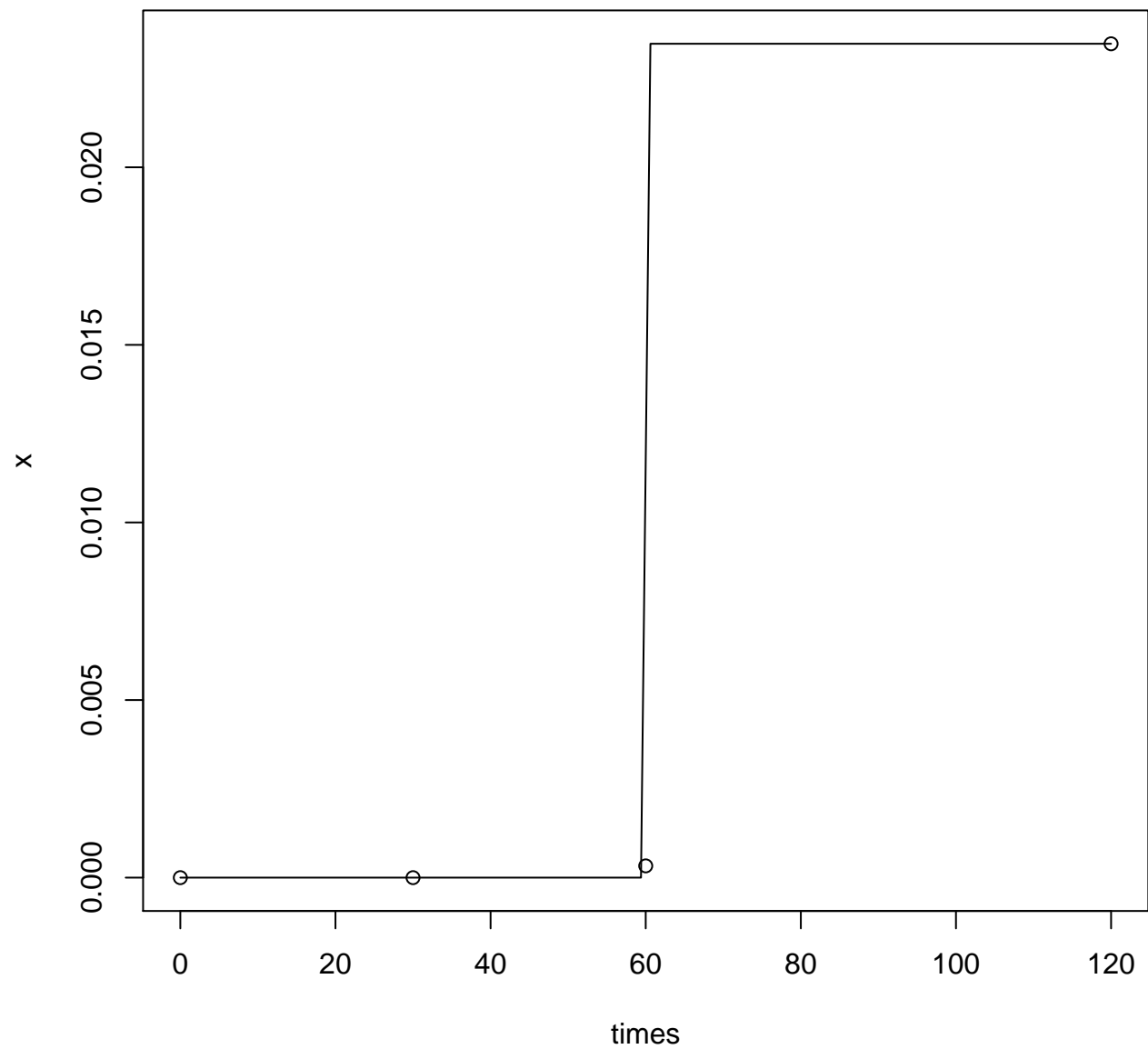

Supplement: Supplementary file 1 [file metabolites-10-00150-s001.zip › Supplementary Code S1/Asp-Thr/BL3/fit_input/res_fit_enr_BL3_thr_1.pdf]

## fitting results

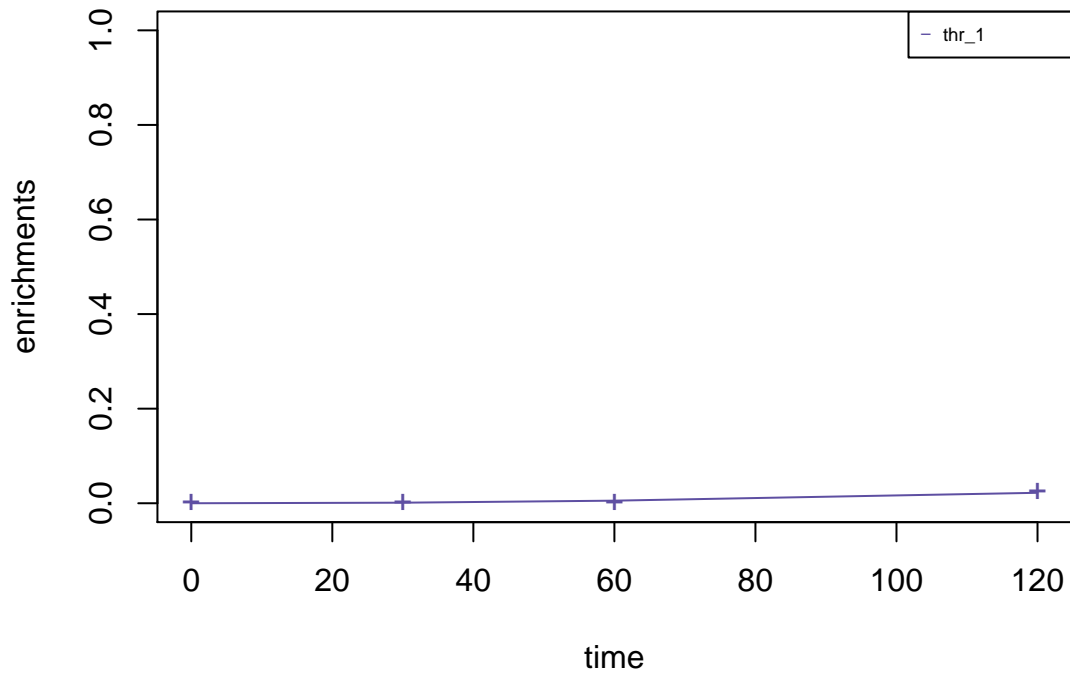

Supplement: Supplementary file 1 [file metabolites-10-00150-s001.zip › Supplementary Code S1/Asp-Thr/BL3/fit_subnet_fitthr/results.pdf]

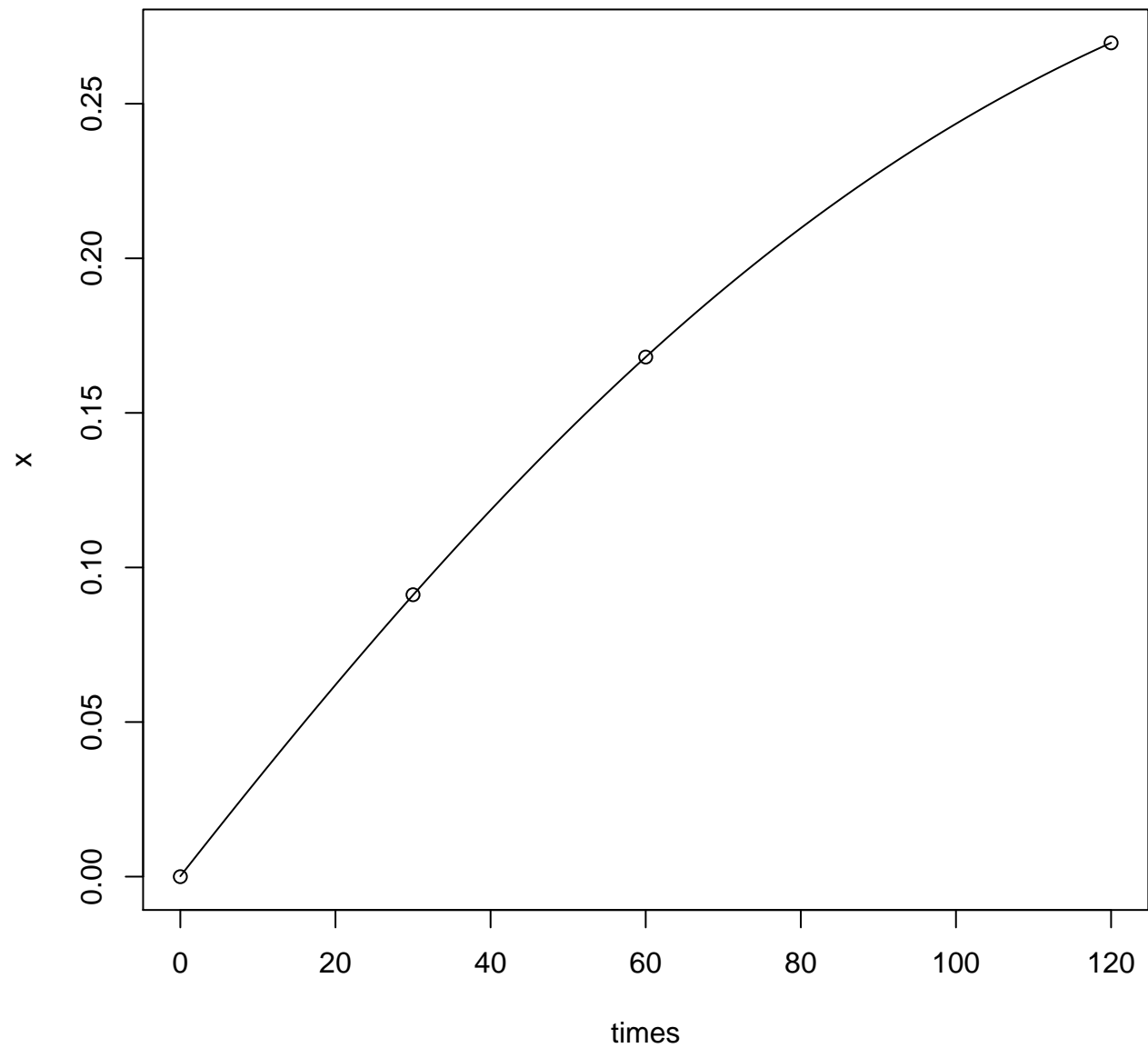

Supplement: Supplementary file 1 [file metabolites-10-00150-s001.zip › Supplementary Code S1/Asp-Thr/BL7/fit_input/res_fit_enr_BL7_asp_1.pdf]

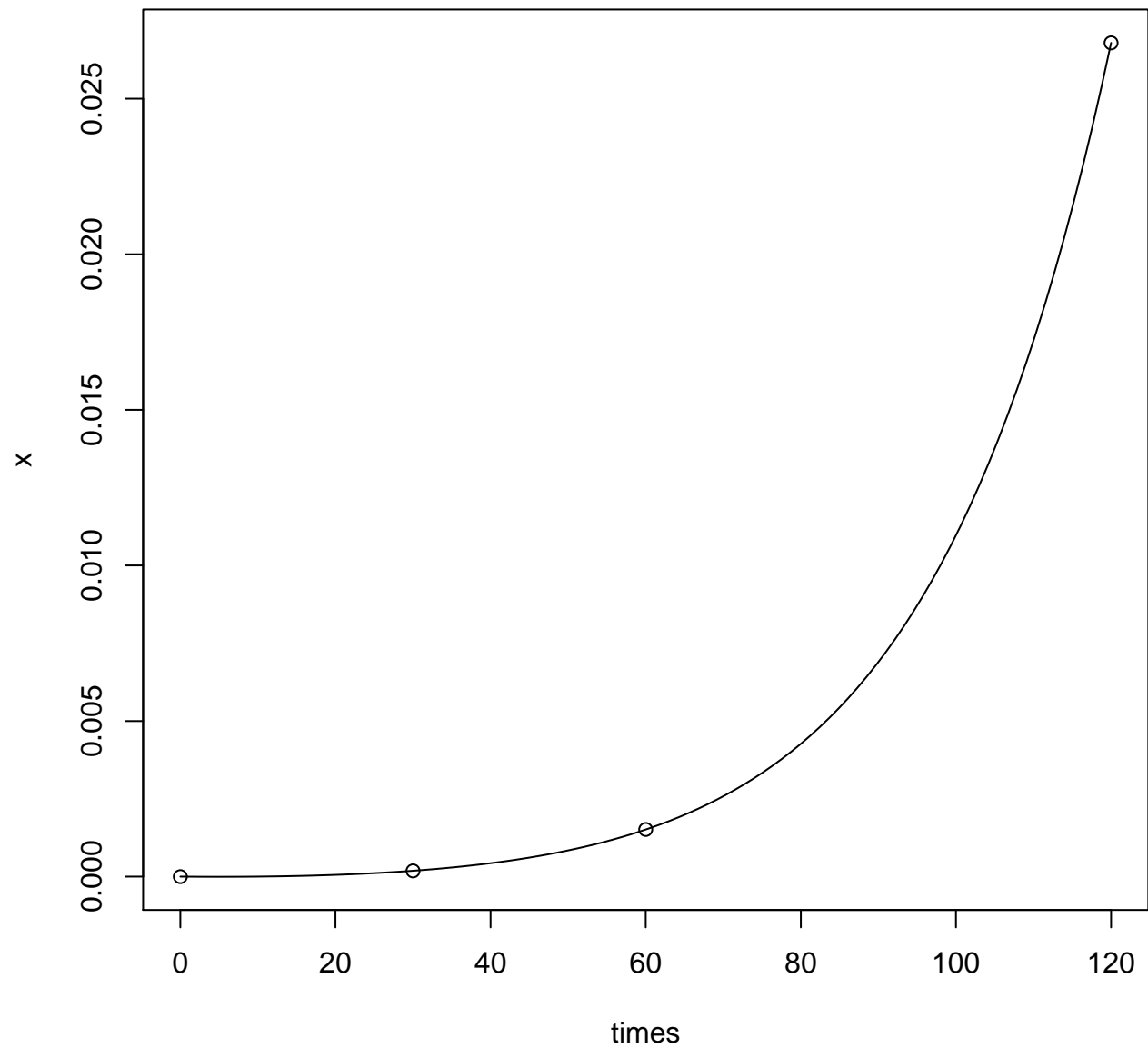

Supplement: Supplementary file 1 [file metabolites-10-00150-s001.zip › Supplementary Code S1/Asp-Thr/BL7/fit_input/res_fit_enr_BL7_thr_1.pdf]

## fitting results

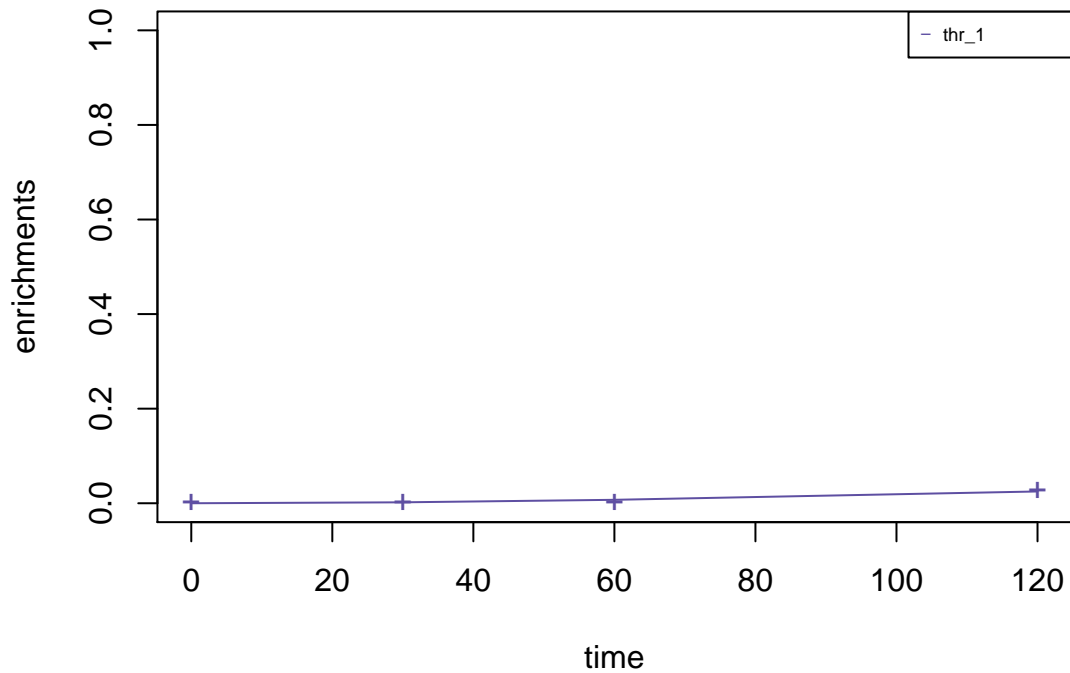

Supplement: Supplementary file 1 [file metabolites-10-00150-s001.zip › Supplementary Code S1/Asp-Thr/BL7/fit_subnet_fitthr/results.pdf]

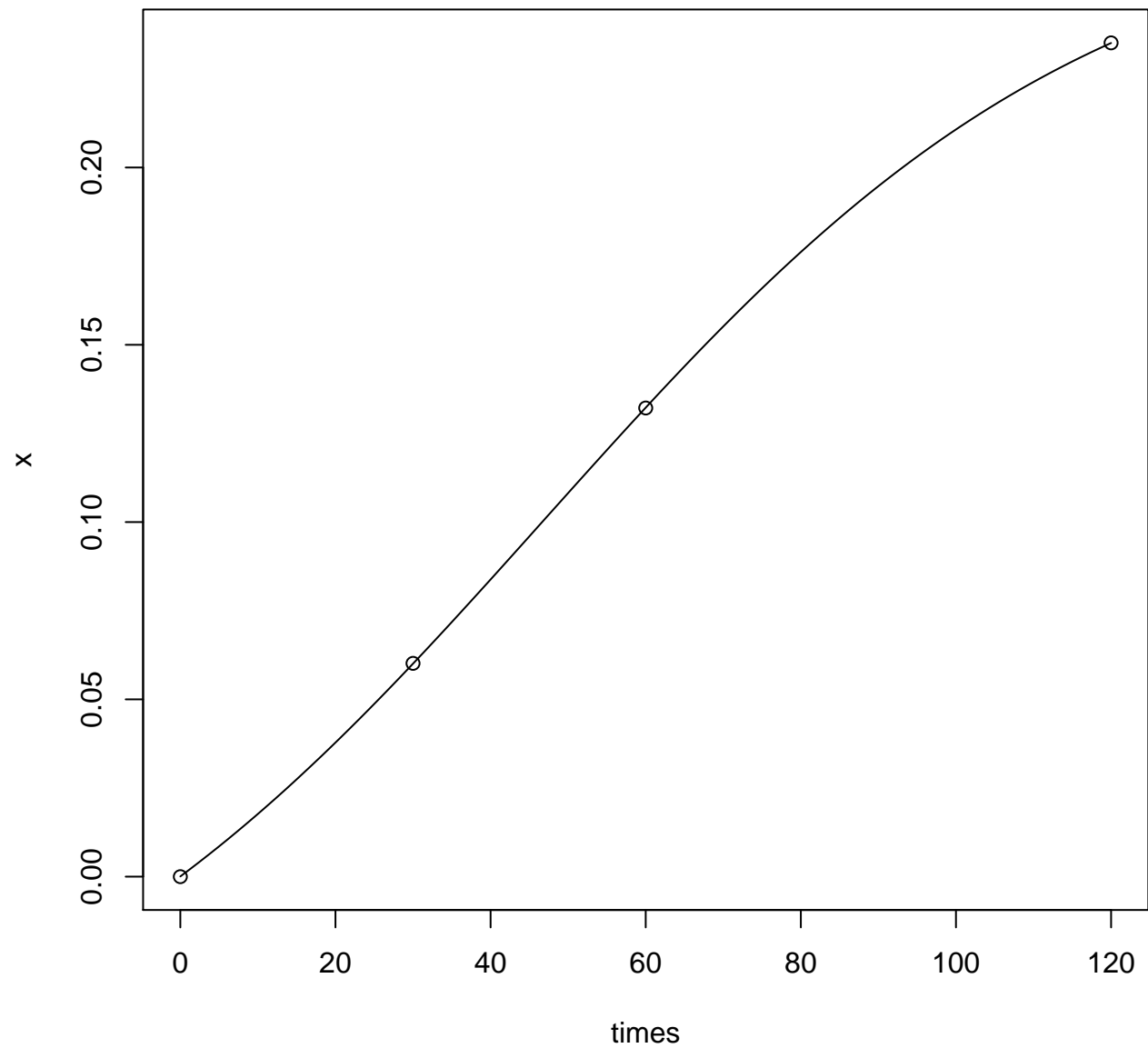

Supplement: Supplementary file 1 [file metabolites-10-00150-s001.zip › Supplementary Code S1/Asp-Thr/CL11/fit_input/res_fit_enr_CL11_asp_1.pdf]

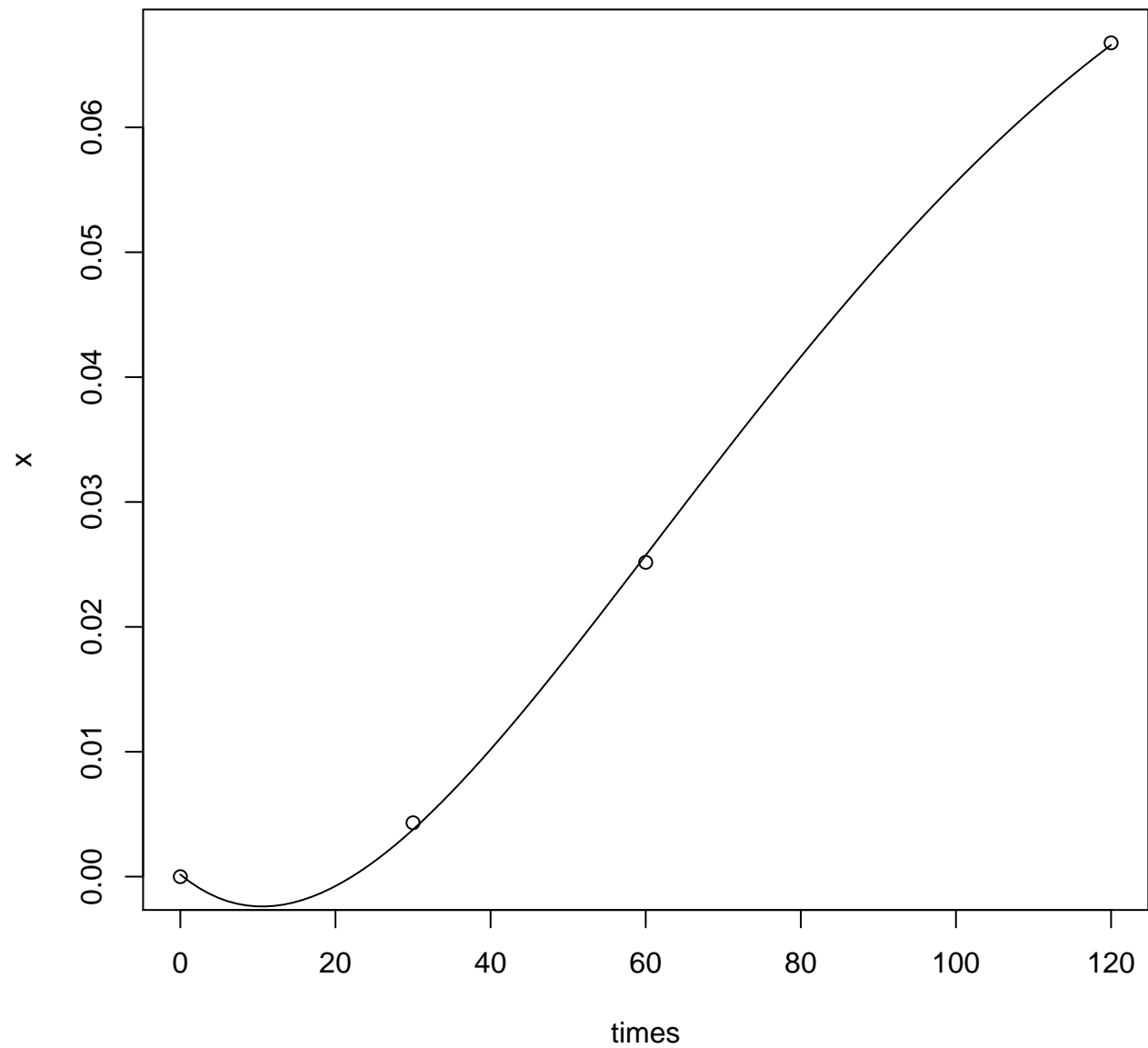

Supplement: Supplementary file 1 [file metabolites-10-00150-s001.zip › Supplementary Code S1/Asp-Thr/CL11/fit_input/res_fit_enr_CL11_thr_1.pdf]

## fitting results

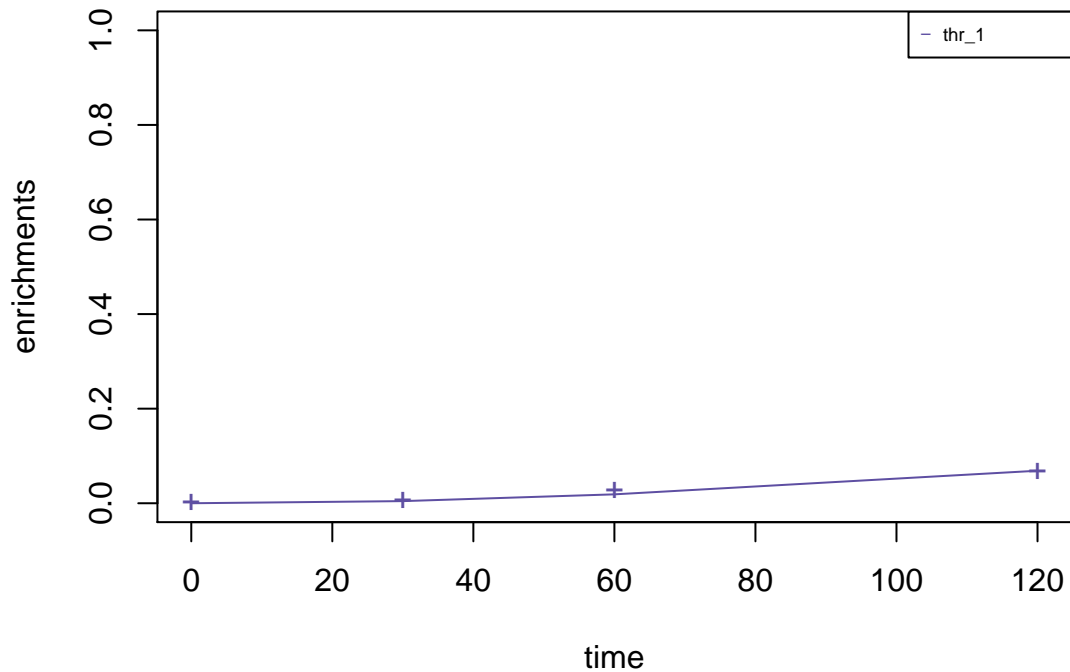

Supplement: Supplementary file 1 [file metabolites-10-00150-s001.zip › Supplementary Code S1/Asp-Thr/CL11/fit_subnet_fitthr/results.pdf]

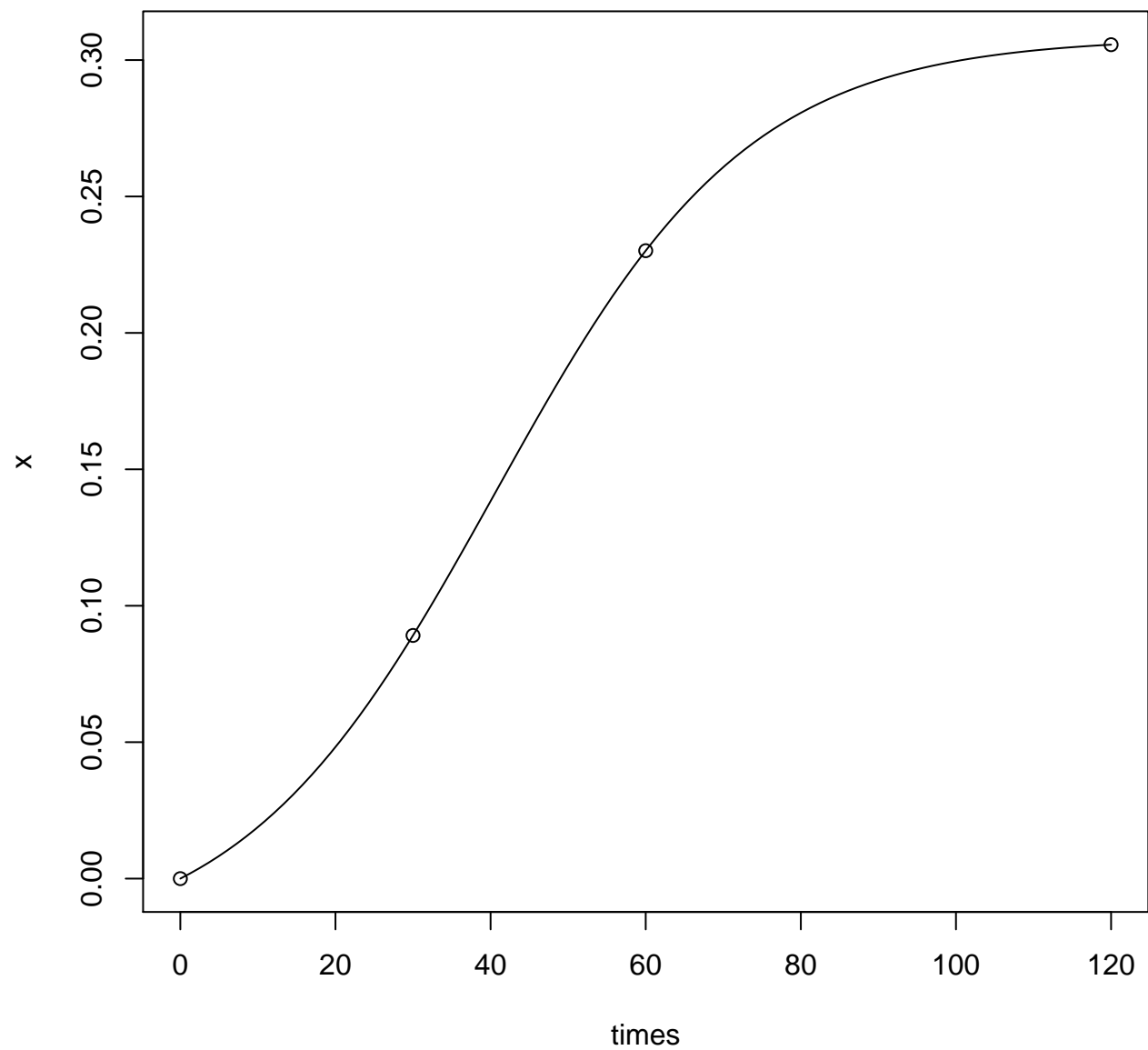

Supplement: Supplementary file 1 [file metabolites-10-00150-s001.zip › Supplementary Code S1/Asp-Thr/CL15/fit_input/res_fit_enr_CL15_asp_1.pdf]

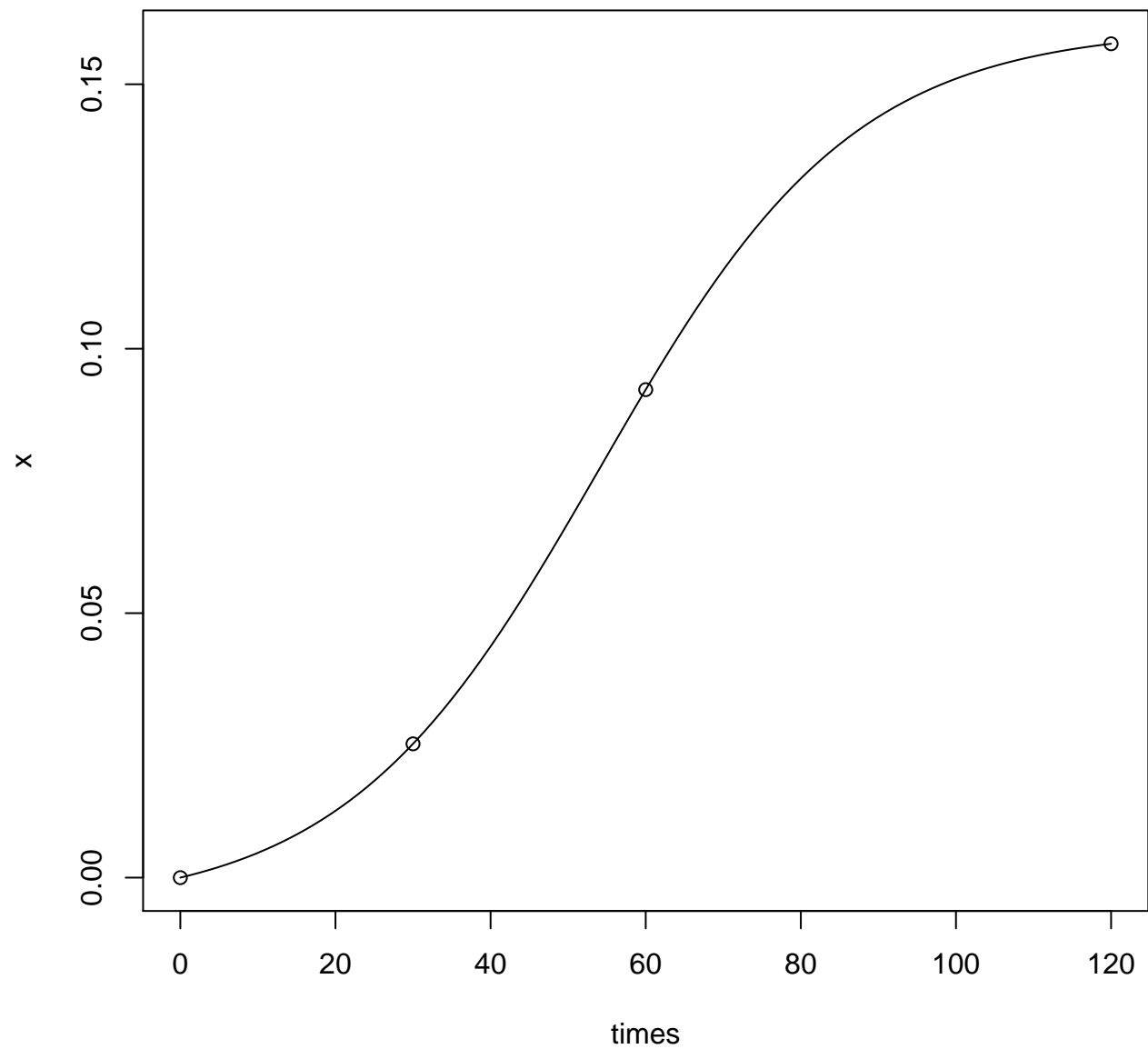

Supplement: Supplementary file 1 [file metabolites-10-00150-s001.zip › Supplementary Code S1/Asp-Thr/CL15/fit_input/res_fit_enr_CL15_thr_1.pdf]

## fitting results

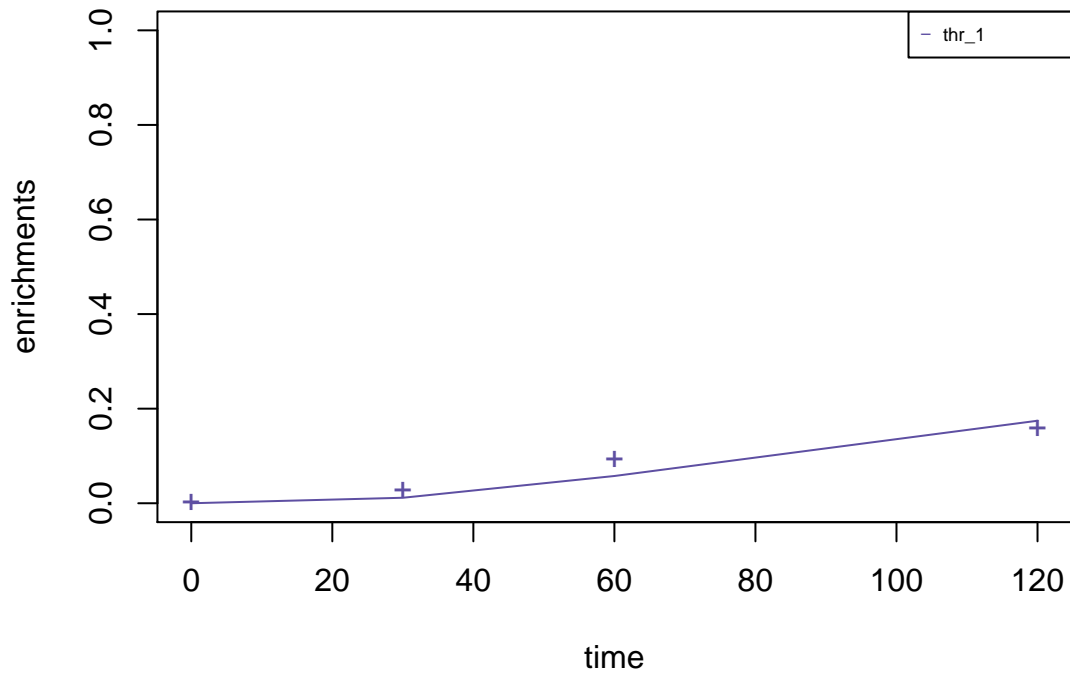

Supplement: Supplementary file 1 [file metabolites-10-00150-s001.zip › Supplementary Code S1/Asp-Thr/CL15/fit_subnet_fitthr/results.pdf]

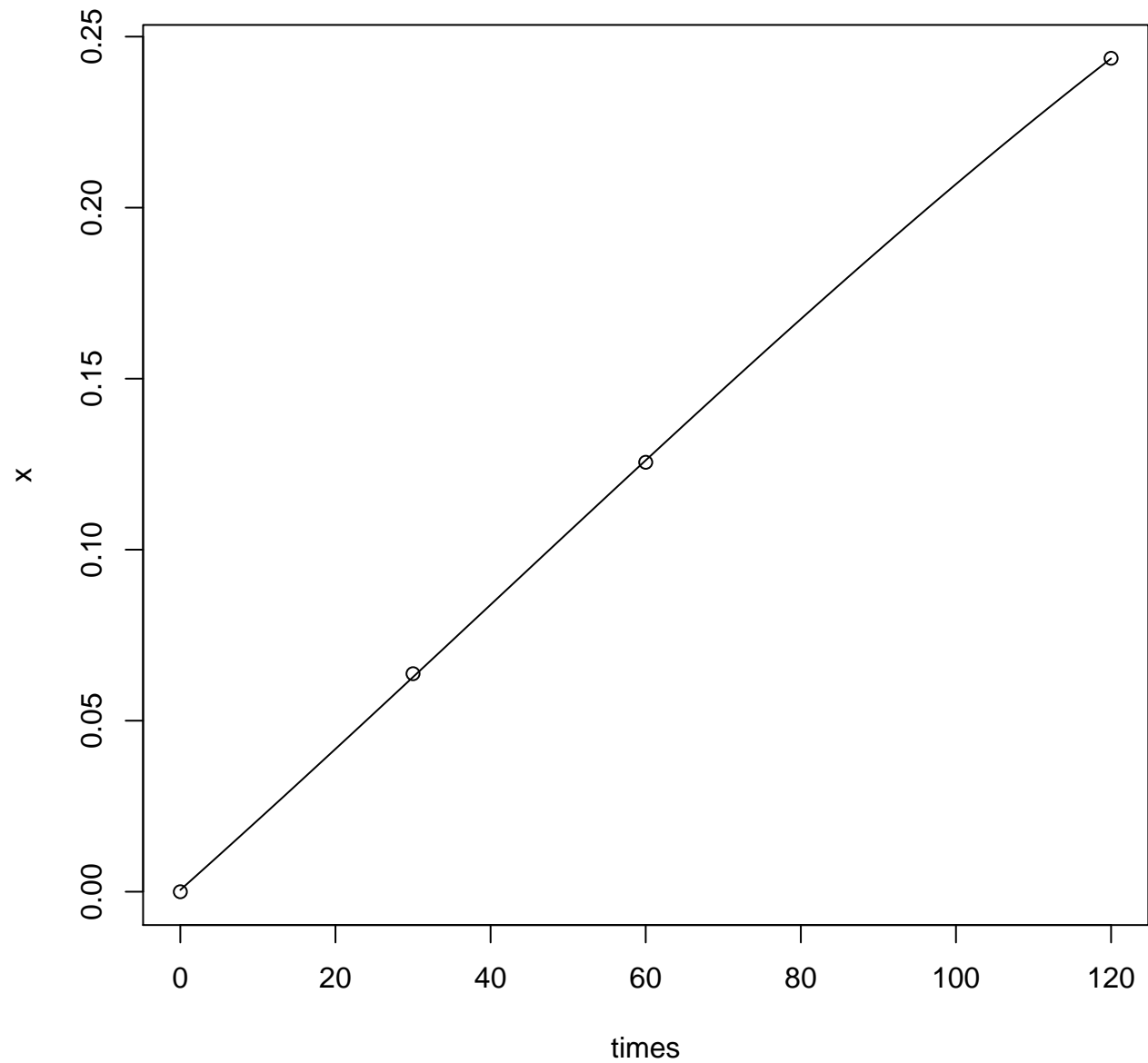

Supplement: Supplementary file 1 [file metabolites-10-00150-s001.zip › Supplementary Code S1/Asp-Thr/CL3/fit_input/res_fit_enr_CL3_asp_1.pdf]

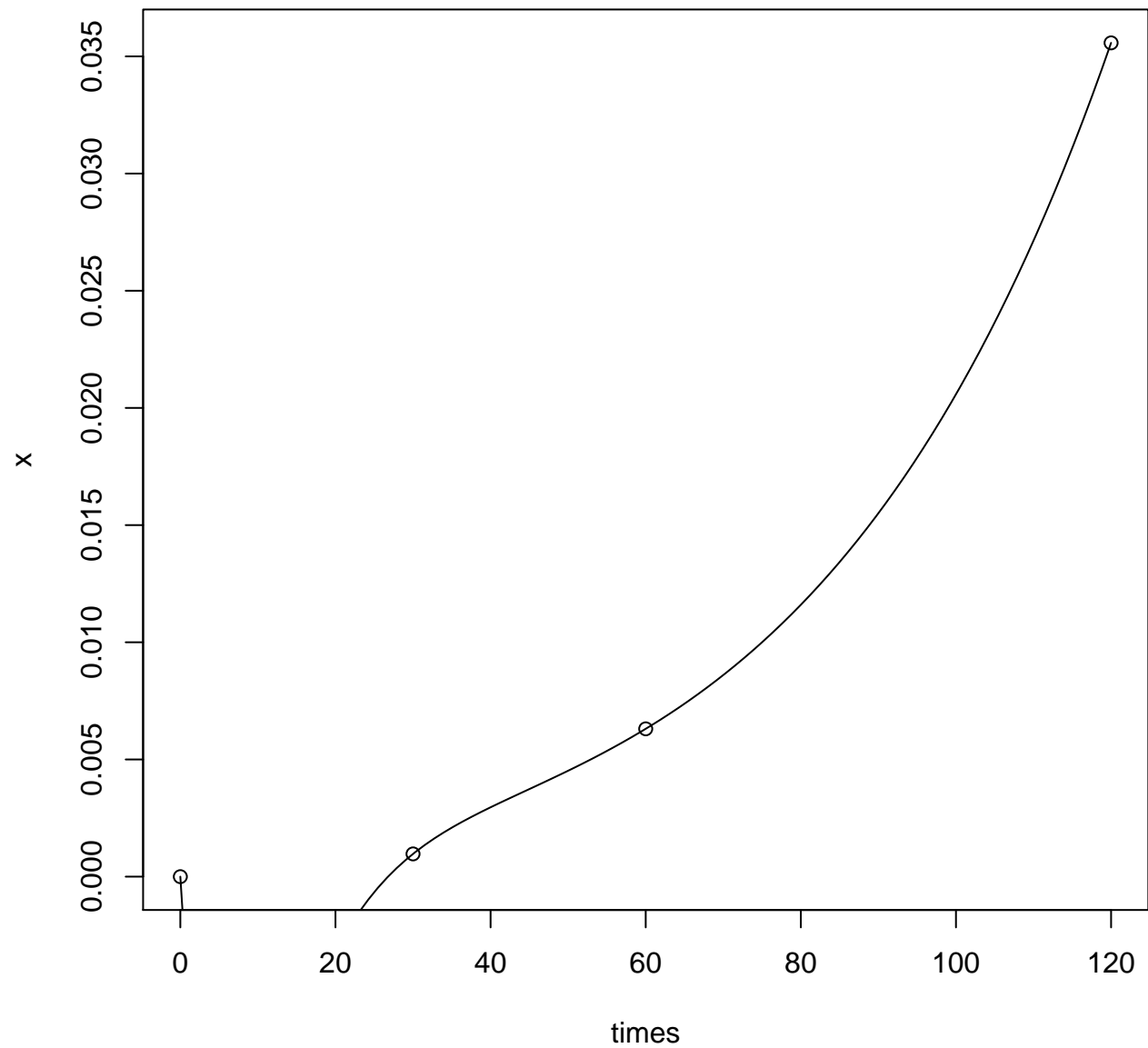

Supplement: Supplementary file 1 [file metabolites-10-00150-s001.zip › Supplementary Code S1/Asp-Thr/CL3/fit_input/res_fit_enr_CL3_thr_1.pdf]

## fitting results

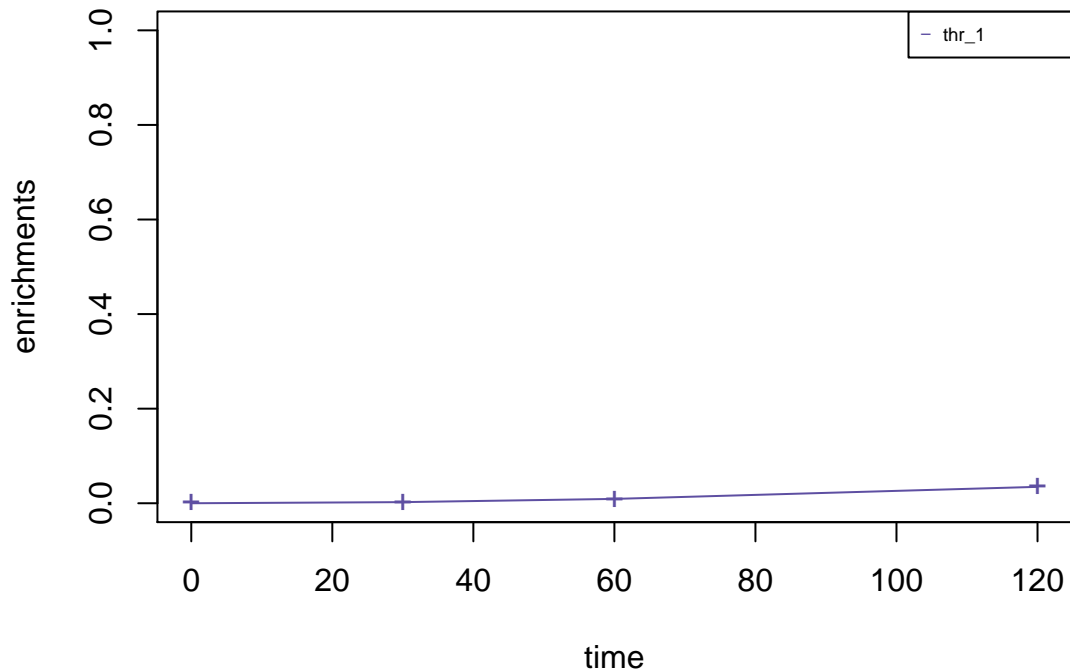

Supplement: Supplementary file 1 [file metabolites-10-00150-s001.zip › Supplementary Code S1/Asp-Thr/CL3/fit_subnet_fitthr/results.pdf]

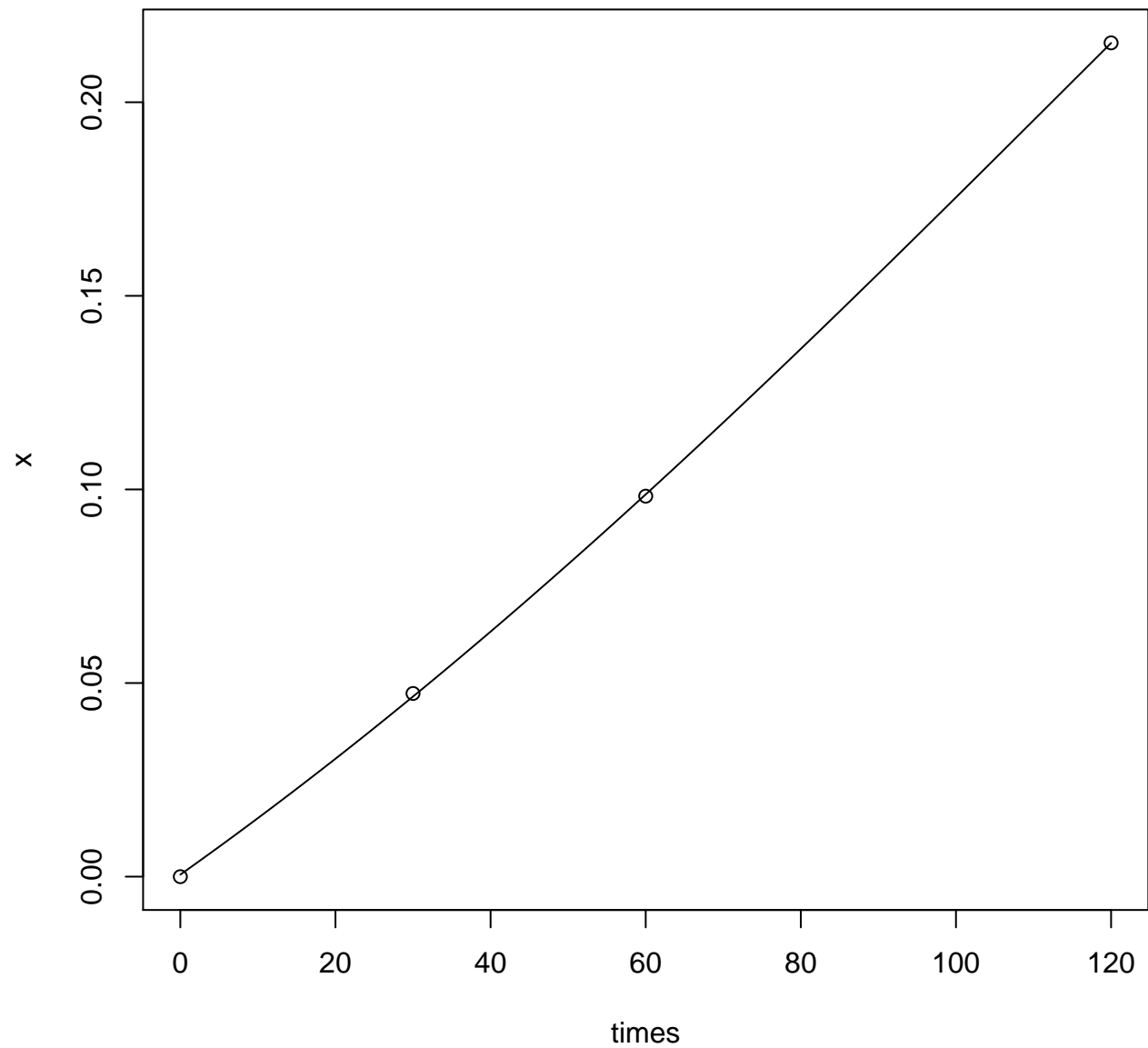

Supplement: Supplementary file 1 [file metabolites-10-00150-s001.zip › Supplementary Code S1/Asp-Thr/CL7/fit_input/res_fit_enr_CL7_asp_1.pdf]

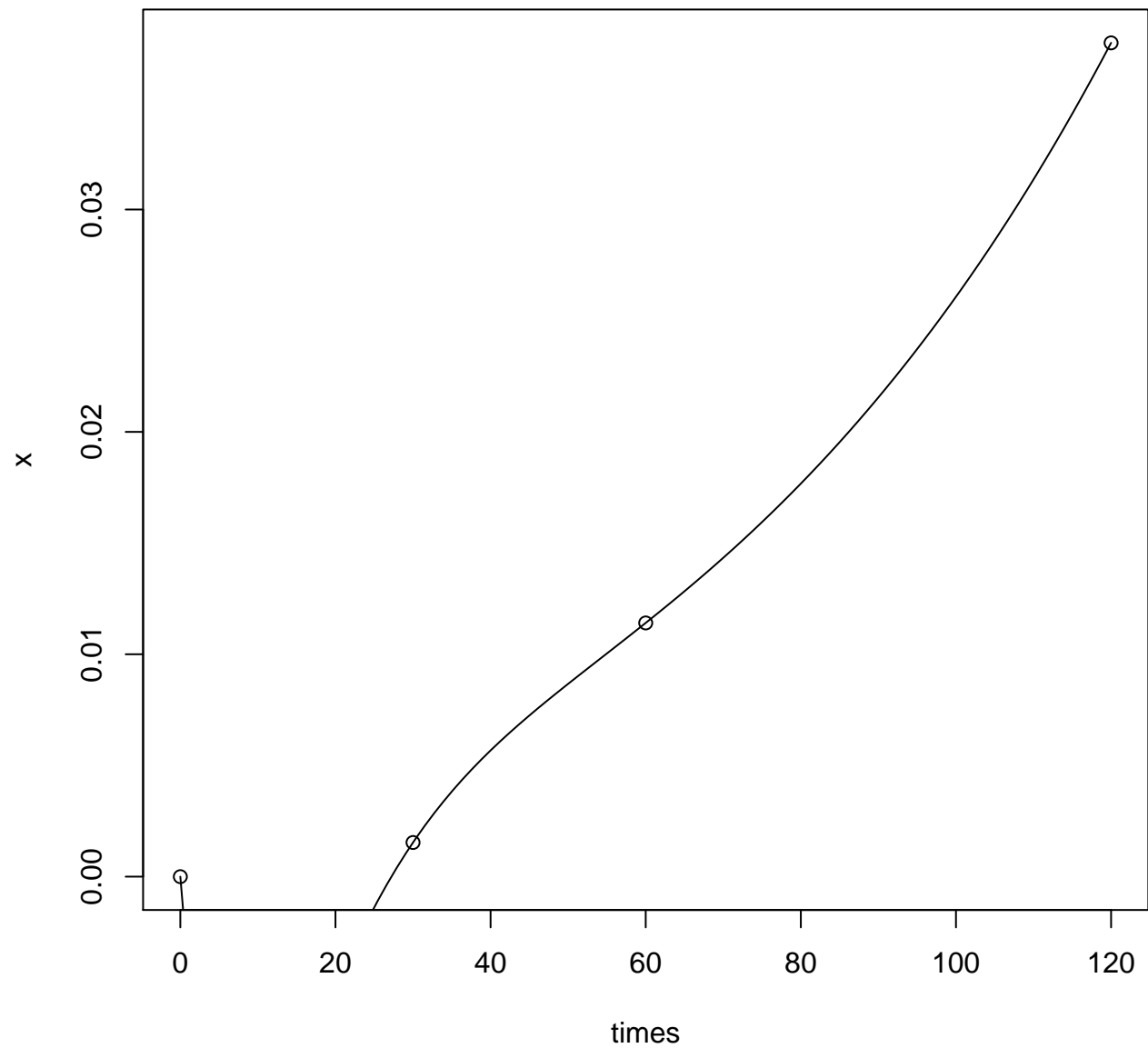

Supplement: Supplementary file 1 [file metabolites-10-00150-s001.zip › Supplementary Code S1/Asp-Thr/CL7/fit_input/res_fit_enr_CL7_thr_1.pdf]

## fitting results

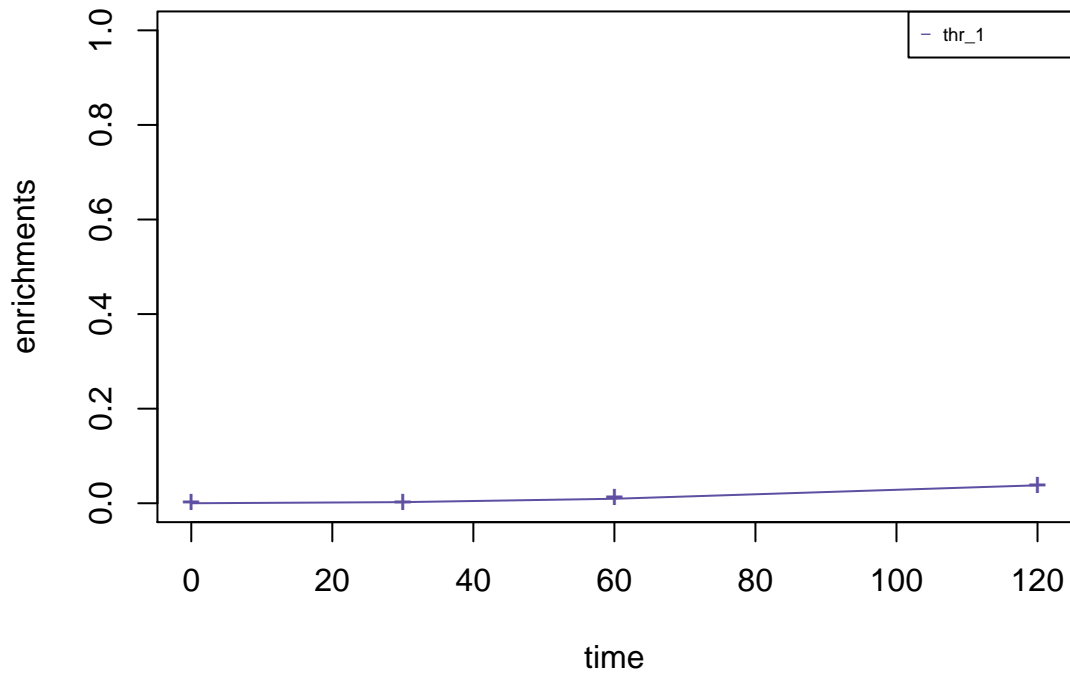

Supplement: Supplementary file 1 [file metabolites-10-00150-s001.zip › Supplementary Code S1/Asp-Thr/CL7/fit_subnet_fitthr/results.pdf]

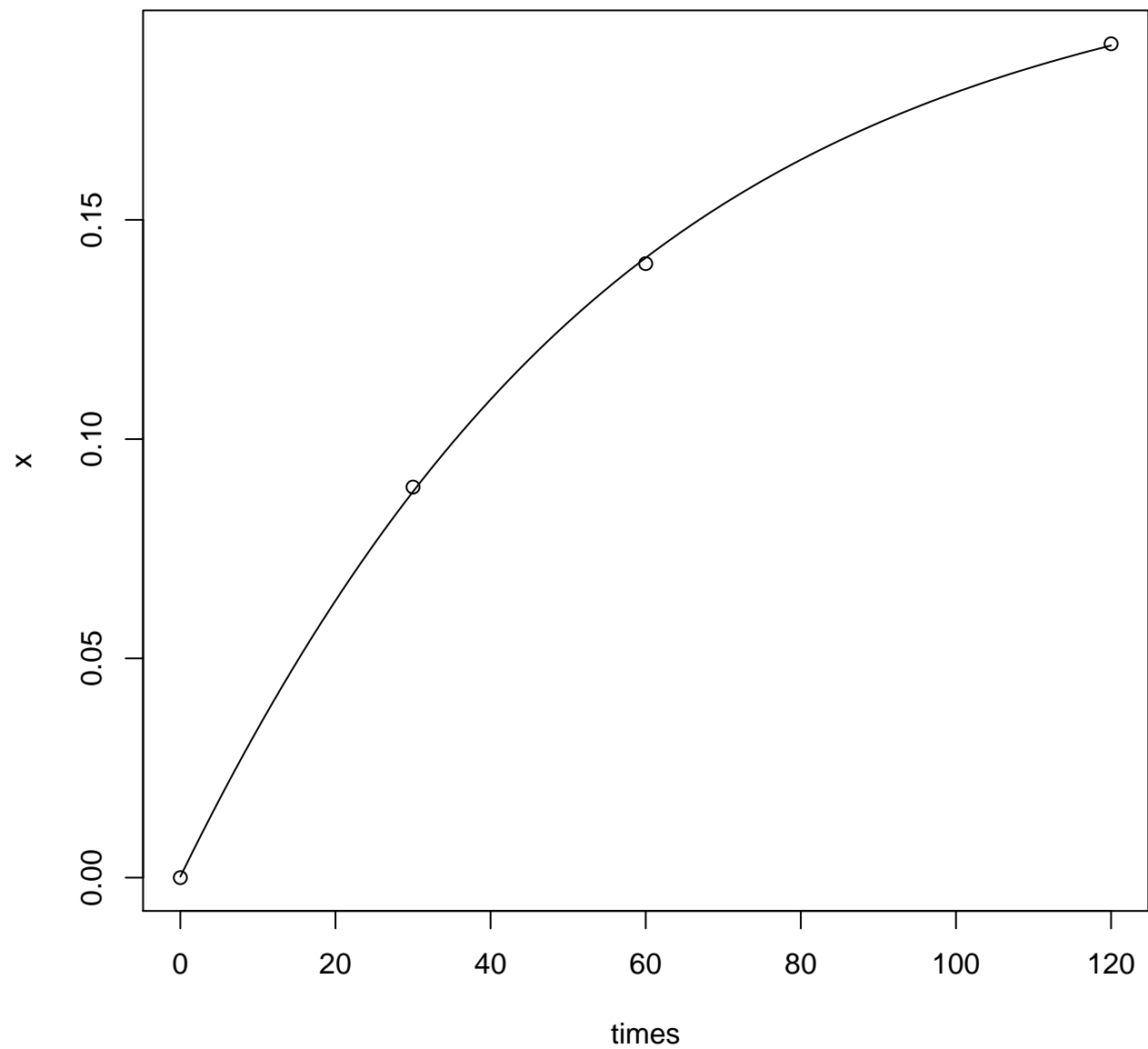

Supplement: Supplementary file 1 [file metabolites-10-00150-s001.zip › Supplementary Code S1/Glu-Pro/AL11/fit_input/res_fit_enr_AL11_glu_1.pdf]

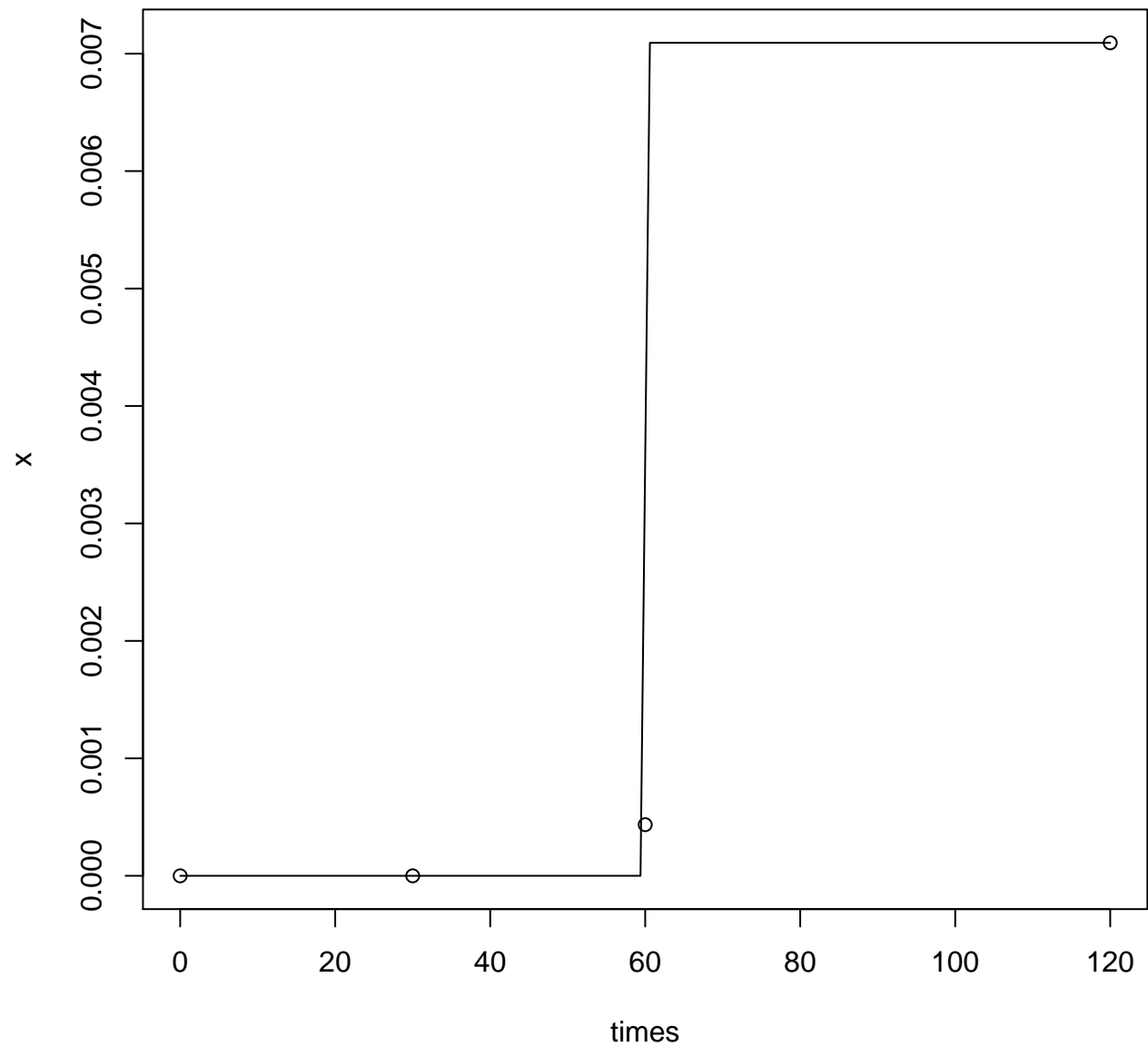

Supplement: Supplementary file 1 [file metabolites-10-00150-s001.zip › Supplementary Code S1/Glu-Pro/AL11/fit_input/res_fit_enr_AL11_pro_1.pdf]

## fitting results

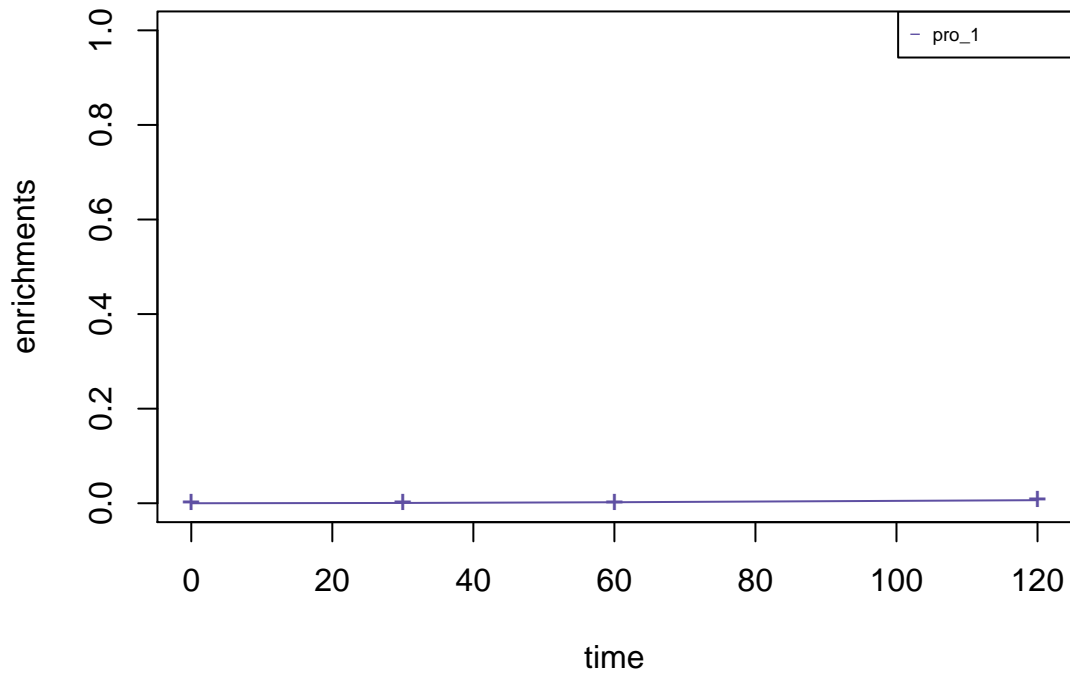

Supplement: Supplementary file 1 [file metabolites-10-00150-s001.zip › Supplementary Code S1/Glu-Pro/AL11/fit_subnet_fitpro/results.pdf]

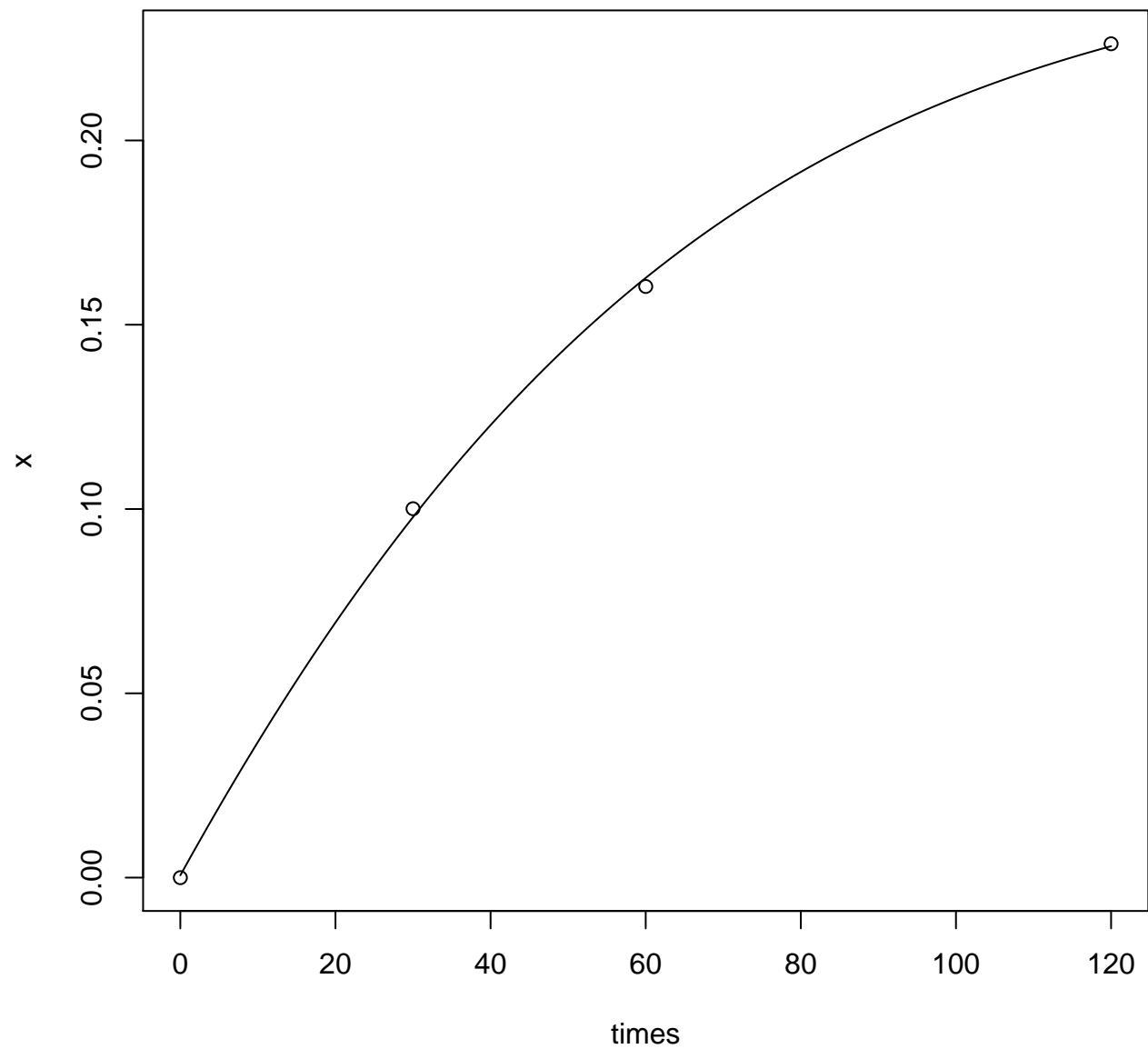

Supplement: Supplementary file 1 [file metabolites-10-00150-s001.zip › Supplementary Code S1/Glu-Pro/AL15/fit_input/res_fit_enr_AL15_glu_1.pdf]

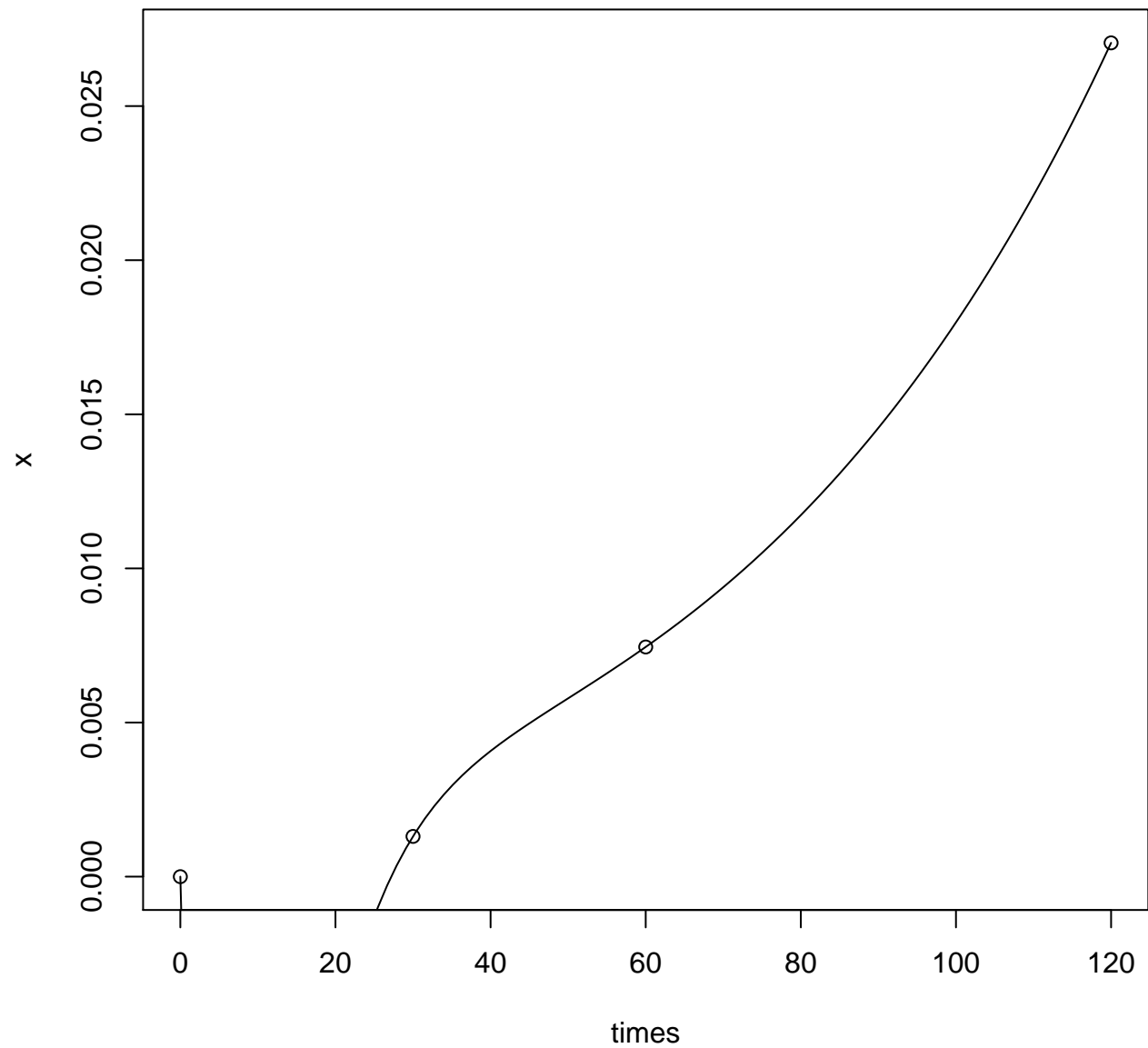

Supplement: Supplementary file 1 [file metabolites-10-00150-s001.zip › Supplementary Code S1/Glu-Pro/AL15/fit_input/res_fit_enr_AL15_pro_1.pdf]

## fitting results

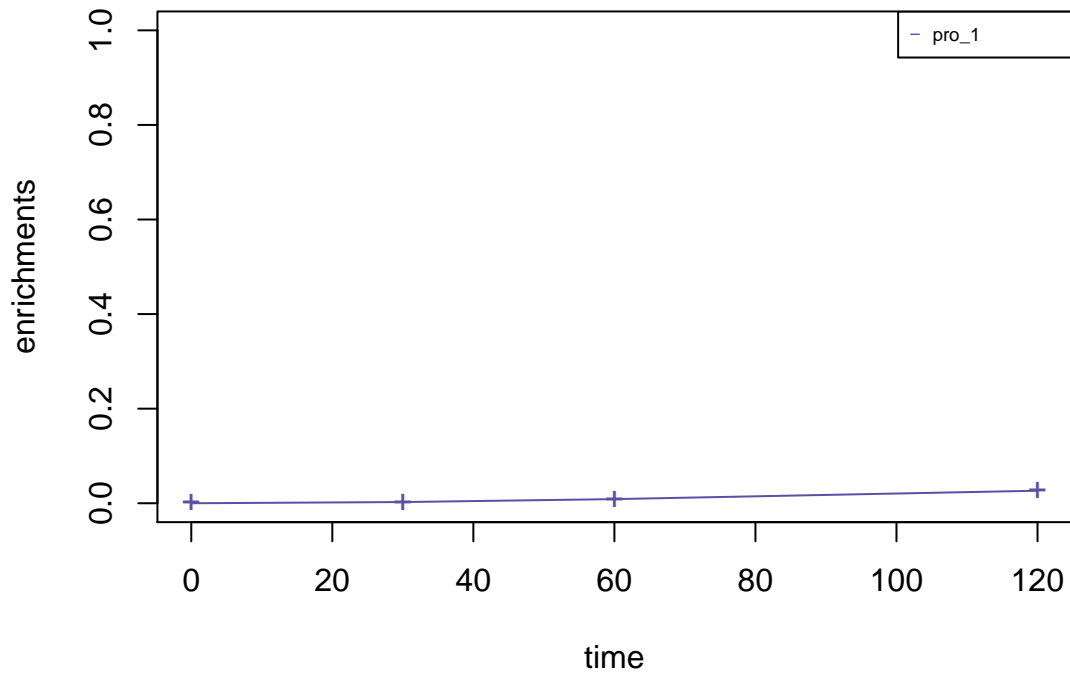

Supplement: Supplementary file 1 [file metabolites-10-00150-s001.zip › Supplementary Code S1/Glu-Pro/AL15/fit_subnet_fitpro/results.pdf]

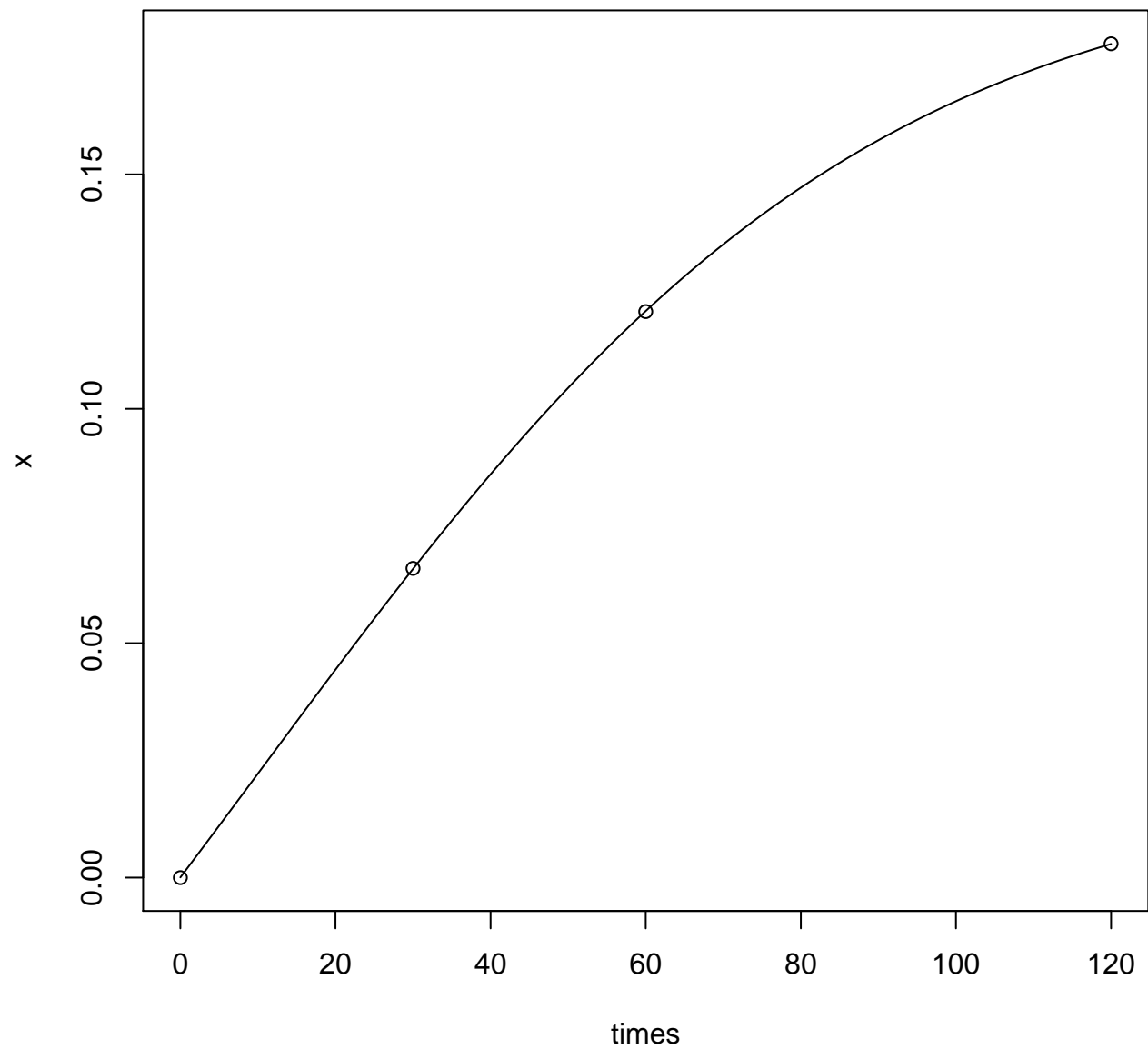

Supplement: Supplementary file 1 [file metabolites-10-00150-s001.zip › Supplementary Code S1/Glu-Pro/AL3/fit_input/res_fit_enr_AL3_glu_1.pdf]

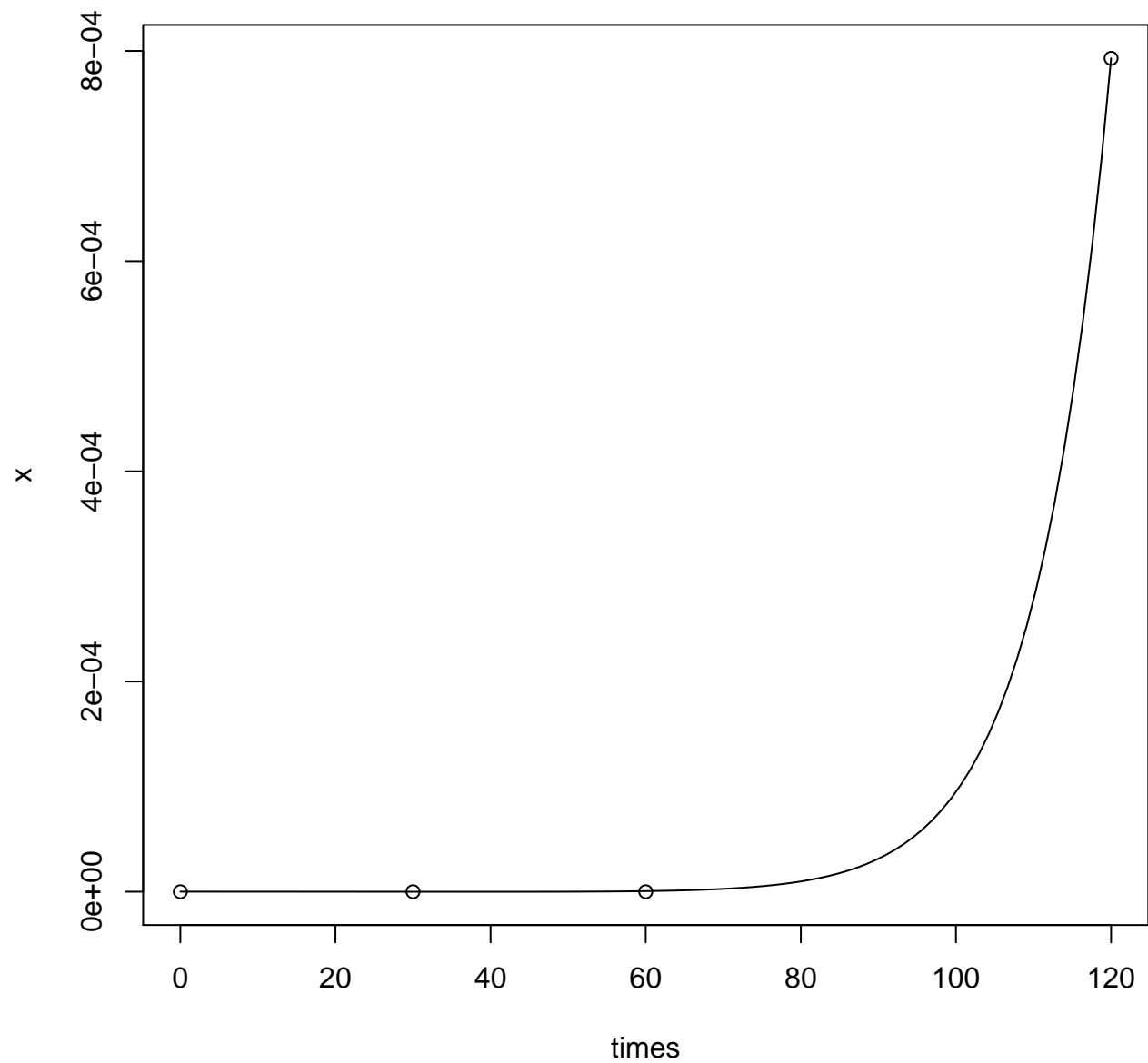

Supplement: Supplementary file 1 [file metabolites-10-00150-s001.zip › Supplementary Code S1/Glu-Pro/AL3/fit_input/res_fit_enr_AL3_pro_1.pdf]

## fitting results

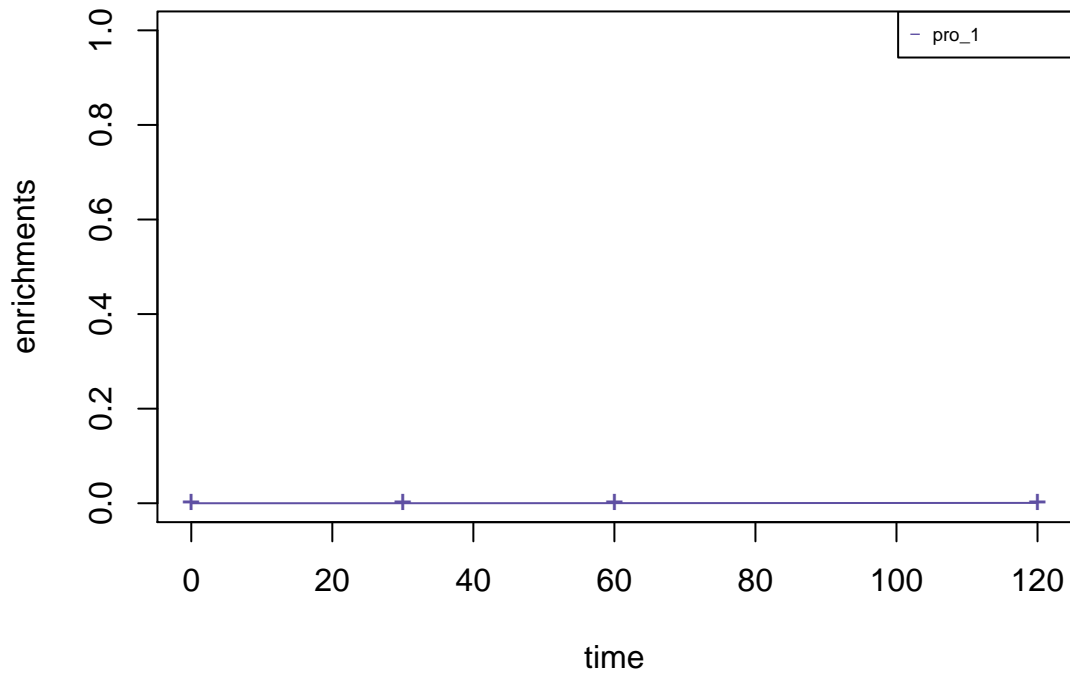

Supplement: Supplementary file 1 [file metabolites-10-00150-s001.zip › Supplementary Code S1/Glu-Pro/AL3/fit_subnet_fitpro/results.pdf]

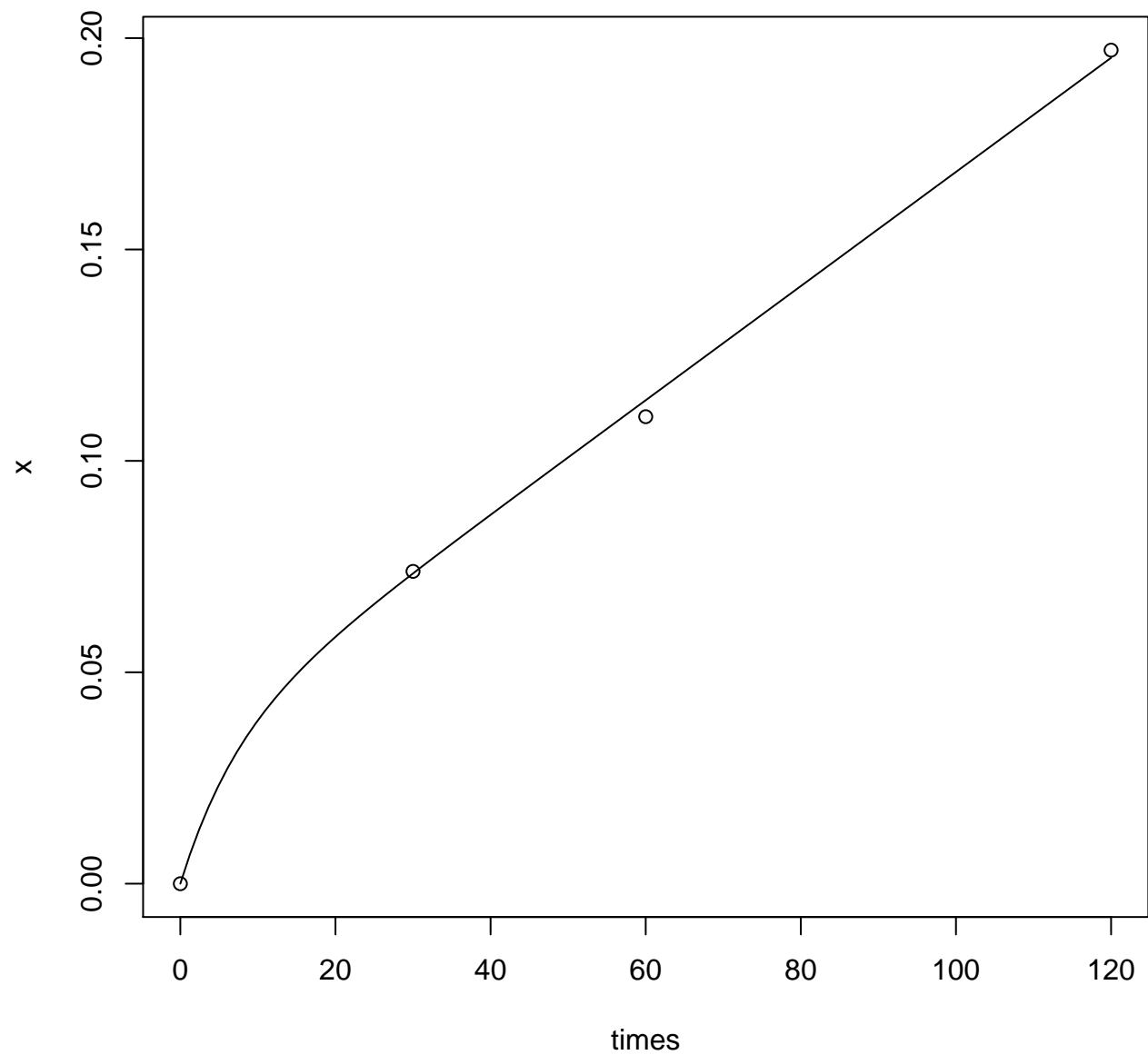

Supplement: Supplementary file 1 [file metabolites-10-00150-s001.zip › Supplementary Code S1/Glu-Pro/AL7/fit_input/res_fit_enr_AL7_glu_1.pdf]

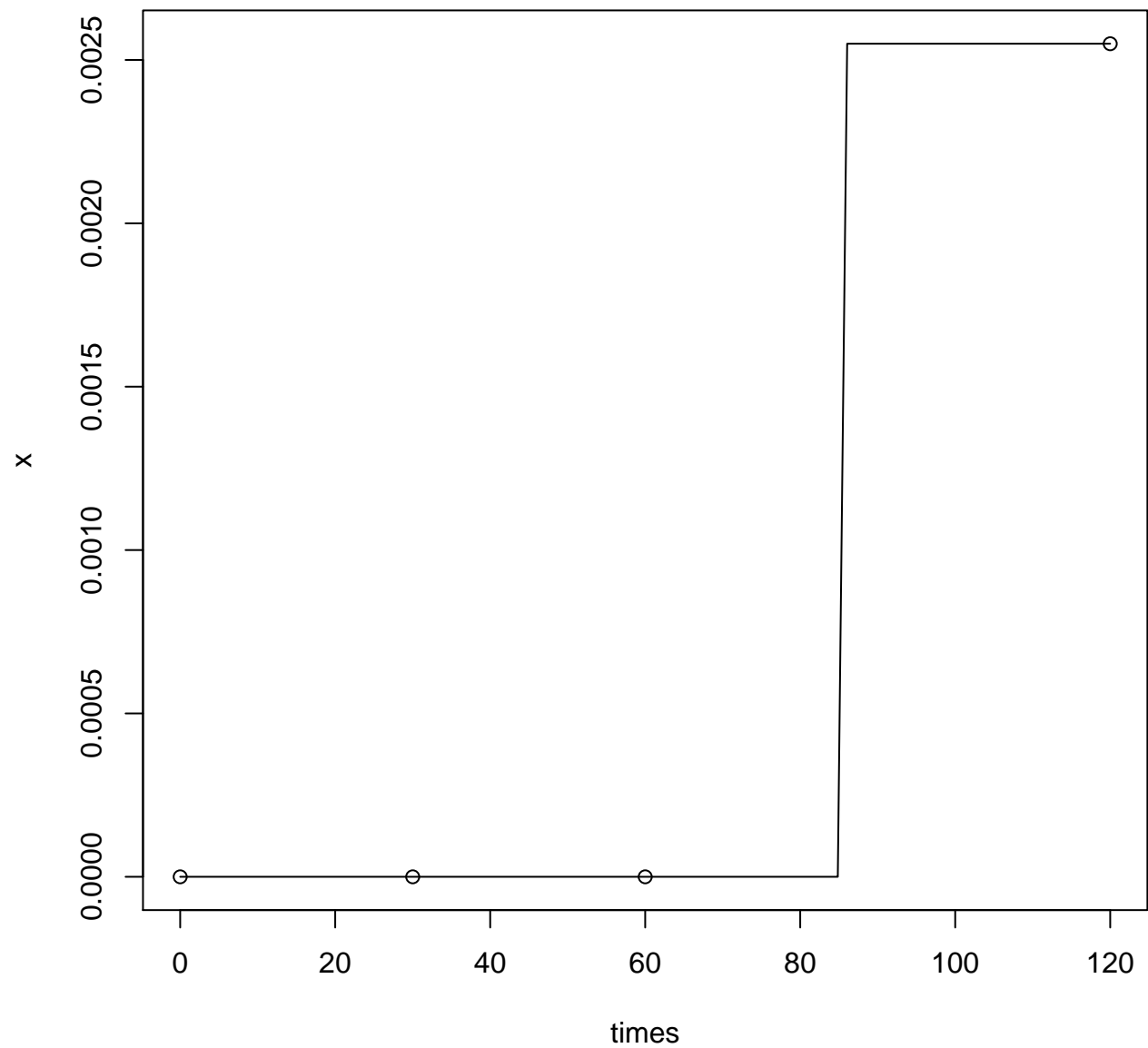

Supplement: Supplementary file 1 [file metabolites-10-00150-s001.zip › Supplementary Code S1/Glu-Pro/AL7/fit_input/res_fit_enr_AL7_pro_1.pdf]

## fitting results

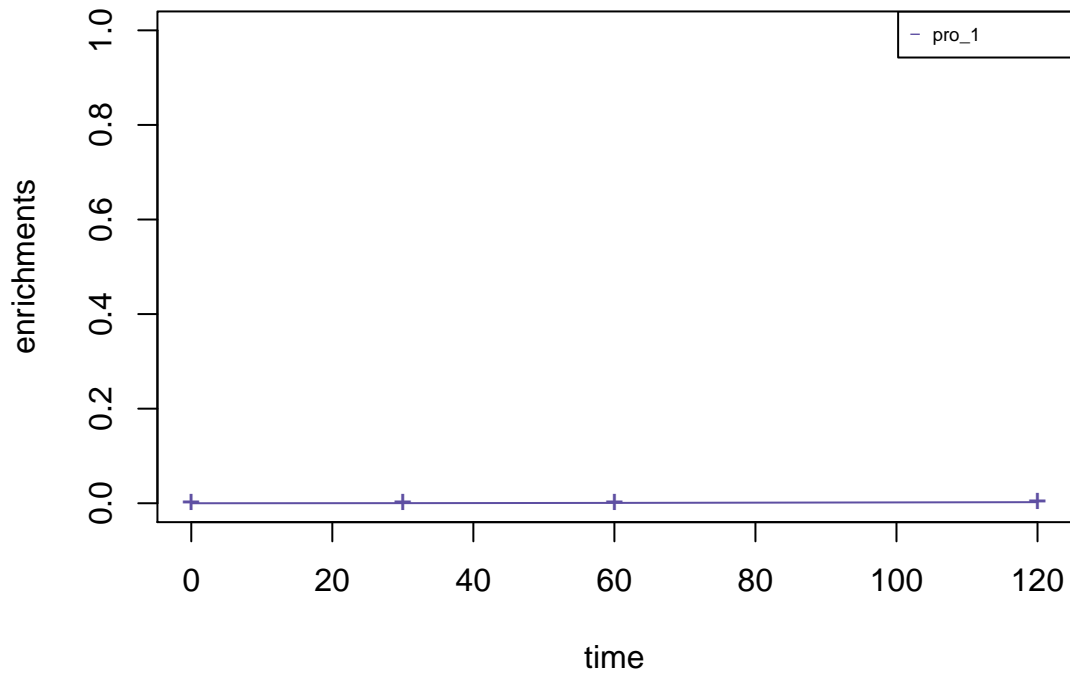

Supplement: Supplementary file 1 [file metabolites-10-00150-s001.zip › Supplementary Code S1/Glu-Pro/AL7/fit_subnet_fitpro/results.pdf]

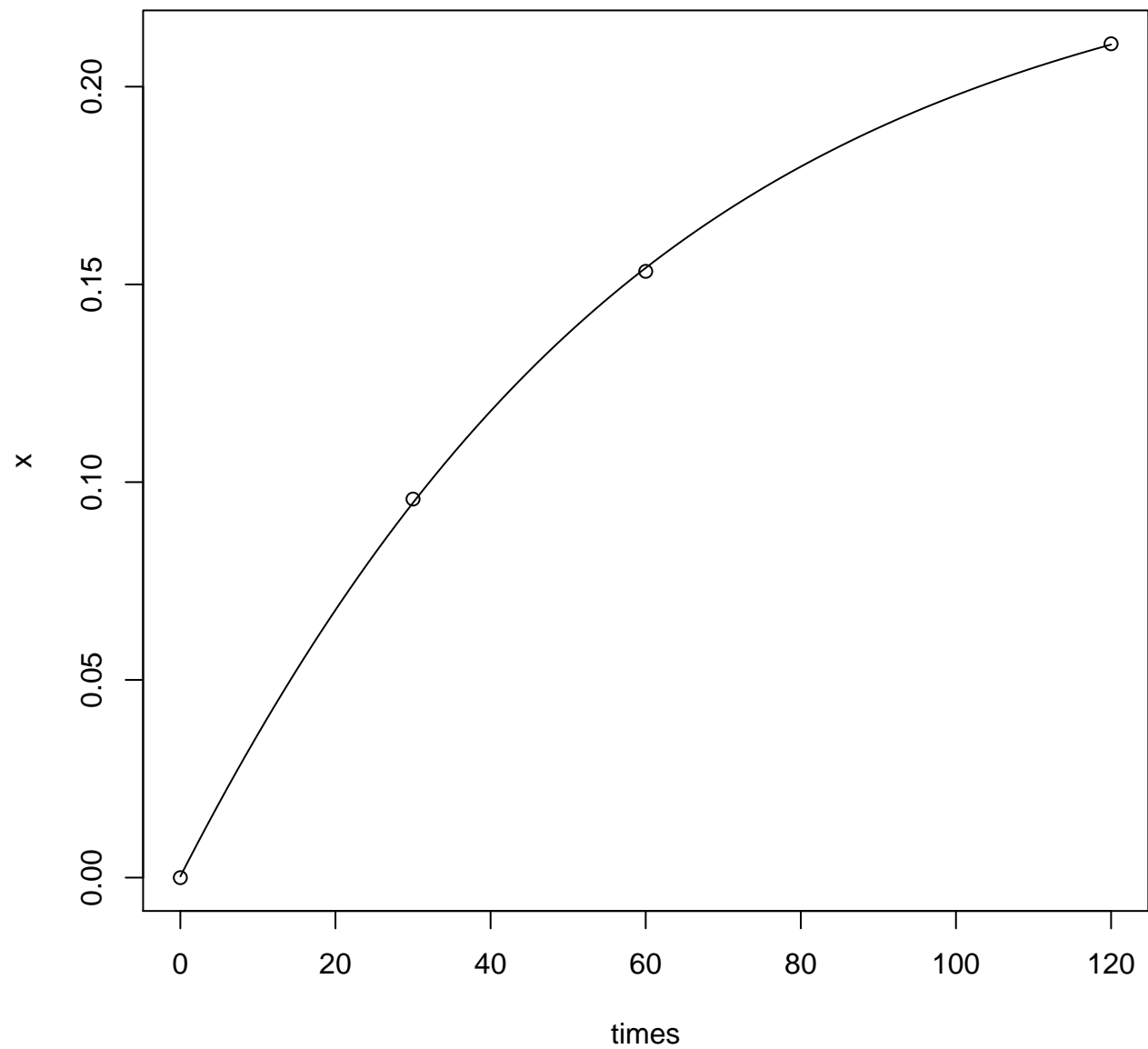

Supplement: Supplementary file 1 [file metabolites-10-00150-s001.zip › Supplementary Code S1/Glu-Pro/BL11/fit_input/res_fit_enr_BL11_glu_1.pdf]

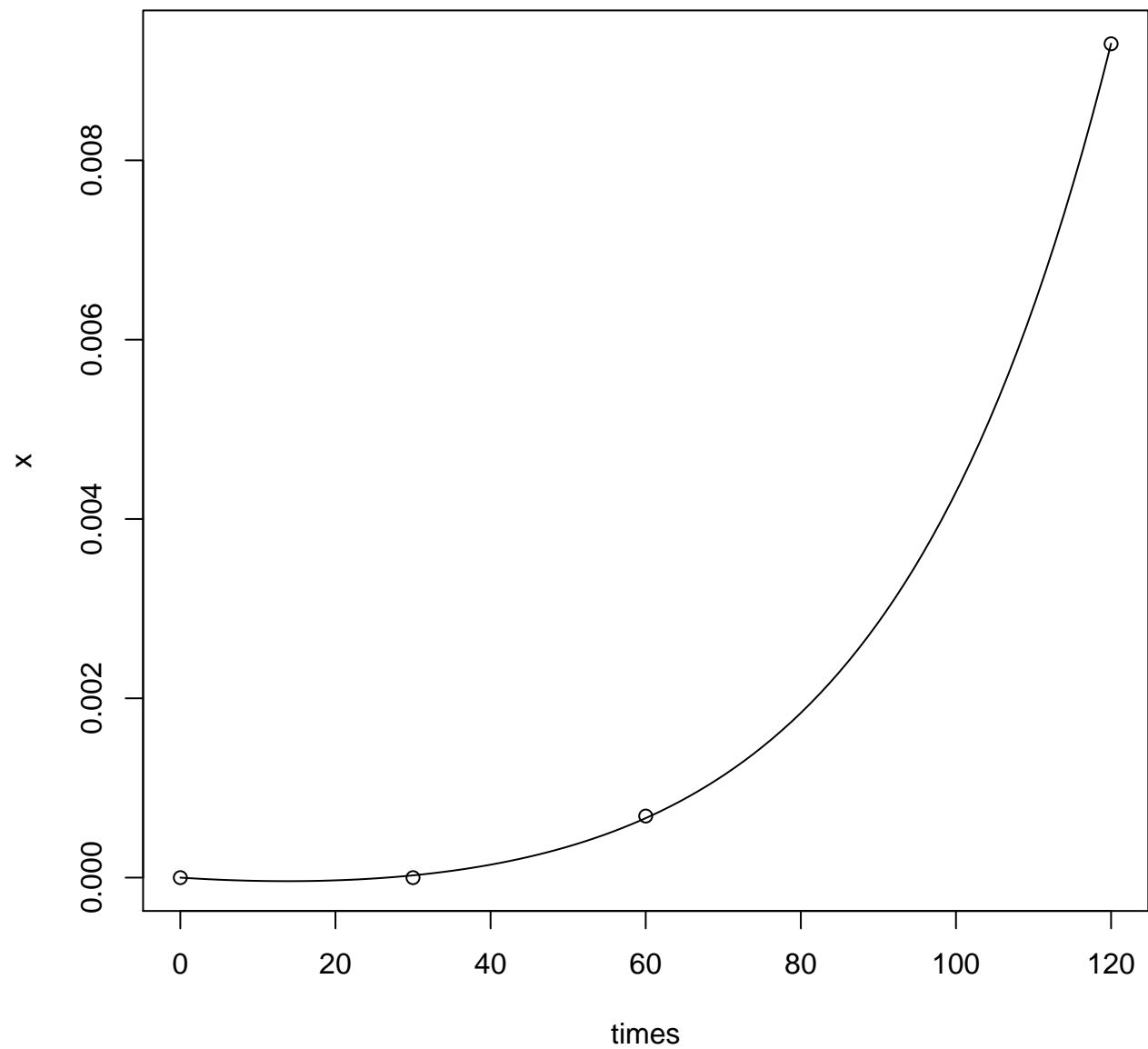

Supplement: Supplementary file 1 [file metabolites-10-00150-s001.zip › Supplementary Code S1/Glu-Pro/BL11/fit_input/res_fit_enr_BL11_pro_1.pdf]

## fitting results

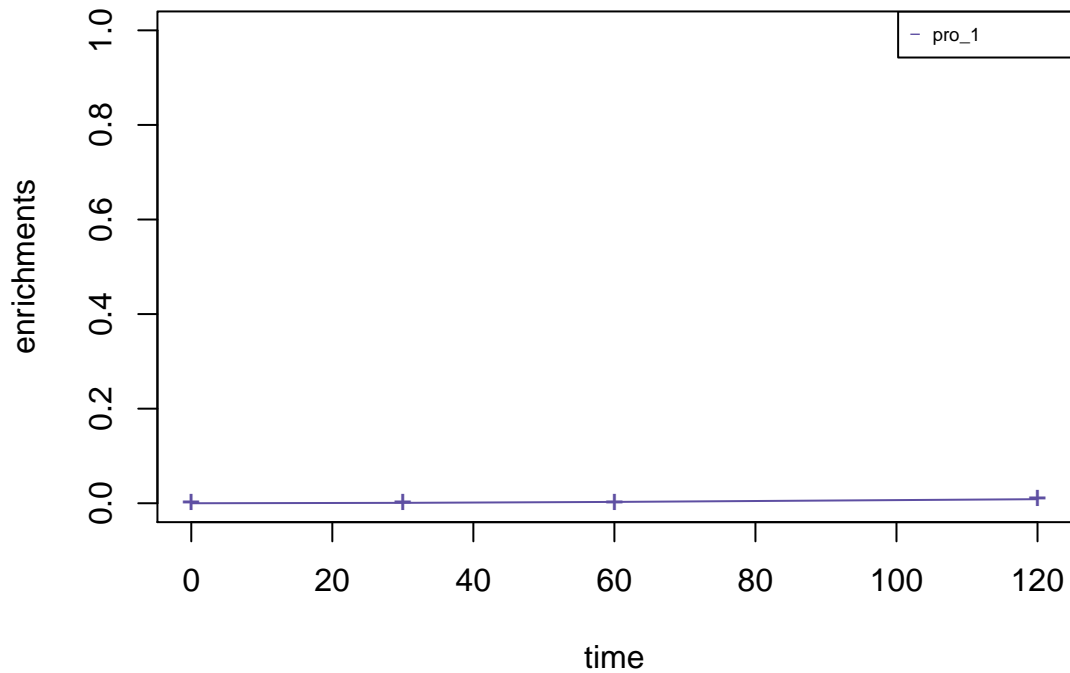

Supplement: Supplementary file 1 [file metabolites-10-00150-s001.zip › Supplementary Code S1/Glu-Pro/BL11/fit_subnet_fitpro/results.pdf]

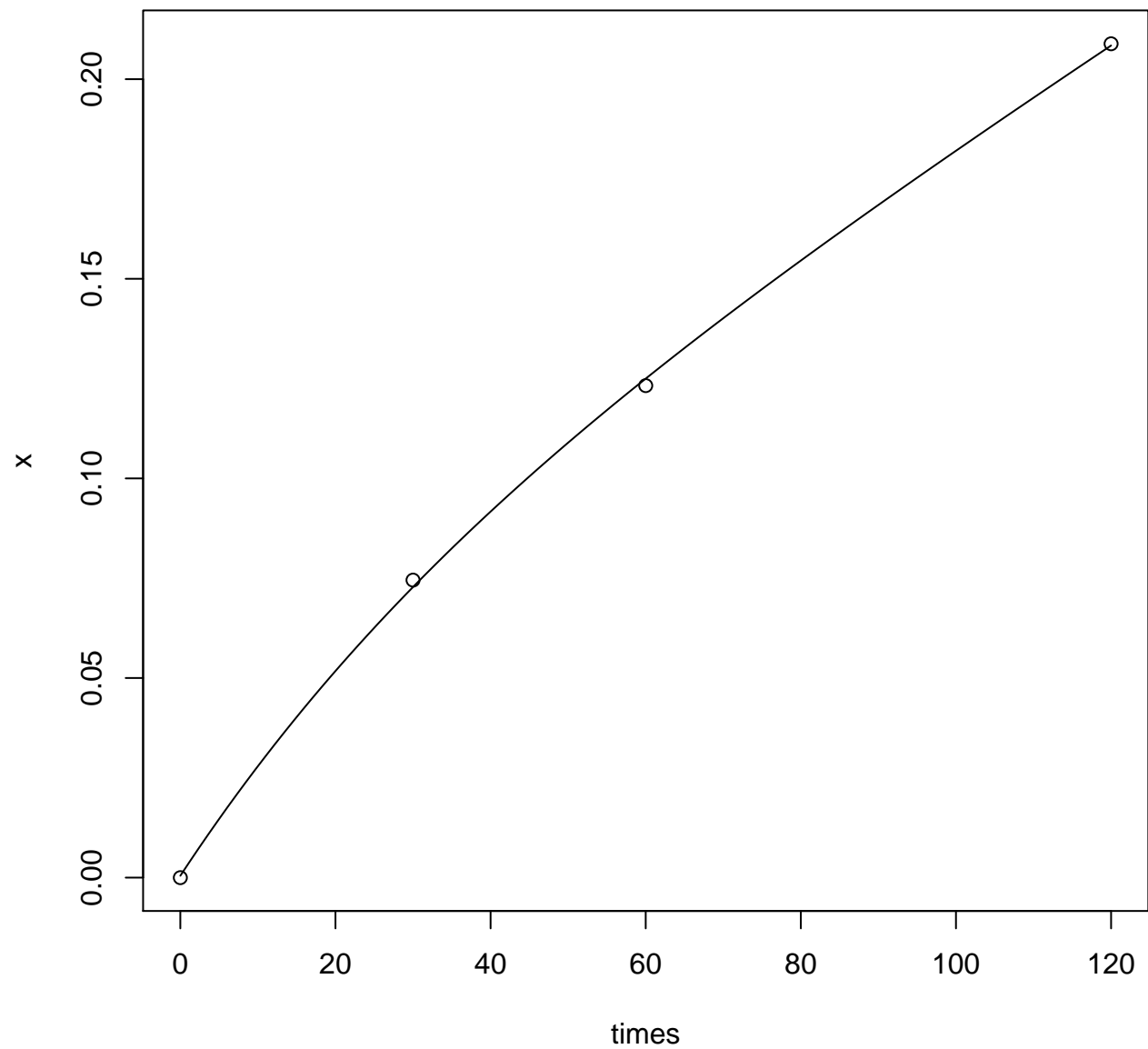

Supplement: Supplementary file 1 [file metabolites-10-00150-s001.zip › Supplementary Code S1/Glu-Pro/BL15/fit_input/res_fit_enr_BL15_glu_1.pdf]

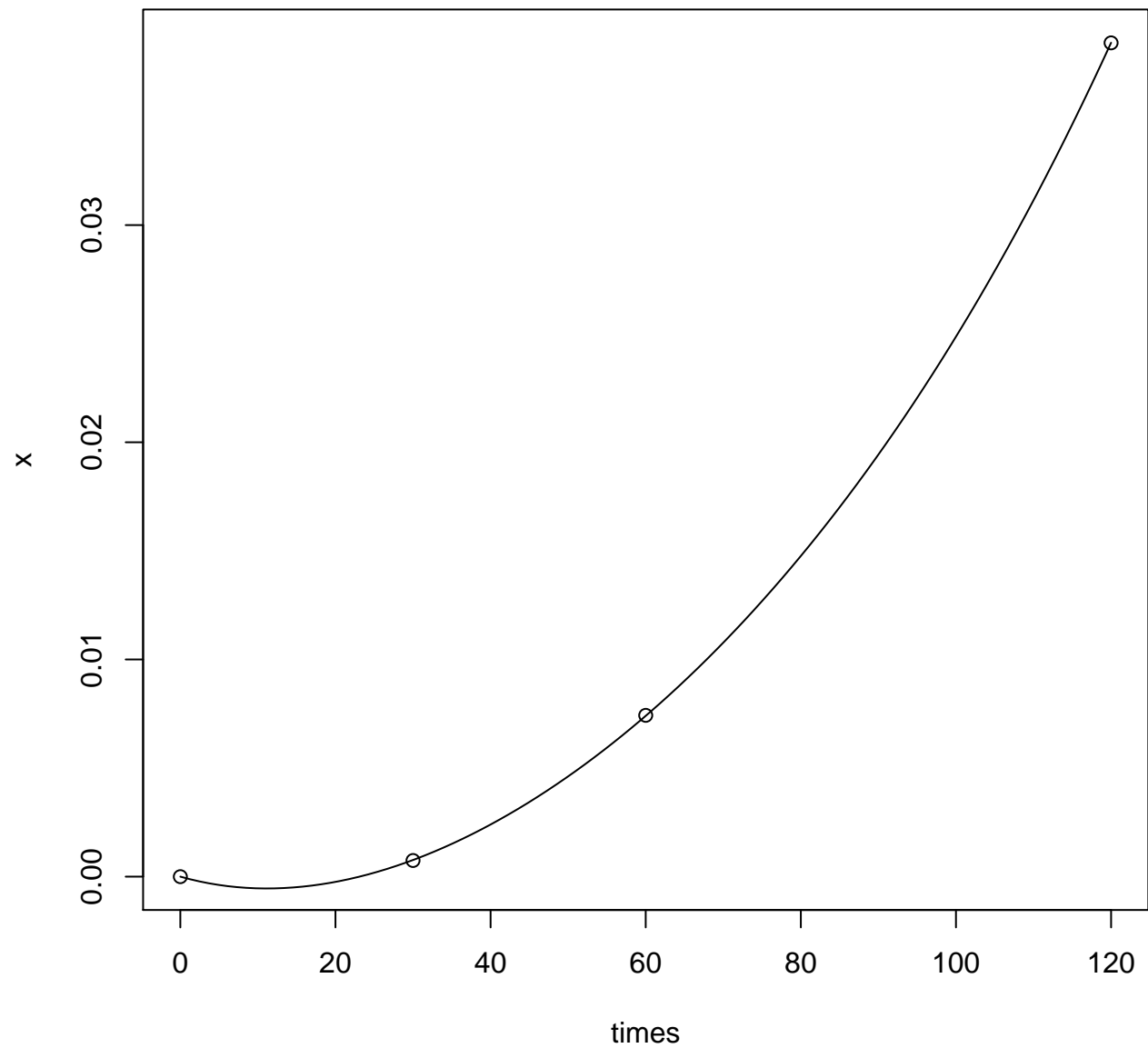

Supplement: Supplementary file 1 [file metabolites-10-00150-s001.zip › Supplementary Code S1/Glu-Pro/BL15/fit_input/res_fit_enr_BL15_pro_1.pdf]

## fitting results

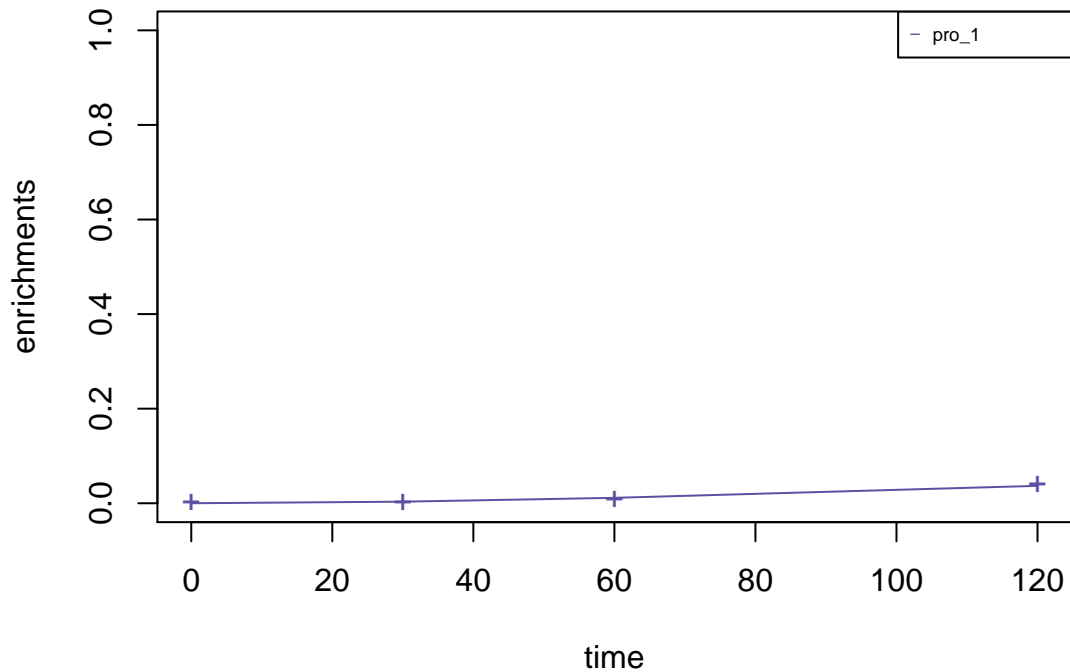

Supplement: Supplementary file 1 [file metabolites-10-00150-s001.zip › Supplementary Code S1/Glu-Pro/BL15/fit_subnet_fitpro/results.pdf]

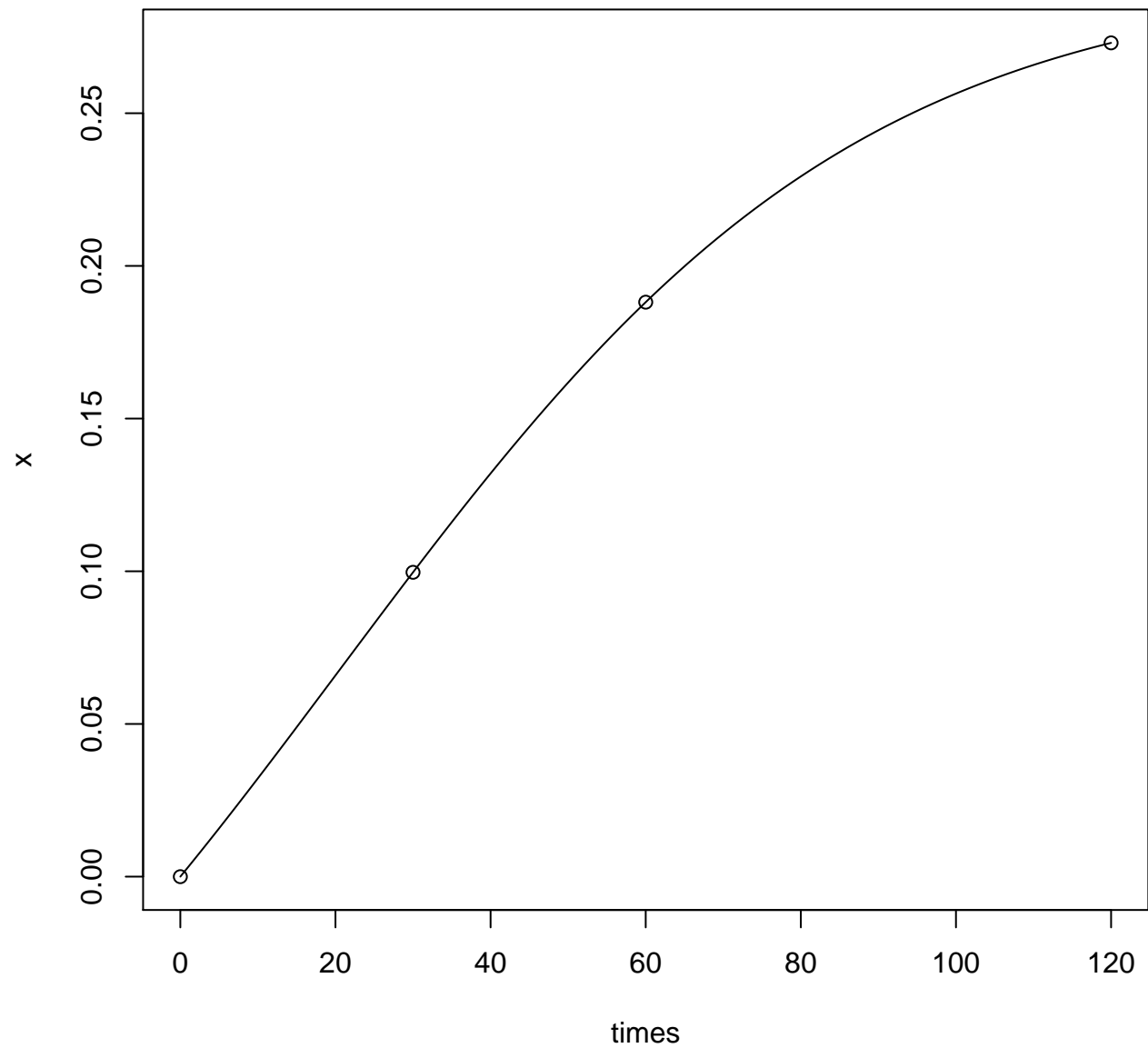

Supplement: Supplementary file 1 [file metabolites-10-00150-s001.zip › Supplementary Code S1/Glu-Pro/BL3/fit_input/res_fit_enr_BL3_glu_1.pdf]

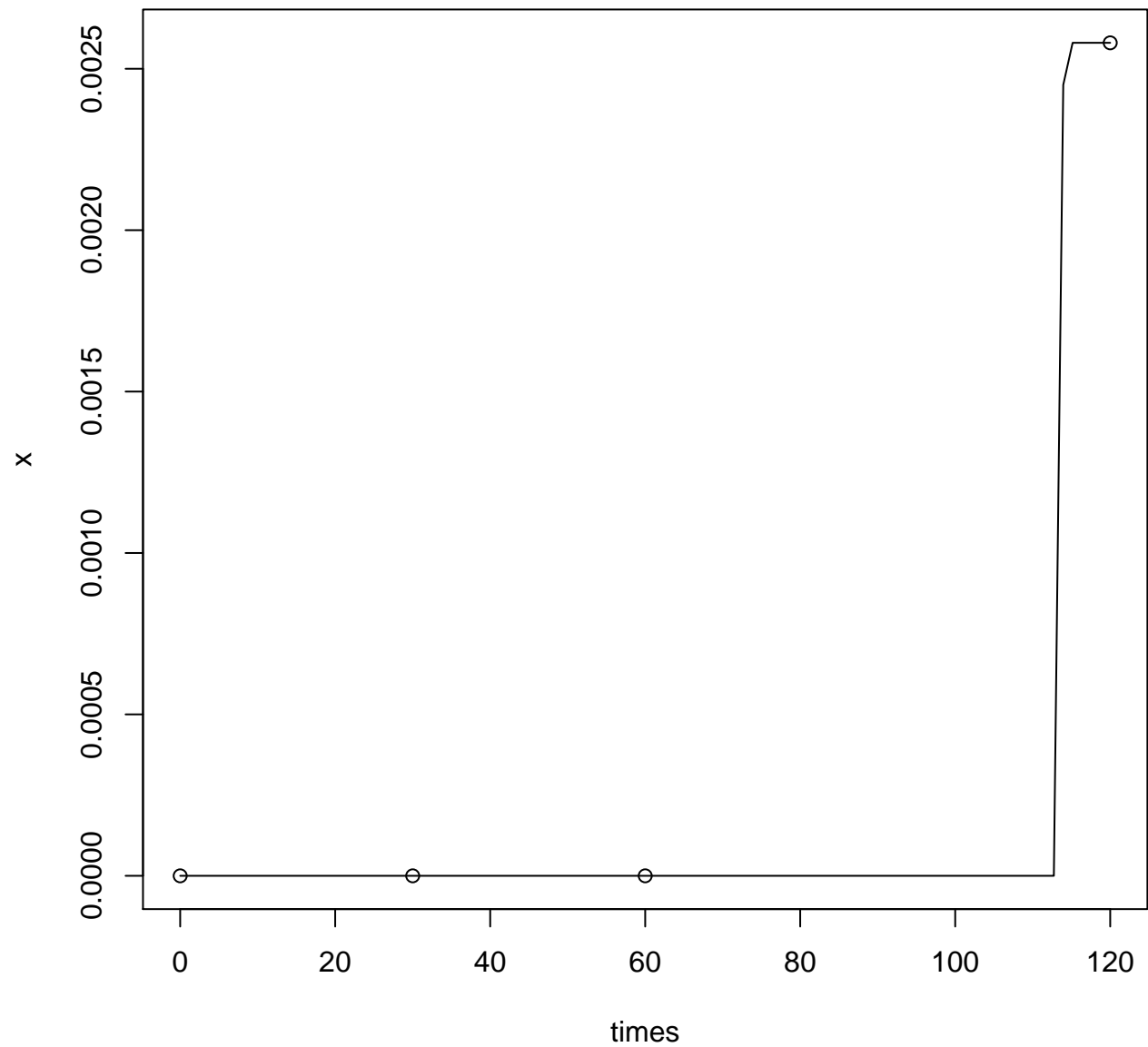

Supplement: Supplementary file 1 [file metabolites-10-00150-s001.zip › Supplementary Code S1/Glu-Pro/BL3/fit_input/res_fit_enr_BL3_pro_1.pdf]

## fitting results

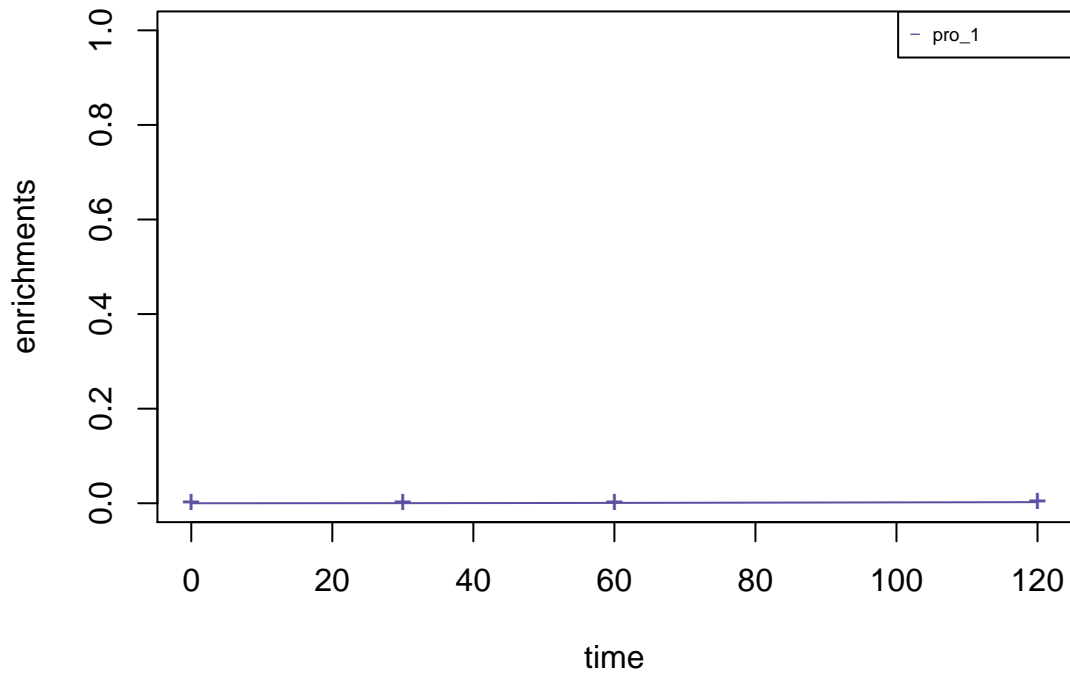

Supplement: Supplementary file 1 [file metabolites-10-00150-s001.zip › Supplementary Code S1/Glu-Pro/BL3/fit_subnet_fitpro/results.pdf]

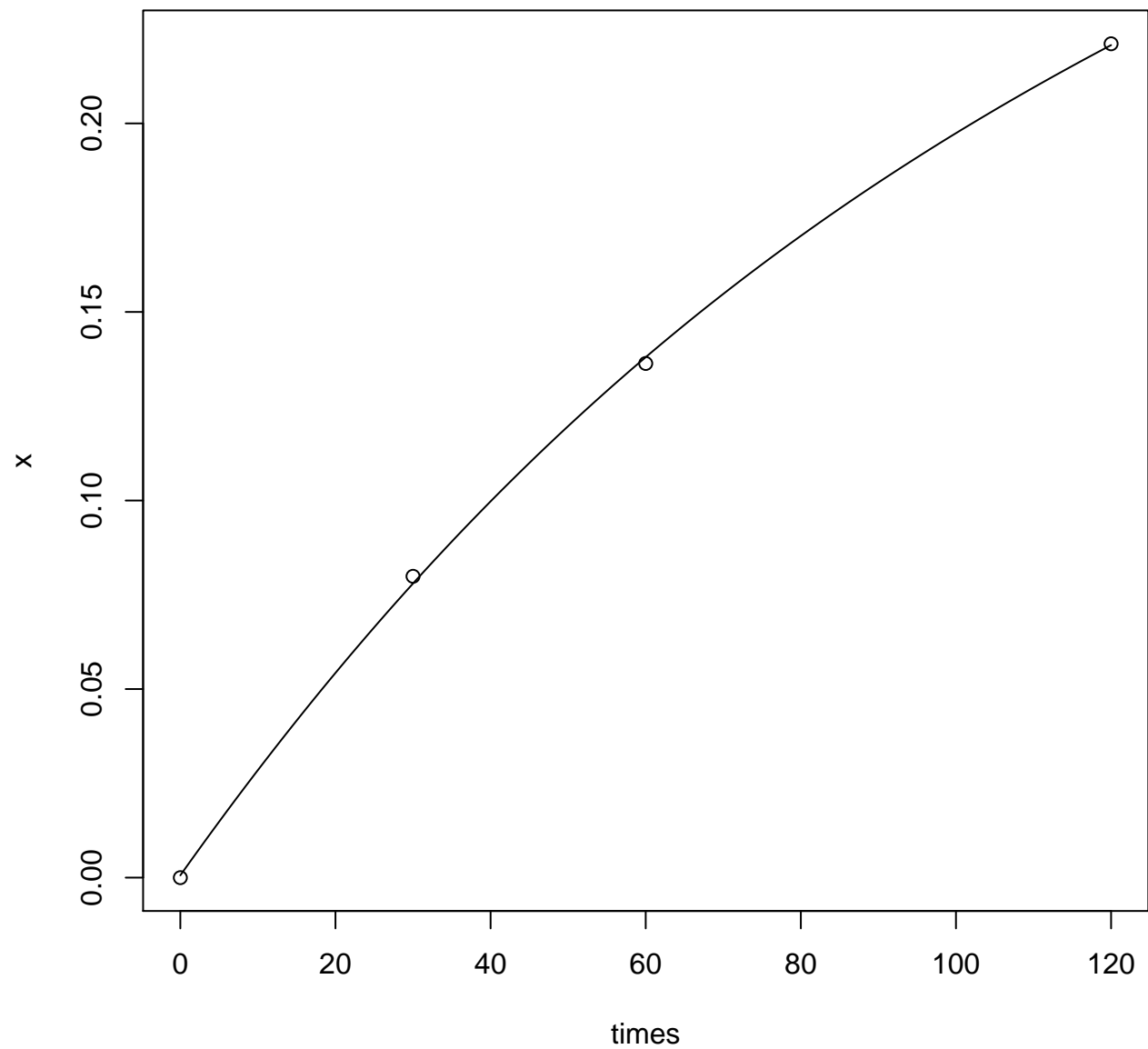

Supplement: Supplementary file 1 [file metabolites-10-00150-s001.zip › Supplementary Code S1/Glu-Pro/BL7/fit_input/res_fit_enr_BL7_glu_1.pdf]

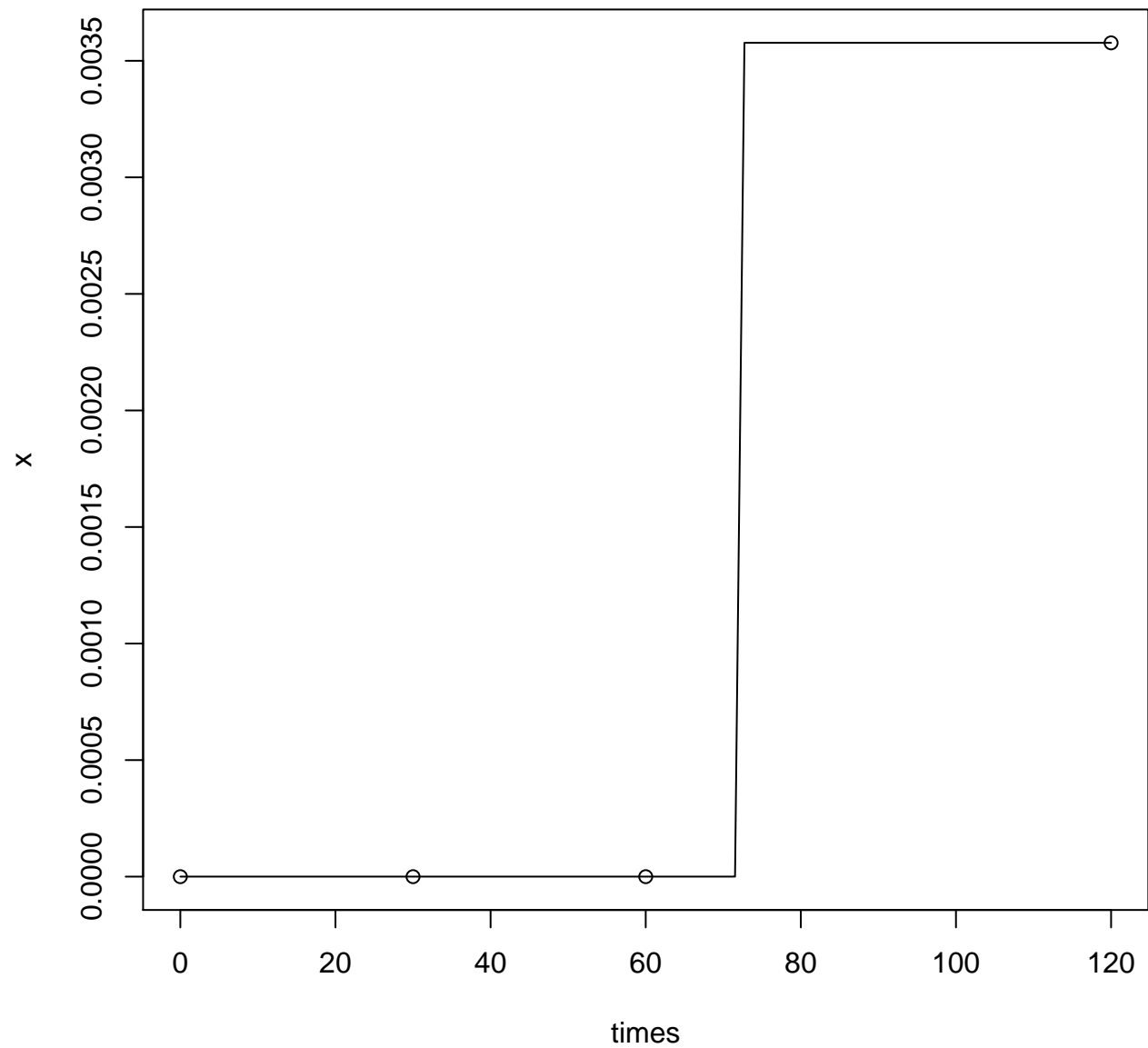

Supplement: Supplementary file 1 [file metabolites-10-00150-s001.zip › Supplementary Code S1/Glu-Pro/BL7/fit_input/res_fit_enr_BL7_pro_1.pdf]

## fitting results

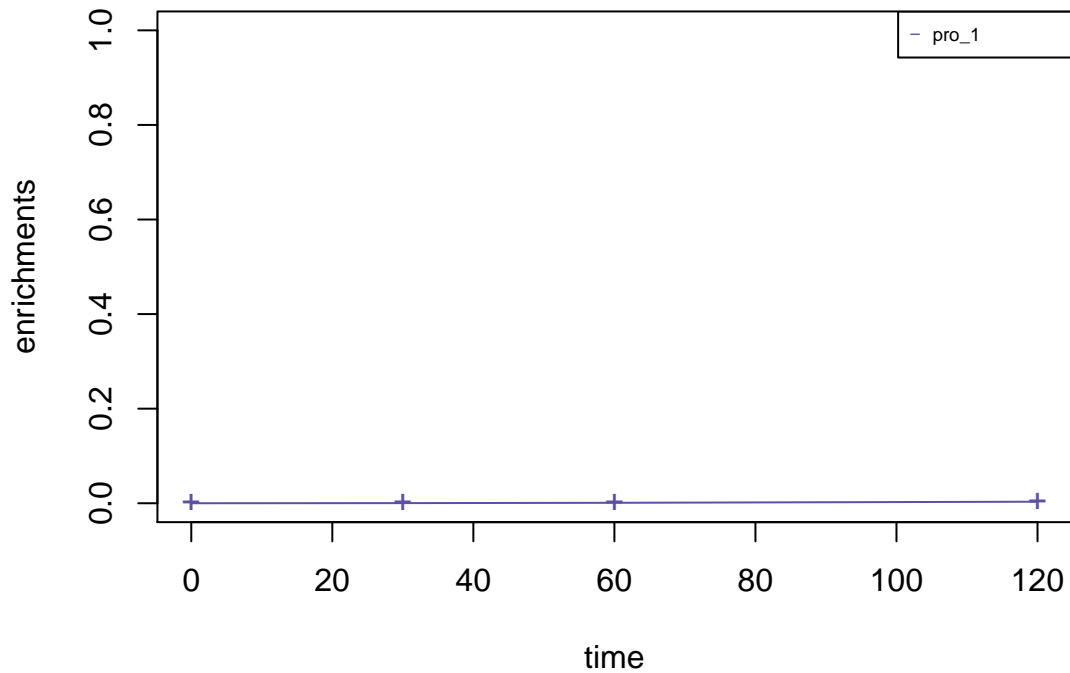

Supplement: Supplementary file 1 [file metabolites-10-00150-s001.zip › Supplementary Code S1/Glu-Pro/BL7/fit_subnet_fitpro/results.pdf]

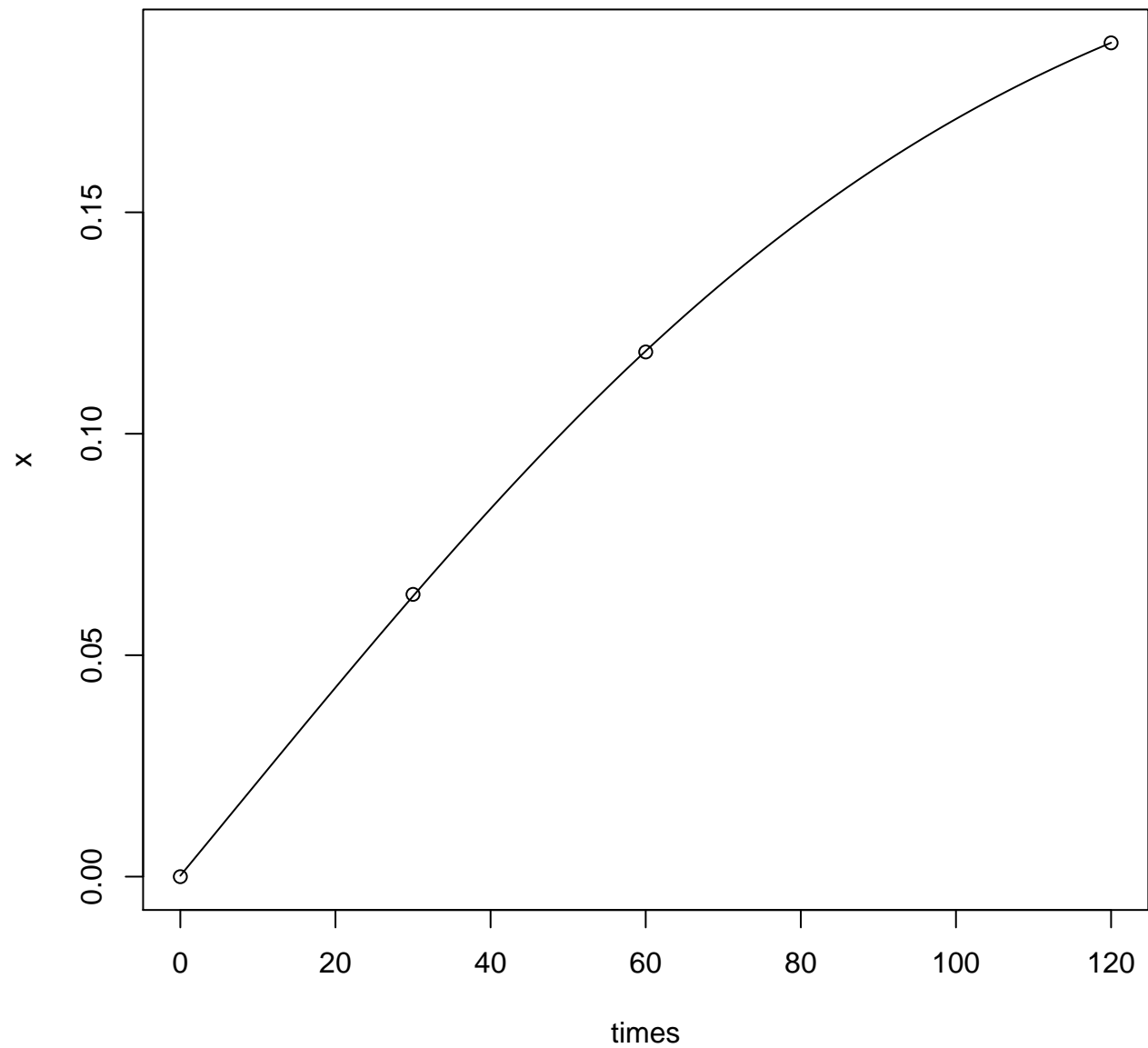

Supplement: Supplementary file 1 [file metabolites-10-00150-s001.zip › Supplementary Code S1/Glu-Pro/CL11/fit_input/res_fit_enr_CL11_glu_1.pdf]

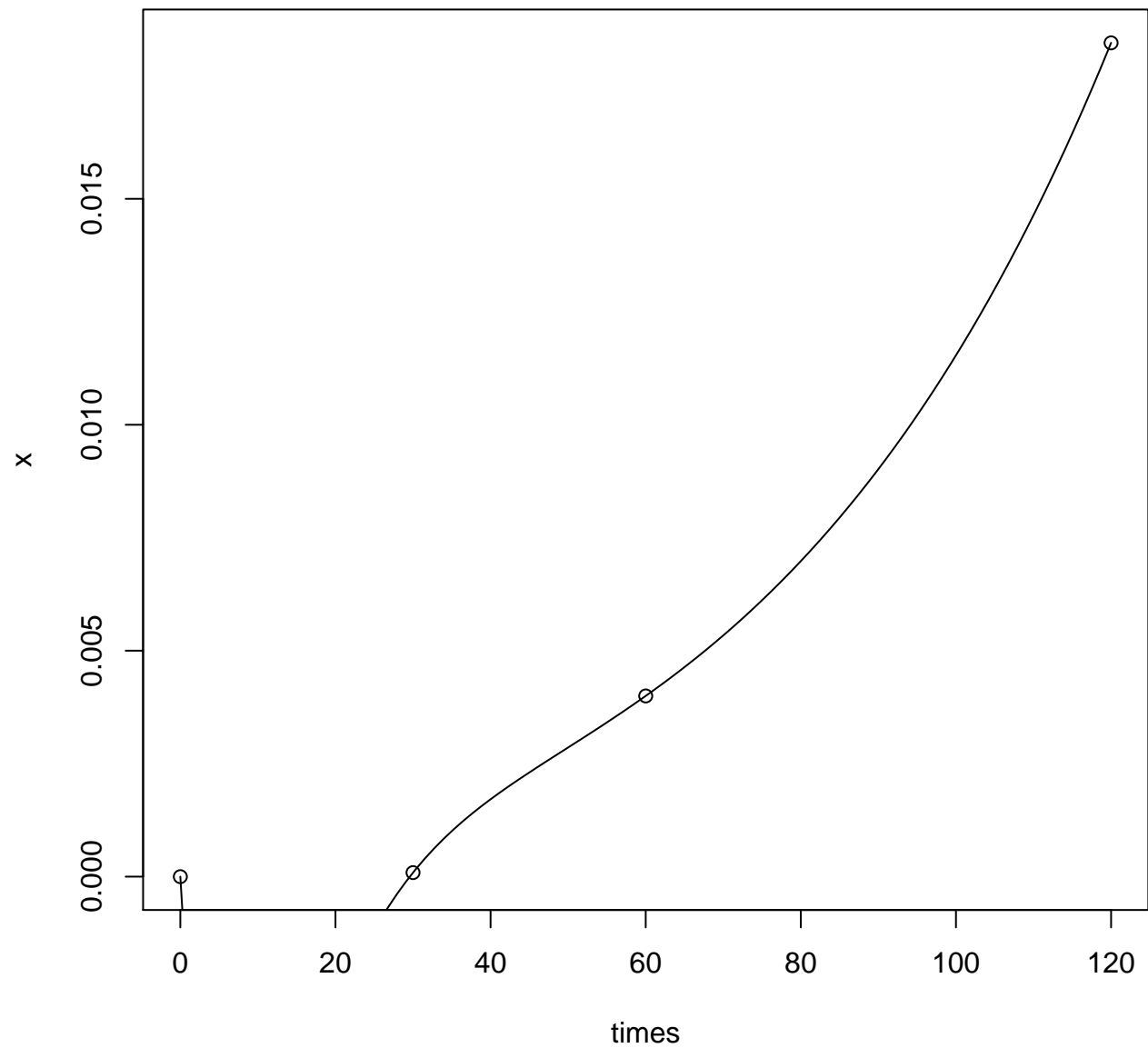

Supplement: Supplementary file 1 [file metabolites-10-00150-s001.zip › Supplementary Code S1/Glu-Pro/CL11/fit_input/res_fit_enr_CL11_pro_1.pdf]

## fitting results

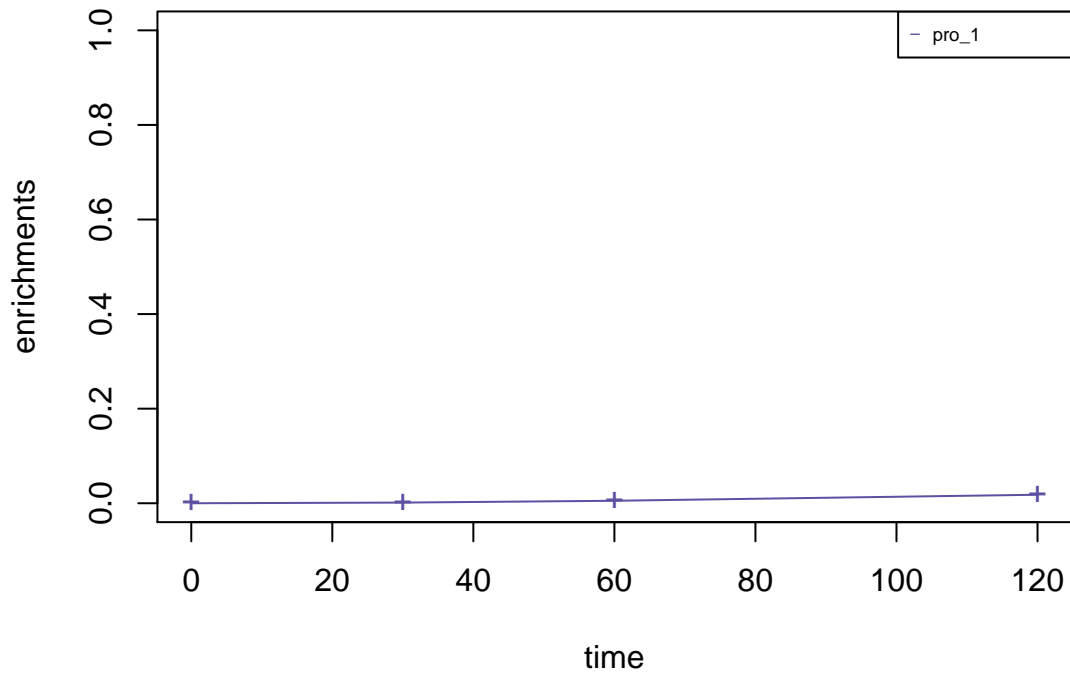

Supplement: Supplementary file 1 [file metabolites-10-00150-s001.zip › Supplementary Code S1/Glu-Pro/CL11/fit_subnet_fitpro/results.pdf]

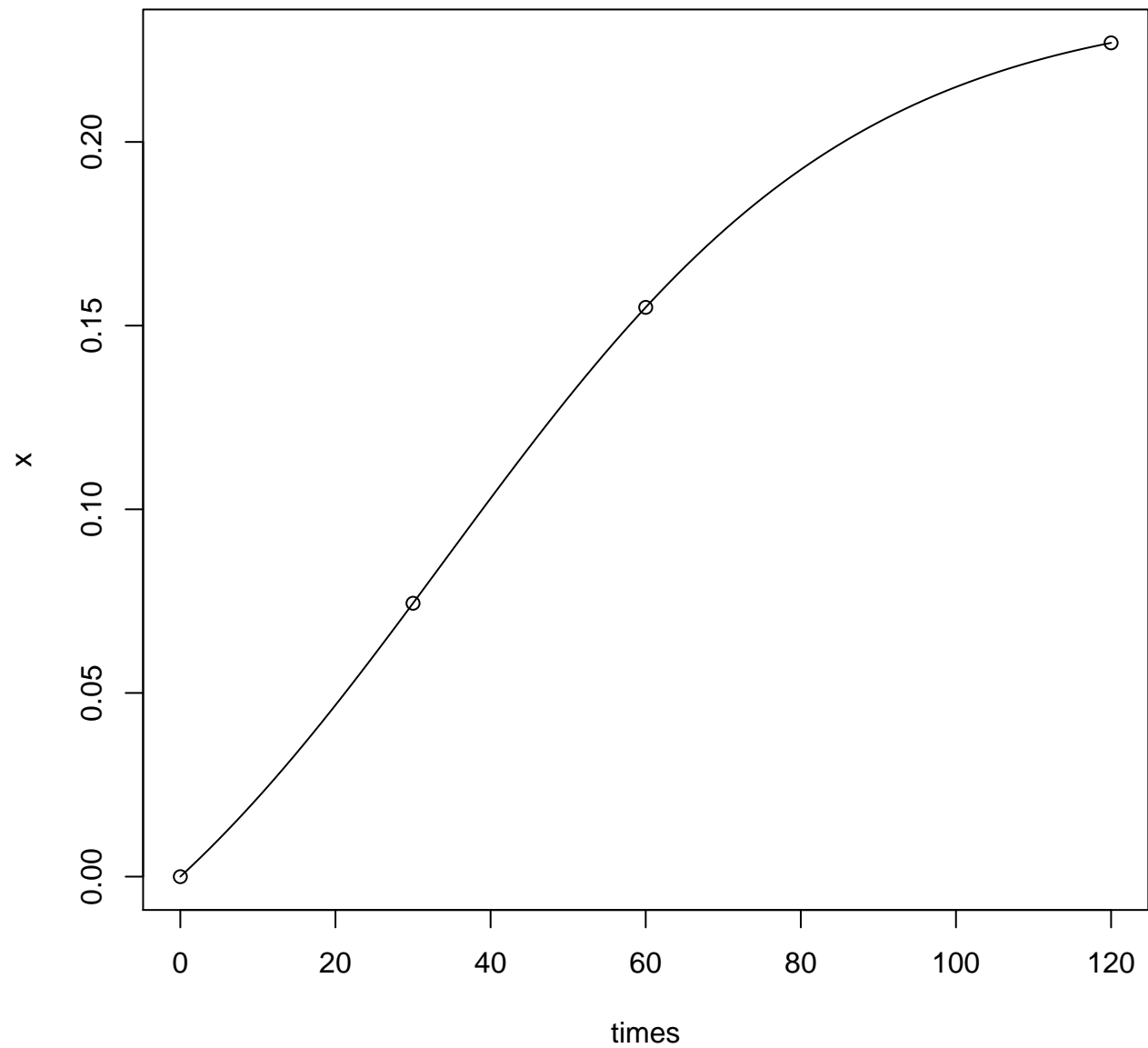

Supplement: Supplementary file 1 [file metabolites-10-00150-s001.zip › Supplementary Code S1/Glu-Pro/CL15/fit_input/res_fit_enr_CL15_glu_1.pdf]

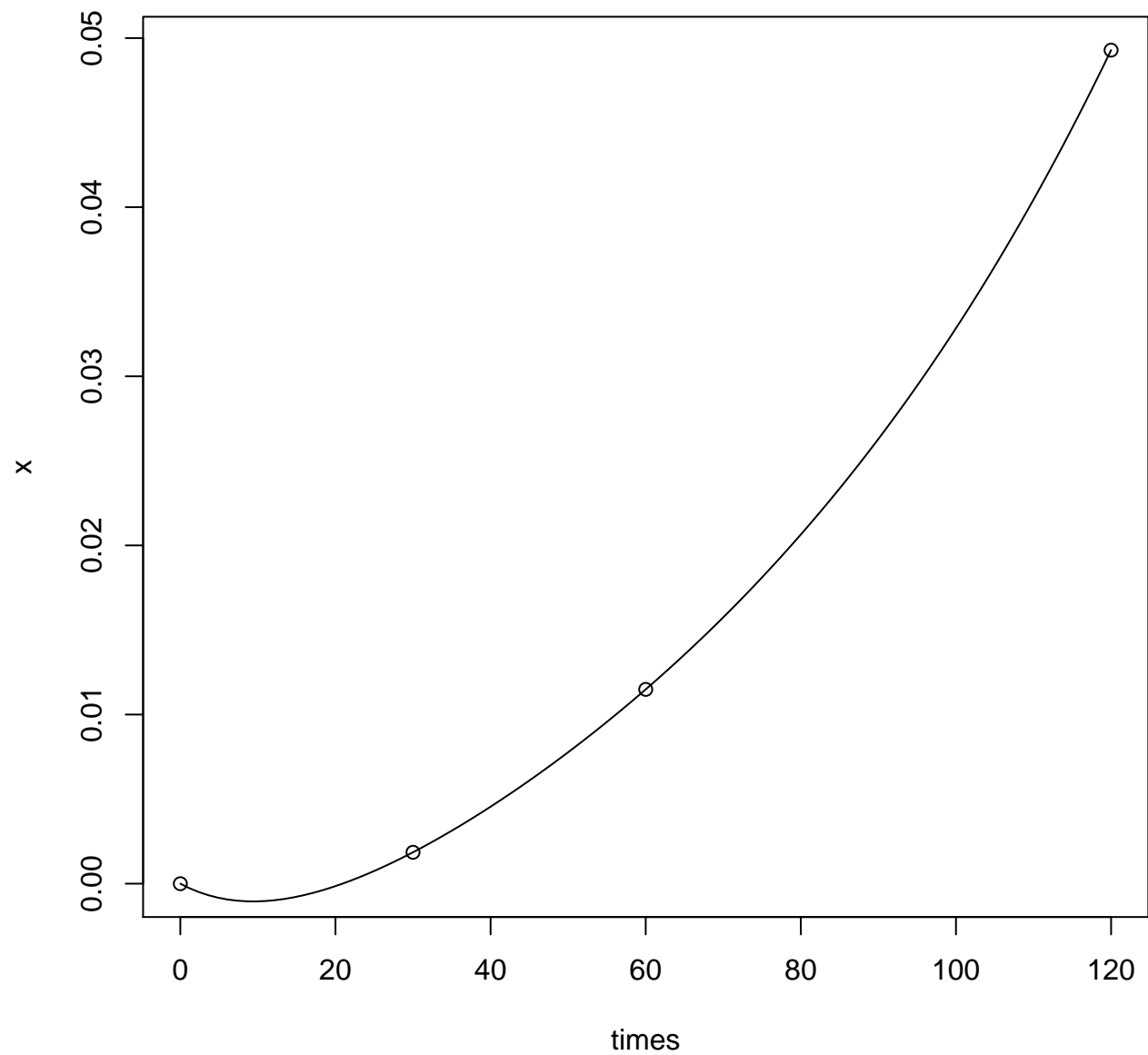

Supplement: Supplementary file 1 [file metabolites-10-00150-s001.zip › Supplementary Code S1/Glu-Pro/CL15/fit_input/res_fit_enr_CL15_pro_1.pdf]

## fitting results

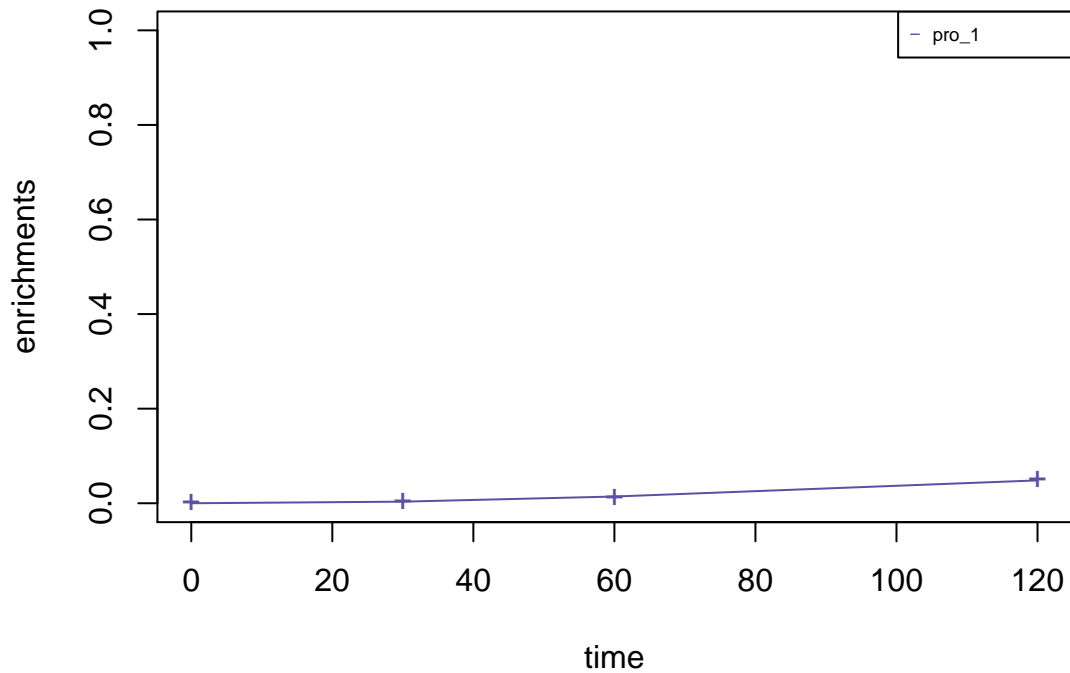

Supplement: Supplementary file 1 [file metabolites-10-00150-s001.zip › Supplementary Code S1/Glu-Pro/CL15/fit_subnet_fitpro/results.pdf]

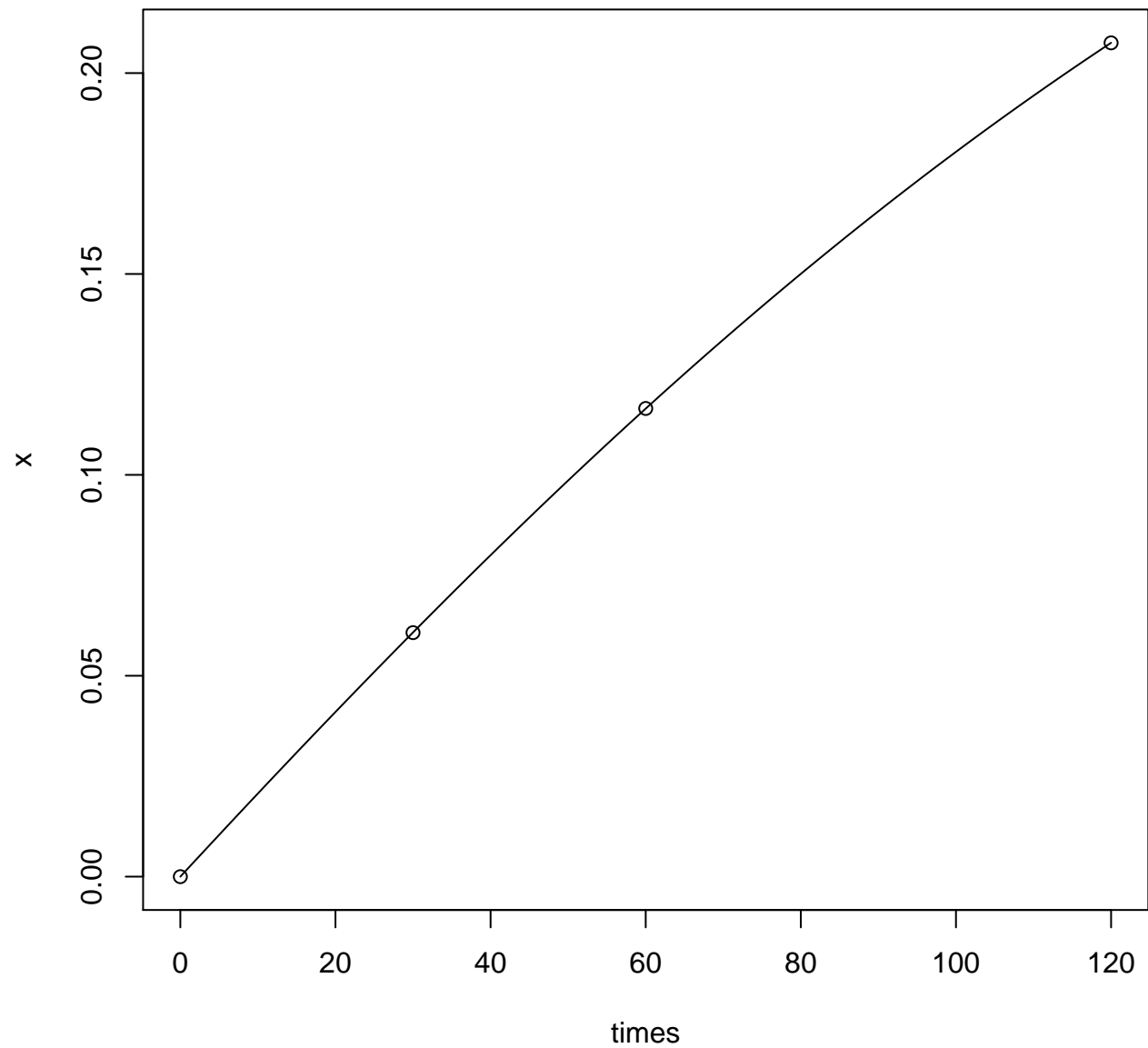

Supplement: Supplementary file 1 [file metabolites-10-00150-s001.zip › Supplementary Code S1/Glu-Pro/CL3/fit_input/res_fit_enr_CL3_glu_1.pdf]

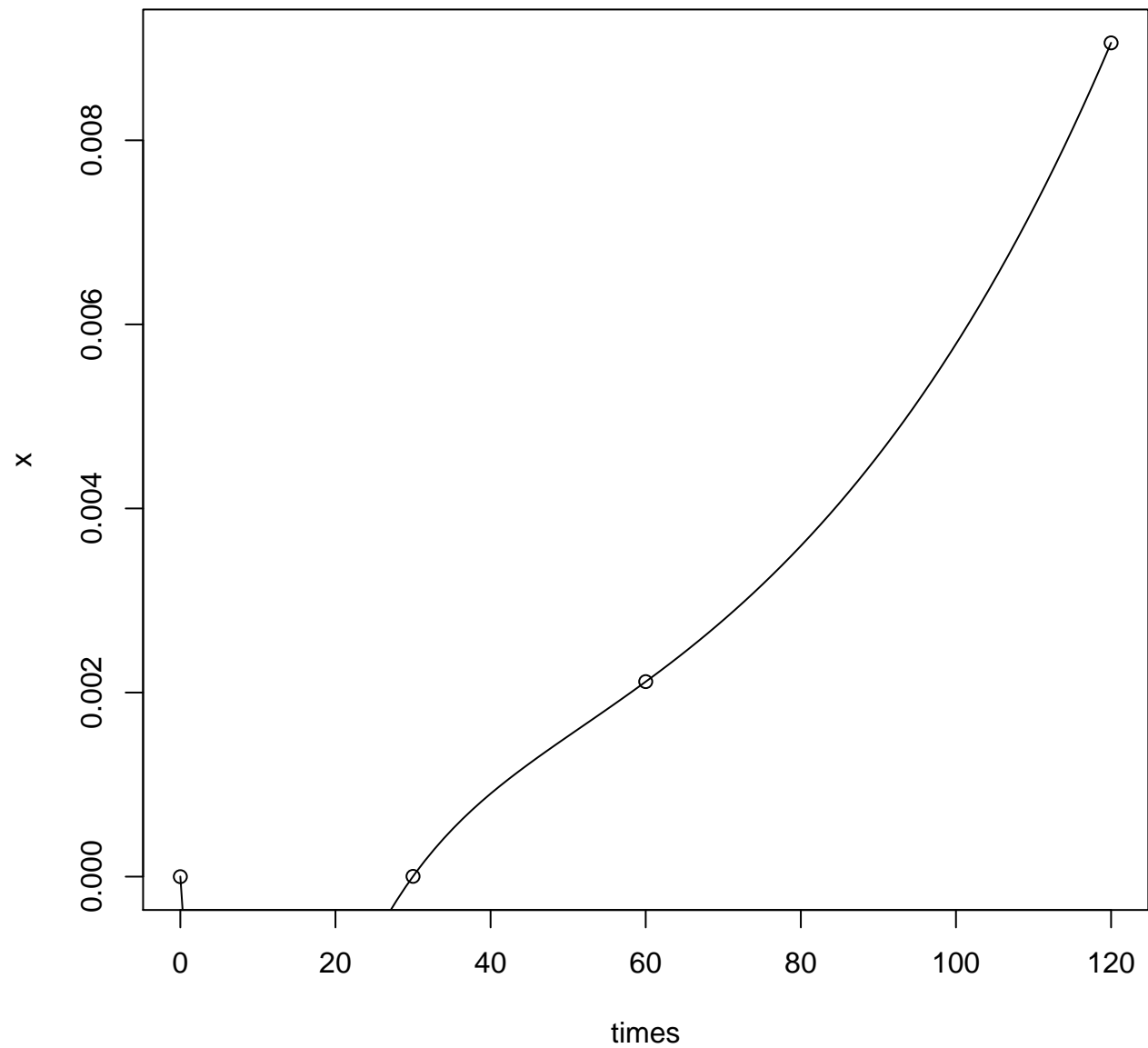

Supplement: Supplementary file 1 [file metabolites-10-00150-s001.zip › Supplementary Code S1/Glu-Pro/CL3/fit_input/res_fit_enr_CL3_pro_1.pdf]

## fitting results

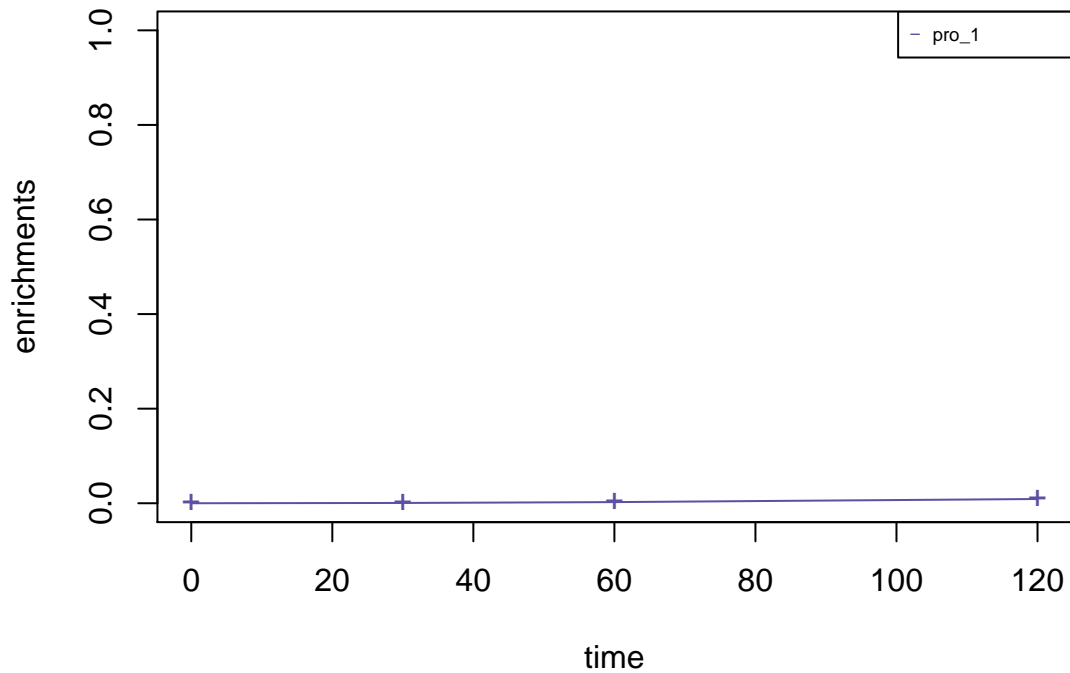

Supplement: Supplementary file 1 [file metabolites-10-00150-s001.zip › Supplementary Code S1/Glu-Pro/CL3/fit_subnet_fitpro/results.pdf]

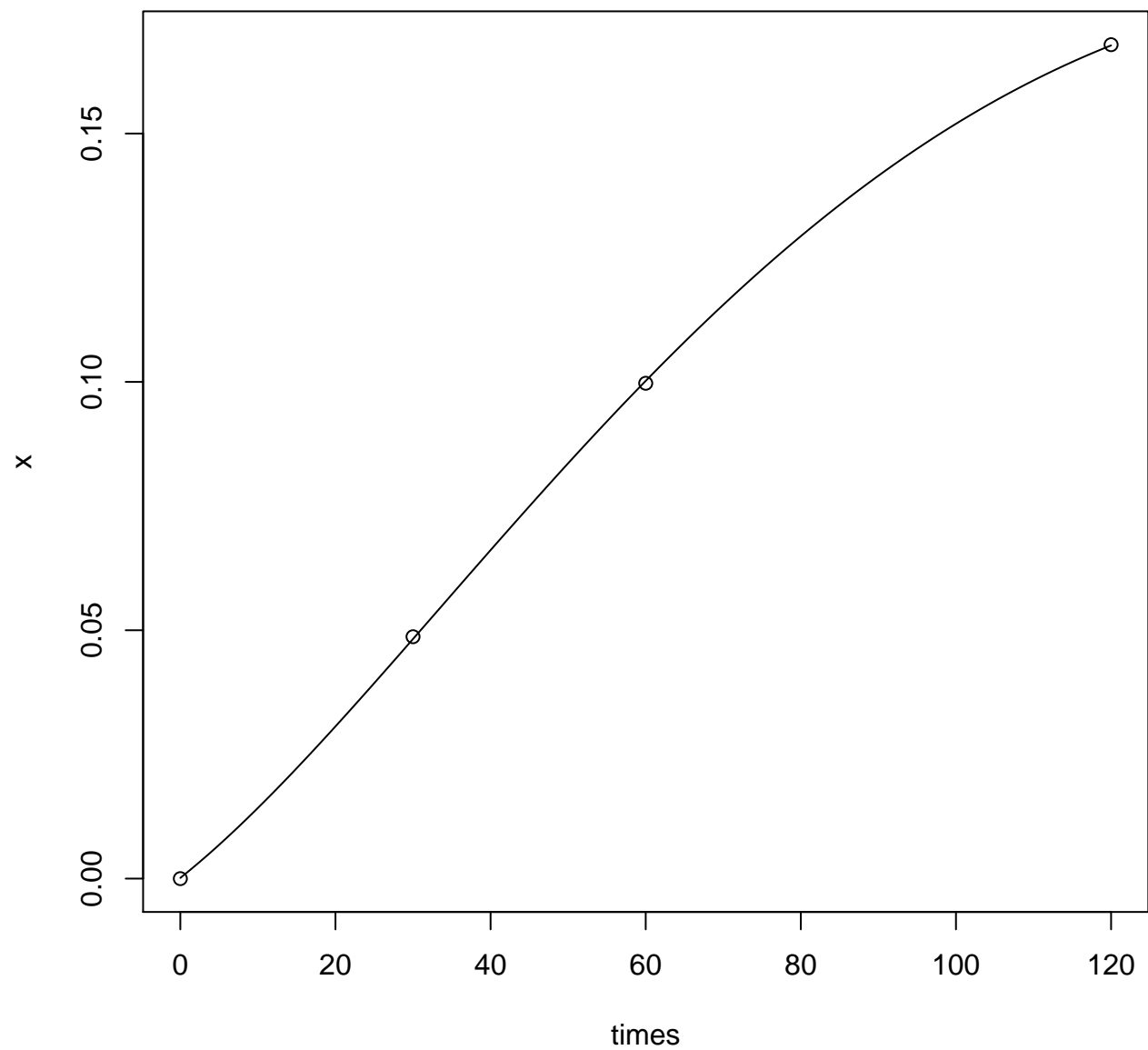

Supplement: Supplementary file 1 [file metabolites-10-00150-s001.zip › Supplementary Code S1/Glu-Pro/CL7/fit_input/res_fit_enr_CL7_glu_1.pdf]

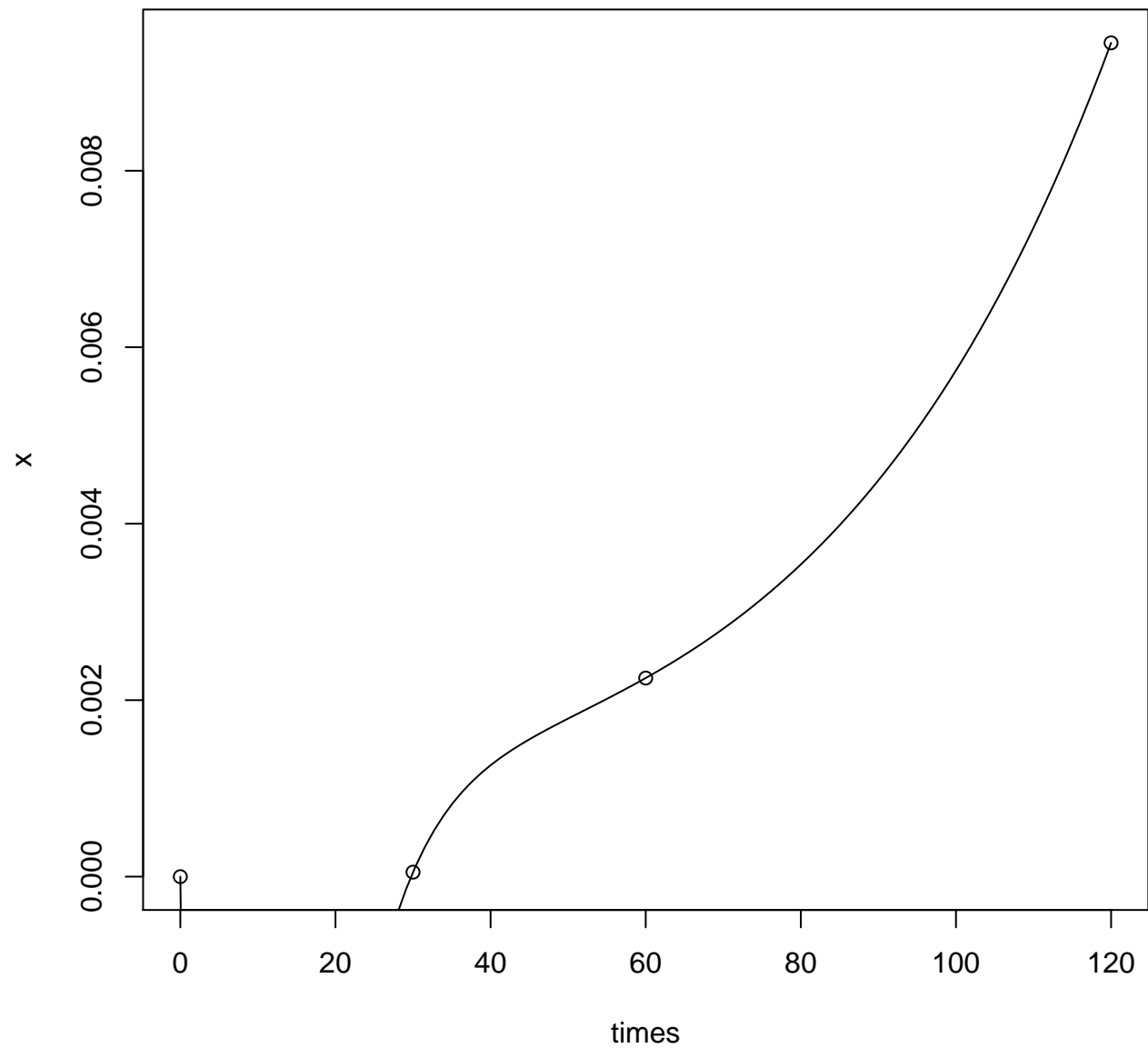

Supplement: Supplementary file 1 [file metabolites-10-00150-s001.zip › Supplementary Code S1/Glu-Pro/CL7/fit_input/res_fit_enr_CL7_pro_1.pdf]

## fitting results

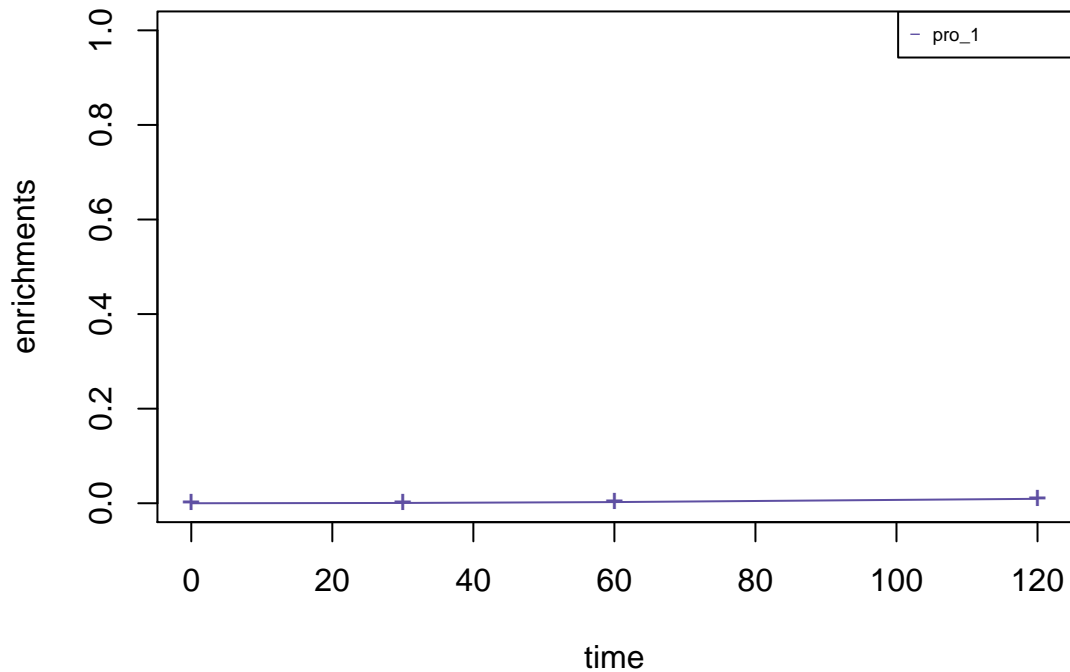

Supplement: Supplementary file 1 [file metabolites-10-00150-s001.zip › Supplementary Code S1/Glu-Pro/CL7/fit_subnet_fitpro/results.pdf]

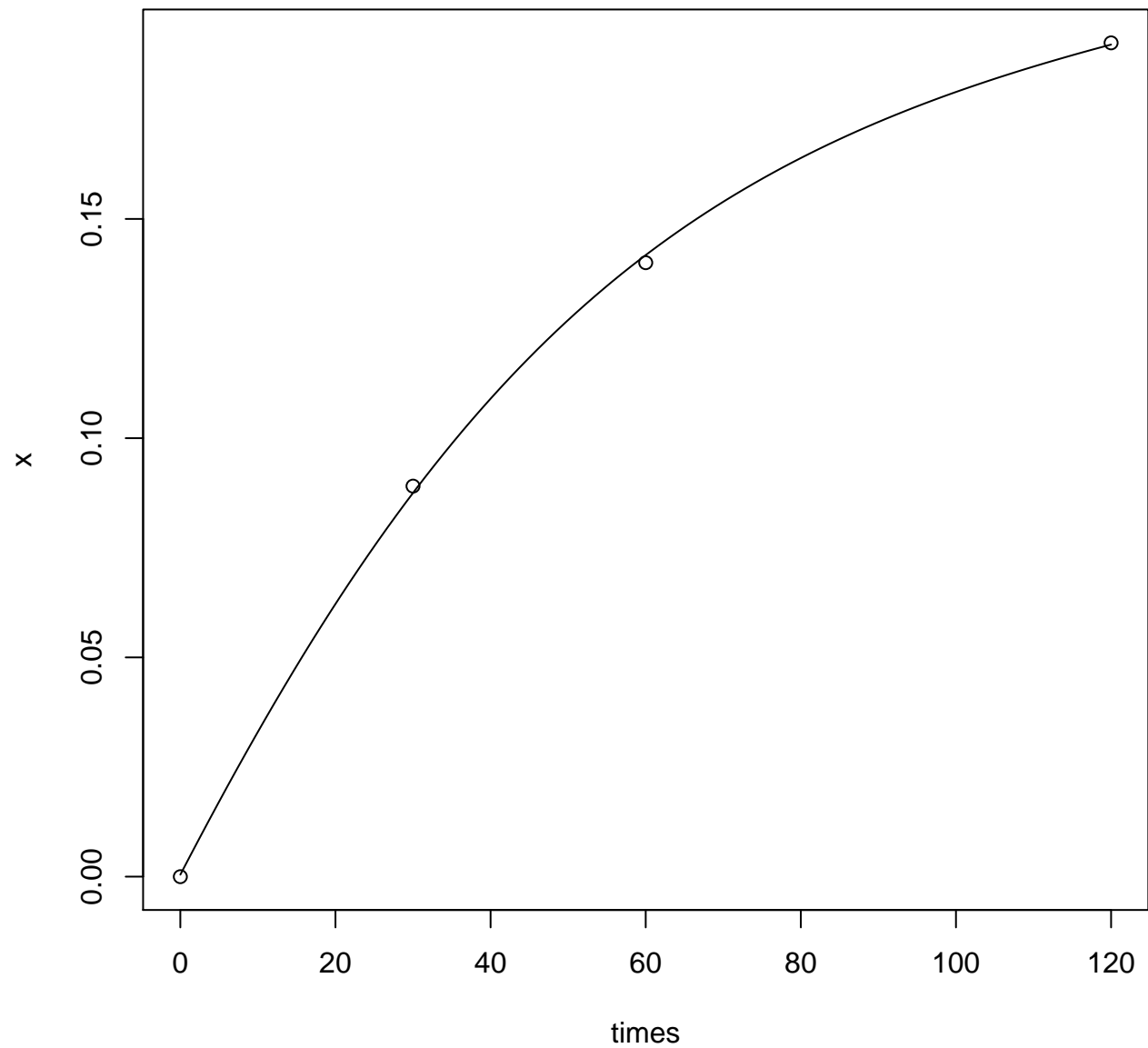

Supplement: Supplementary file 1 [file metabolites-10-00150-s001.zip › Supplementary Code S1/Glu-Val/AL11/fit_input/res_fit_enr_AL11_glu_1.pdf]
