# Supplementary material for: Sink/Source Balance of Leaves Influences Amino Acid Pools and Their Associated Metabolic Fluxes in Winter Oilseed Rape (Brassica napus L.)
Source: Metabolites. 2020 Apr 13;10(4):150. doi: 10.3390/metabo10040150 (PMC7240945; doi:10.3390/metabo10040150)
Supplement: Supplementary file 1 [file metabolites-10-00150-s001.zip › Supplementary Code S1/Glu-Val/AL3/fit_input/res_fit_enr_AL3_val_1.pdf]

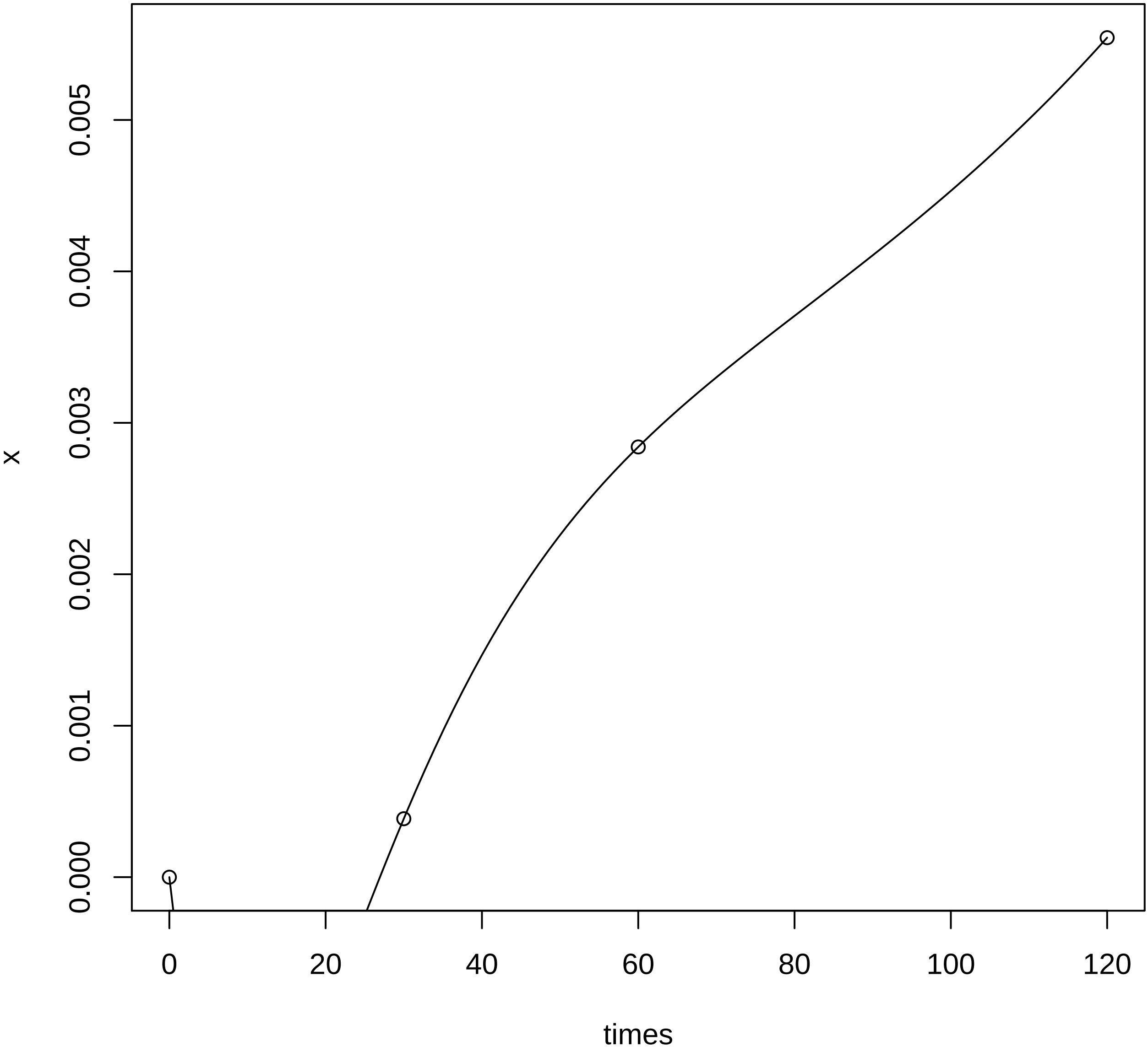

0.000
0.001
0.002
0.003
0.004
0.005
x
0
20
40
60
80
100
120
times
