# Supplementary material for: Sink/Source Balance of Leaves Influences Amino Acid Pools and Their Associated Metabolic Fluxes in Winter Oilseed Rape (Brassica napus L.)
Source: Metabolites. 2020 Apr 13;10(4):150. doi: 10.3390/metabo10040150 (PMC7240945; doi:10.3390/metabo10040150)
Supplement: Supplementary file 1 [file metabolites-10-00150-s001.zip › Supplementary Code S1/Glu-Val/CL11/fit_subnet_fitval/results.pdf]

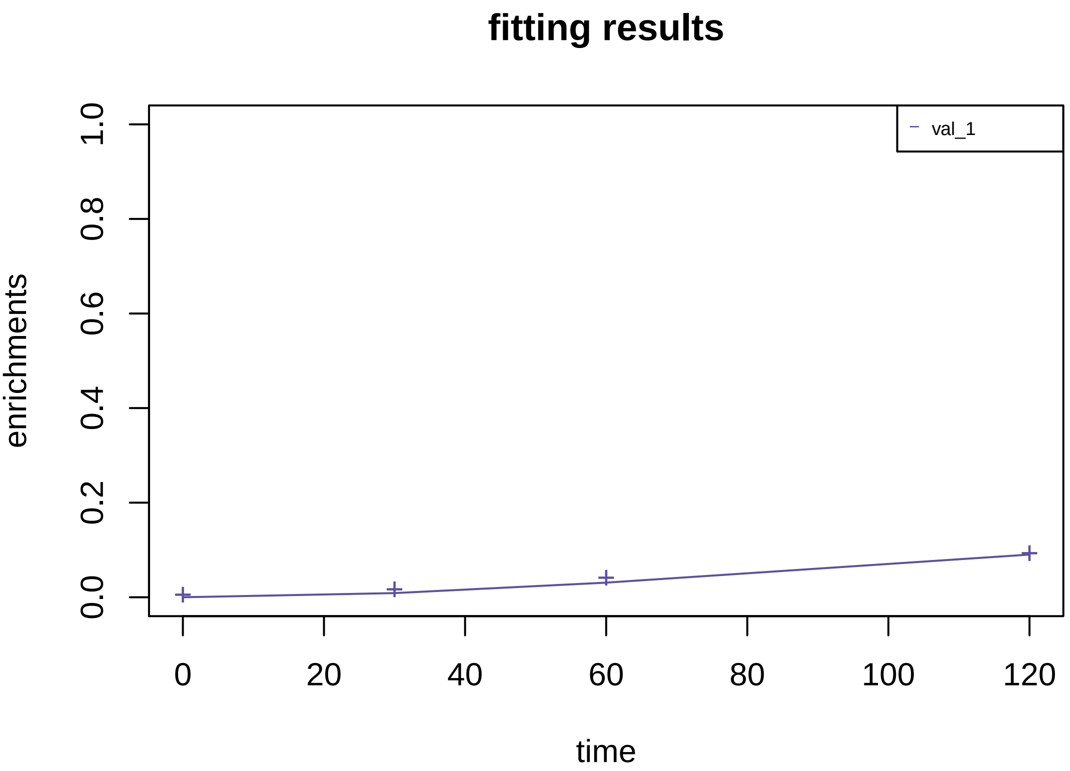
fitting results
val_1
enrichments
0.0
0.2
0.4
0.6
0.8
1.0
0
20
40
60
80
100
120
time
